# DRP1 interacts directly with BAX to induce its activation and apoptosis

**DOI:** 10.15252/embj.2021108587

**Published:** 2022-01-13

**Authors:** Andreas Jenner, Aida Peña‐Blanco, Raquel Salvador‐Gallego, Begoña Ugarte‐Uribe, Cristiana Zollo, Tariq Ganief, Jan Bierlmeier, Markus Mund, Jason E Lee, Jonas Ries, Dirk Schwarzer, Boris Macek, Ana J Garcia‐Saez

**Affiliations:** ^1^ Institute for Genetics CECAD University of Cologne Cologne Germany; ^2^ Interfaculty Institute of Biochemistry University of Tübingen Tübingen Germany; ^3^ Interfaculty Institute of Cell Biology University of Tübingen Tübingen Germany; ^4^ Cell Biology and Biophysics Unit European Molecular Biology Laboratory Heidelberg Germany; ^5^ University of Colorado Boulder CO USA

**Keywords:** BCL‐2 proteins, fluorescence correlation spectroscopy, membrane protein complex, mitochondrial division, super‐resolution microscopy, Autophagy & Cell Death, Membranes & Trafficking, Organelles

## Abstract

The apoptotic executioner protein BAX and the dynamin‐like protein DRP1 co‐localize at mitochondria during apoptosis to mediate mitochondrial permeabilization and fragmentation. However, the molecular basis and functional consequences of this interplay remain unknown. Here, we show that BAX and DRP1 physically interact, and that this interaction is enhanced during apoptosis. Complex formation between BAX and DRP1 occurs exclusively in the membrane environment and requires the BAX N‐terminal region, but also involves several other BAX surfaces. Furthermore, the association between BAX and DRP1 enhances the membrane activity of both proteins. Forced dimerization of BAX and DRP1 triggers their activation and translocation to mitochondria, where they induce mitochondrial remodeling and permeabilization to cause apoptosis even in the absence of apoptotic triggers. Based on this, we propose that DRP1 can promote apoptosis by acting as noncanonical direct activator of BAX through physical contacts with its N‐terminal region.

## Graphical abstract

Dynamin‐like protein DRP1 can promote apoptosis by inducing activation and mitochondrial translocation of apoptotic executioner protein BAX.

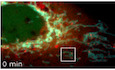

## Introduction

Apoptosis is a form of programmed cell death that plays a key role in fundamental biological processes like embryo development, tissue homeostasis, and the correct functioning of the immune system. Dysregulation of apoptosis has been related with human pathology, including neurodegenerative diseases and cancer (Strasser *et al*, 2011). Apoptosis execution is mediated by the apoptotic caspases, which accelerate cell death by cleaving a defined set of target proteins that leads to the organized dismantling of the cellular components. In most cells, activation of the caspase cascade requires mitochondrial outer membrane permeabilization (MOMP), which releases cytochrome *c* (cyt *c*) and Smac/DIABLO into the cytosol (Bock & Tait, 2020). MOMP is considered the point of no return in the cell’s commitment to death, as cells where all mitochondria underwent MOMP ultimately die also in the absence of caspase activity (Tait *et al*, 2010). Besides MOMP, mitochondria undergo multiple alterations during apoptosis including cristae remodeling, extensive fragmentation, changes in lipid composition and calcium signaling, loss of mitochondrial potential, and swelling (Cosentino & Garcia‐Saez, 2014). Furthermore, recent studies reported the release of mitochondrial DNA (mtDNA) into the cytosol during apoptosis, which happens via extrusion of the mitochondrial inner membrane (MIM) through the mitochondrial outer membrane (MOM) and initiates type I interferon inflammatory responses normally blocked by caspase activity (McArthur *et al*, 2018; Riley *et al*, 2018).

BAX is a pro‐apoptotic member of the BCL‐2 family of proteins that, together with its homolog protein BAK, is necessary for the execution of MOMP and the additional mitochondrial alterations in apoptosis (Pena‐Blanco & Garcia‐Saez, 2018). BAX is kept in an inactive form that constantly retrotranslocates between cytosol and mitochondria in healthy cells (Edlich *et al*, 2011; Schellenberg *et al*, 2013). Upon apoptosis induction, BAX is activated by interaction with the BH3 domain of the direct activator BH3‐only proteins, like tBID and BIM, which induce BAX accumulation at discrete puncta at the MOM, called apoptotic foci (Karbowski *et al*, 2002). This is accompanied by conformational changes that allow extensive membrane interactions, dimerization, and further self‐assembly of BAX into multiple oligomeric species (Subburaj *et al*, 2015). BAX oligomers form supramolecular structures shaped as lines, arcs, and rings, and both arcs and rings have been associated with growing membrane pores at the MOM that reach sizes in the order of hundreds of nanometers in diameter (Große *et al*, 2016; Salvador‐Gallego *et al*, 2016). These pores are responsible for the release of mitochondrial contents to the cytosol, ranging from cyt *c* and Smac to mtDNA (McArthur *et al*, 2018; Riley *et al*, 2018). Despite the clear paramount role of BAX and BAK in MOMP, the contribution of additional mitochondrial proteins to this process remains an open question.

Mitochondrial fragmentation is conserved in apoptotic cell death, even in organisms that do not involve MOMP (Martinou & Youle, 2011), yet its relevance for cell death is poorly understood. The dynamin‐like protein DRP1 promotes mitochondrial fission in healthy human cells to maintain cellular homeostasis, and it mediates mitochondrial fragmentation and participates in cristae remodeling in apoptotic human cells to facilitate cyt *c* release (Frank *et al*, 2001; Otera *et al*, 2016). DRP1 is SUMOylated during apoptosis, which stabilizes its oligomeric form at mitochondria to stabilize membrane contacts sites between ER and mitochondria (Prudent *et al*, 2015). DRP1 co‐localizes with BAX at apoptotic foci in mitochondria (Karbowski *et al*, 2002) and several lines of evidence support an interplay between the two proteins in apoptosis. DRP1 has been shown to promote the oligomerization of BAX in *in vitro* reconstituted systems by promoting negative membrane curvature (Montessuit *et al*, 2010). However, the contribution of DRP1 to apoptosis is controversial, because mitochondrial fission can be uncoupled from cyt *c* release and cells deficient in DRP1 also undergo cell death albeit with altered kinetics (Parone *et al*, 2006; Estaquier & Arnoult, 2007; Sheridan *et al*, 2008). As a result, the molecular mechanisms and functional significance of the connection between DRP1 and BAX in apoptosis remain unclear.

Here we show that direct interaction between BAX and DRP1 is induced at apoptotic foci in correlation with MOMP and maintained until the death of the cell. The association between BAX and DRP1 requires the lipid environment and affects the membrane activity of both proteins. We identify several surfaces of BAX involved in the interaction with DRP1 and determine that the N‐terminal region of the protein is required for the association with DRP1 in cells. Interestingly, forced interaction between BAX and DRP1 induces their translocation to mitochondria, accumulation in apoptotic foci, as well as their activation for MOMP, and mitochondrial remodeling leading to apoptosis. Our findings provide a molecular basis for the functional link between the machineries for apoptosis execution and for mitochondrial dynamics and reveal that DRP1 can act as a non‐BH3 activator of BAX to promote apoptosis.

## Results

### Apoptosis induction brings BAX and DRP1 in close proximity in correlation with MOMP

**Figure 1:**
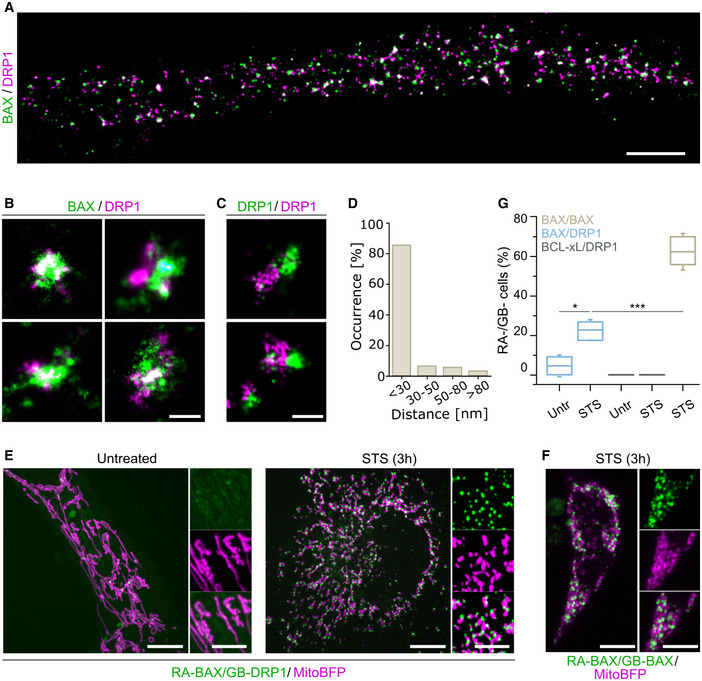
BAX and DRP1 specifically interact at mitochondria during apoptosis A–DDual‐color SMLM shows colocalization of BAX and DRP1 at the nanoscale. (A) Overview SMLM image of a HeLa cell transfected with GFP‐BAX stained with anti‐GFP nanobody‐AF647 and anti‐DRP1 antibody probed with a CF680‐labeled secondary antibody, 3 h after apoptosis induction. Scale bar 2 μm. (B) Magnified SMLM images of GFP‐BAX and DRP1 assemblies colocalizing up to 30 nm during apoptosis. Scale bar 100 nm. (C) Colocalization control of DRP1 immunostaining with the same primary and different secondary antibodies (labelled with AF647 and CF680) in HeLa cells. Scale bar 100 nm. (D) Quantification of the distance from GFP‐BAX to DRP1 structures (distance measured at the center of each structure) from dual‐color SMLM images. Data are quantified from *n* = 4 independent experiments with a total of 720 BAX structure.E, FRepresentative confocal microscopy images of HeLa cells transfected with ddFP RA‐BAX and GB‐DRP1 (E) in untreated or apoptotic (STS) conditions, or with RA‐BAX and GB‐BAX during apoptosis (F). RA‐BAX/GB‐DRP1 and RA‐BAX/GB‐BAX complexes shown in green, mitochondria labeled with mito‐BFP in magenta. Scale bar 10 μm. Right panels are zoomed areas representing individual and merged channels. Scale bar 5 μm.GBAX/DRP1 interaction compared to BAX/BAX and BCL‐xL/DRP1 by ddFP. % mito‐BFP transfected cells that show RA/GB signal was quantified in HeLa cells untreated (untr.) and 3 h after apoptosis induction (STS). Box plots represent the interquartile (box), median (line) and SD (whiskers) of *n* = 3 independent experiments (with *n* = 100 cells each). Levels of significance were determined by paired two‐tailed Student's *t*‐test (*P < 0.05, ***P < 0.001) compared to BAX/DRP1 after apoptotic induction (STS).

While we and others have so far not been able to detect direct interaction between endogenous BAX and DRP1 (Montessuit *et al*, 2010), these two proteins clearly co‐localize at discrete foci in apoptotic cells visualized with confocal microscopy (Karbowski *et al*, 2002). However, the spatial resolution limit of around 200 nm does not allow to discern whether BAX and DRP1 are connected by direct physical interactions. To gain further insight into the structural organization of BAX and DRP1 during apoptosis, we took advantage of the high spatial resolution offered by single‐molecule localization microscopy (SMLM) (Salvador‐Gallego *et al*, 2016). We imaged fixed HeLa cells transiently transfected with GFP‐BAX 3 h after staurosporine (STS) treatment for apoptosis induction and stained them with anti‐GFP nanobodies labeled with Alexa Fluor (AF)647. Endogenous DRP1 was immunostained using a secondary antibody labeled with the cyanine‐based fluorescent (CF) dye CF680. Using spectral unmixing based on a ratiometric classification of the individual localizations in the individual emission channels (Winterflood *et al*, 2015), we built two‐color super‐resolved images that revealed a close apposition between the fluorescent signals corresponding to BAX and DRP1, which appeared in discrete foci (Fig 1A). The degree of overlap was comparable to that of the positive co‐localization control based on DRP1 immunostaining with two secondary antibodies labeled with AF647 and CF680 dyes, indicating that in apoptotic cells BAX and DRP1 co‐localize up to a resolution of 30 nm (Fig 1B and C). In agreement with this, quantification of the distance between BAX and DRP1 in the foci revealed that the distance between them was < 30 nm in > 80% of the cases (Fig 1D).

**Figure EV1:**
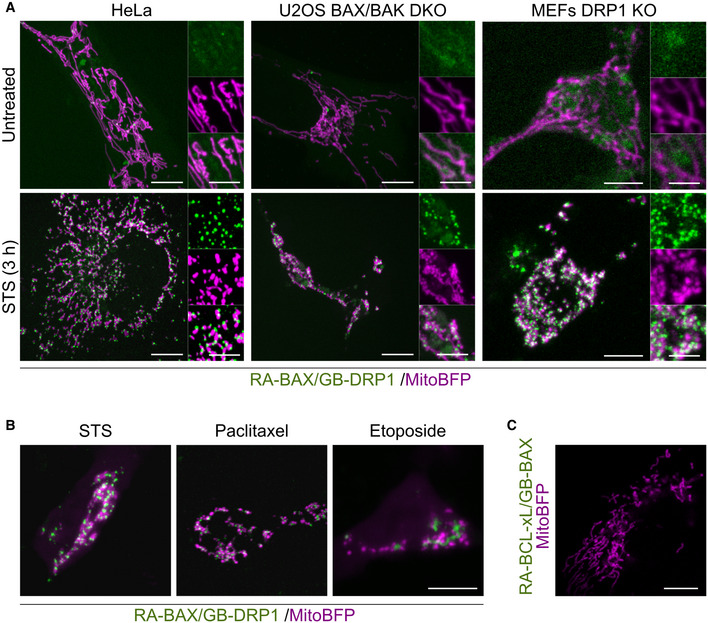
Interaction of BAX and DRP1 is independent of cell type and apoptotic stimuli Representative confocal microscopy images of ddFP of RA‐BAX and GB‐DFP1 (green) in untreated or apoptotic (STS) HeLa (same images as used in Fig 1E), U2OS BAX/BAK DKO, and MEF DRP1 KO cells. Mitochondria are visualized using mito‐BFP (magenta). Scale bar 10 μm. Right panels are zoomed areas representing individual and merged channels. Scale bar 5 μm.Representative confocal microscopy images of RA‐BAX/GB‐DRP1 (green) and mito‐BFP (magenta) overexpressed in U2OS BAX/BAK DKO cells after apoptosis induction using STS, Paclitaxel or Etoposide as indicated. Scale bar 10 μm.Representative confocal microscopy image of RA‐BCL‐xL/GB‐DRP1 (green) and mito‐BFP (magenta) overexpressed in U2OS BAX/BAK DKO cells after apoptosis induction using STS. Scale bar 10 μm. Images are representative for *n* = 3 independent experiments. Data information: Images are representative for *n* = 3 independent experiments.

Since both BAX and DRP1 are known to form large oligomers with sizes in the order of the measured distances between them by SMLM, we reasoned that they were likely to interact physically at the apoptotic foci. To test this hypothesis, we used the dimerization‐dependent fluorescent protein (ddFP) technique (Ding *et al*, 2015). In this method, the proteins of interest are tagged with the fusion proteins RA and GB, respectively, which emit significant measurable fluorescence only when they are part of the same complex (< 10 nm apart). Compared to similar approaches, the ddFP pair offers the advantage that it does not enhance association between the tagged proteins and can be used to follow the dynamics of reversible interactions (Ding *et al*, 2015). We transiently expressed RA‐BAX and GB‐DRP1 in HeLa cells and imaged cells before and after apoptosis induction with STS for 3 h (optimal treatment for BAX translocation to mitochondrial foci) (Salvador‐Gallego *et al*, 2016). As shown in Fig 1E, the fluorescence of RA‐BAX/GB‐DRP1 complexes was negligible in untreated cells, but became apparent as discrete foci in apoptotic cells with a distribution similar to that of colocalized BAX and DRP1 in Fig 1A and B. These results were consistent when we used different cell lines (U2OS BAX/BAK DKO and MEFs DRP1 KO) or different apoptotic triggers (paclitaxel and etoposide) (Fig EV1A and B). As a positive control, RA‐BAX/GB‐BAX interaction signal was also induced in apoptosis (Fig 1F). In contrast, the combination of the anti‐apoptotic BCL‐2 protein BCL‐xL tagged with RA and GB‐DRP1 did not give any fluorescent signal neither in untreated nor in treated cells (Fig EV1C). Quantification of the % cells with positive RA/GB signal revealed that the interaction between BAX and DRP1 was enhanced in apoptotic cells compared to untreated cells (Fig 1G).

**Figure 2:**
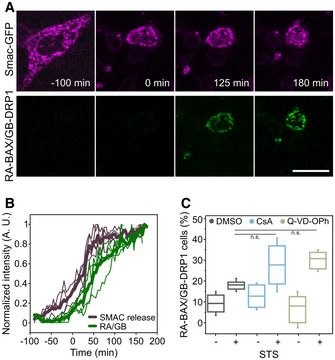
Dynamics of BAX and DRP1 interaction during apoptosis Confocal microscopy time‐series images of the increase in cytosolic Smac‐GFP (magenta) in relation to the detection of RA‐BAX/GB‐DRP1 complexes (green) during apoptosis induction in MEF DRP1 KO cells. Scale bar 10 μm. Images are representative for *n* = 3 independent experiments.Normalized fluorescence intensity of Smac‐GFP in a region of interest in the cytosol and of RA‐BAX/GB‐DRP1 foci for individual cells (thin lines) and the average of all cells (thick lines, *n* = 5). Time 0 min corresponds to the time point when RA‐BAX/GB‐DRP1 foci were detected.Quantification of BAX/DRP1 interaction (% cells with RA‐BAX/GB‐DRP1 foci normalized to the mito‐BFP positive cells, *n* = 100 cells per experiment) during inhibition of effector caspases (Q‐VD‐OPh), the mitochondrial permeability transition pore (CsA) versus control (DMSO), with or without apoptosis induction (+/− STS). Box plots represent the interquartile (box), median (line) and SD (whiskers). Significance was determined from *n* = 3 independent experiments (with *n* = 100 cells each) by paired two‐tailed Student's *t*‐test (n.s. indicating *P* > 0.05) compared to DMSO control after apoptotic induction (STS).

To examine the dynamics of association between BAX and DRP1 during apoptosis progression, we performed live cell confocal imaging of HeLa cells transiently expressing RA‐BAX, GB‐DRP1, and Smac‐GFP following STS treatment. Smac is a mitochondrial pro‐apoptotic factor that is released to the cytosol upon MOMP. As shown in Fig 2A and B, the interaction between RA‐BAX and GB‐DRP1 was enhanced upon apoptosis induction and correlated in time with Smac‐GFP release into the cytosol, persisting until cell death. Of note, we observed a delay between the redistribution of Smac‐GFP and the appearance of BAX/DRP1 complexes, which was of about 10 min at the 50% increase in both signals. This is comparable to that observed between the formation of GFP‐BAX foci and Smac release during apoptosis (Salvador‐Gallego *et al*, 2016) and likely related to their relative efficiency of detection. Due to this technical limitation, and although we observe a clear temporal correlation, we cannot exclude that one event happens before the other from these experiments.

To discard that the interaction between BAX and DRP1 was not a product of downstream caspase activity or of the permeability transition pore (PTP), we confirmed that the signal was maintained when cells were treated with a pan‐caspase inhibitor (Q‐VD‐OPh) or with the PTP blocker cyclosporine A (CsA) (Fig 2C). Together, these findings demonstrate that, specifically during apoptosis, BAX and DRP1 associate into complexes that accumulate at discrete foci, correlate in time with MOMP, and persist until the death of the cell.

### Direct interaction between BAX and DRP1 requires the membrane and affects the activity of both proteins

**Figure 3:**
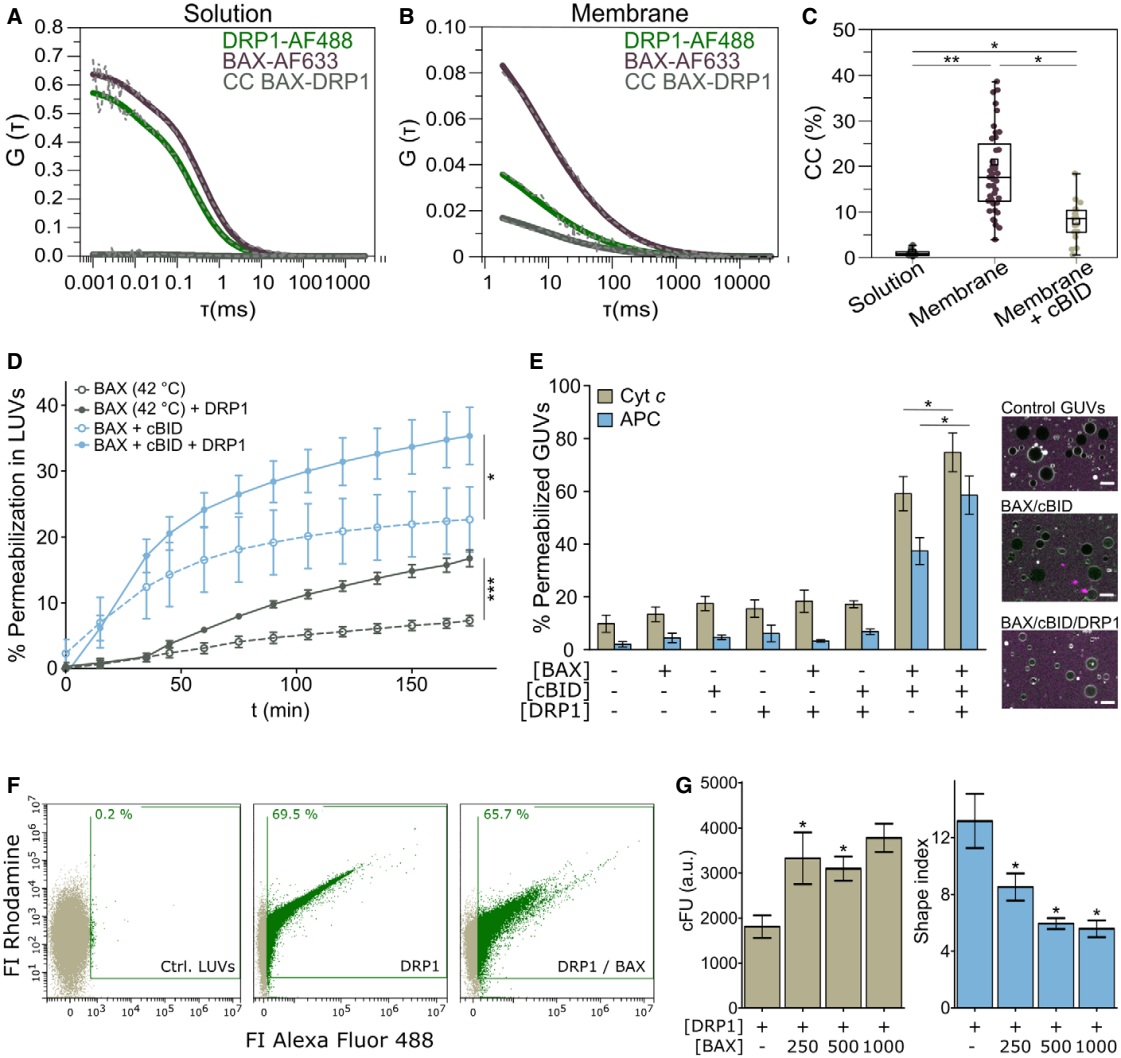
Direct interaction of BAX and DRP1 in the membrane affects their respective activities A, BRepresentative auto‐ (green and violet curves) and cross‐correlation (CC, BAX‐DRP1, grey curves) curves of DRP1‐AF488 and BAX‐AF633 measured by FCCS in solution (A) and in the membrane of GUVs (B). Dash gray line depicts raw data and solid lines correspond to data fitting.CQuantification of %CC between DRP1‐AF488 and BAX‐AF633 in solution (grey), in the membrane (violet), and in the membrane in presence of excess unlabeled cBID (beige). Box plots represent the interquartile (outer box), mean (inner box), median (line) and range (whiskers). Levels of significance were determined by paired two‐tailed Student's *t*‐test (*P < 0.05, **P < 0.01) from *n* = 9 measurements in solution, *n* = 46 individually measured GUVs in the membrane or *n* = 17 GUVs in presence of cBID.DEffect of DRP1 on BAX‐induced LUV permeabilization. BAX was activated by cBID (blue lines), or mild heat (42°C, grey lines). Data are presented as mean ± SD of *n* = 3 individual experiments. Significance was determined at the end‐point of the kinetic measurement (180 min) by paired two‐tailed Student's *t*‐test (*P < 0.05, ***P < 0.001).EEffect of DRP1 on BAX‐induced GUV permeabilization. Left: % GUVs permeabilized to Cytochrome *c*_488_ (Cyt *c,* 12 kDa, beige) and allophycocianin (APC, 104 kDa, blue) in the absence or presence of cBID, BAX and DRP1 combined as indicated. Data are presented as mean ± SD of *n* = 4 independent experiments. *P < 0.05 (paired two‐tailed Student's *t*‐test). Right: representative confocal microscopy images showing GUVs (grey) in a solution of Cyt *c* (green) and APC (magenta). Scale bar 10 µm.F, GEffect of BAX on DRP1 membrane density and DRP1‐induced shape alterations of liposomes measured by flow cytometry. (F) Representative flow cytometry plots outlining DRP1 (Alexa Fluor 488 signal) binding to LUVs (Rhodamine signal) in the absence or presence of BAX. % DRP1‐positive LUVs indicated in green. (G) Membrane density of DRP1 (corrected fluorescence units, cFU, left graph) and DRP1‐induced membrane tethering (indicated by a shape index > 1, right graph) in LUVs in the absence or presence of different concentrations of BAX. Data are presented as mean ± SD of *n* = 3 independent experiments. *P < 0.05 (paired two‐tailed Student's *t*‐test) vs. DRP1 without BAX.

**Figure EV2:**
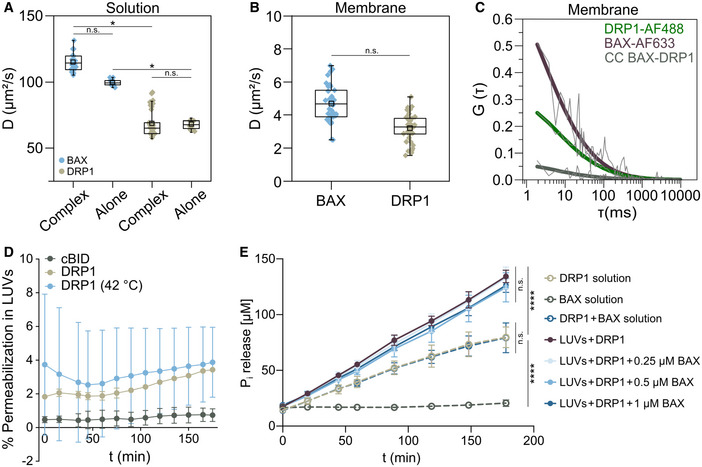
Interaction between BAX and DRP1 and activity analysis *in vitro* A, BDiffusion coefficients derived from FCCS analysis of BAX‐AF633 (blue) and DRP1‐AF488 (beige) in solution in complex with each other and alone as indicated (A) or in the membrane (B). Box plots (in A and B) represent the interquartile (outer box), mean (inner box), median (line) and range (whiskers). Levels of significance were determined by paired two‐tailed Student's *t*‐test (n.s. *P* > 0.05, *P < 0.05) from *n* ≥ 25 individual measurements for BAX or DRP1 in complexes and n ≥ 10 measurements of BAX and DRP1 alone in solution (A) or n ≥ 33 individual measurements for BAX or DRP1 in the membrane (B).CRepresentative auto‐ (green and violet curves) and cross‐correlation (CC, BAX‐DRP1, grey curves) curves of DRP1‐AF488 and BAX‐AF633 measured in the membrane of GUVs in presence of unlabeled cBID. Thin grey lines depict raw data and thick lines correspond to data fitting.DNegative control of LUV permeabilization by cBID and DRP1 alone. Calcein release was measured in calcein‐encapsulated LUVs incubated with cBID (grey) or DRP1 at room temperature (beige) or after mild heating (42°C, blue). Data are presented as mean ± SD of *n* = 3 independent experiments.EEffect of BAX on the GTPase activity of DRP1 in solution or in the membrane. Time course of GTP hydrolysis by DRP1 measured at 37°C in the absence or presence of LUVs and different concentrations of BAX as indicated. Data are presented as mean ± SD of *n* = 4 independent experiments. Significance was determined by paired two‐tailed Student's *t*‐test (n.s. *P* > 0.05, ****P < 0.0001).

To investigate whether BAX and DRP1 interact directly or whether additional components are required for their association, we quantified their interaction in minimal systems of chemically controlled composition. We performed Fluorescence Cross‐Correlation Spectroscopy (FCCS) measurements (Garcia‐Saez *et al*, 2009; Bleicken *et al*, 2017) using recombinant, fluorescently labeled BAX (BAX‐AF633) and DRP1 (DRP1‐AF488) (Fig 3A–C). FCCS is a technique with single molecule sensitivity that calculates the temporal auto‐correlation of diffusing BAX‐AF633 and DRP1‐AF488 particles, from which the diffusion coefficient is calculated (Fig EV2A and B). It also quantifies the cross‐correlation (CC) signal due to the codiffusion of BAX‐AF633/DRP1‐AF488 complexes, which is directly proportional to the extent of association between the two proteins. As shown in Fig 3A and C, we could not detect any CC between BAX‐AF633 and DRP1‐AF488 in solution. In contrast, when we measured FCCS on BAX‐AF633 and DRP1‐AF488 bound to Giant Unilamellar Vesicles (GUVs), we clearly detected a positive CC indicative of direct interaction between the proteins (Fig 3B and C). Of note, DRP1 bound spontaneously to GUVs with a simple lipid composition containing the mitochondrial lipid cardiolipin, while association of BAX with the membrane was not induced by DRP1 and was promoted by incubation at 42°C (heat activation). As a control to confirm that the interaction between BAX and DRP1 is specific and not an artifact due to accumulation of both proteins in the membrane, an excess of unlabeled cBID (a known BAX interactor (Czabotar *et al*, 2013)) successfully competed for the association between BAX‐AF633 and DRP1‐AF488 and decreased the %CC (Figs 3C and EV2C). These results demonstrate that BAX and DRP1 directly interact *in vitro* and that the membrane environment is the only additional component required for such interaction.

We then took advantage of chemically controlled, *in vitro* reconstituted systems to explore whether the interaction between BAX and DRP1 affects the activities that have been reported for both proteins. First, we used assays of calcein release from large unilamellar vesicles (LUVs) (Garcia‐Saez *et al*, 2005) to test the effect of DRP1 on the membrane‐permeabilizing activity of BAX. As shown in Fig 3D, DRP1 enhanced the pore‐formation activity of BAX activated with mild heat or with cBID. Consistent with these results, DRP1 also increased the BAX‐induced permeabilization of GUVs to large molecules like cytochrome *c* and the 100 kDa protein APC (Fig 3E) in the presence of cBID. None of the individual proteins, neither BAX/DRP1 alone were able to permeabilize vesicles (Figs 3E and EV2D). These findings suggest that DRP1 can only promote BAX pore activity when it is already bound to membranes, in agreement with their interaction exclusively in the lipid environment.

Next, we studied the effect of BAX on the reported DRP1 ability to hydrolyze GTP and to tether liposomes (Ugarte‐Uribe *et al*, 2014). Incubation with BAX did not alter the GTPase activity of DRP1 neither in solution nor in the presence of liposomes (Fig EV2E). Remarkably, the presence of BAX increased the density of DRP1 on the individual LUVs and its membrane‐tethering activity (characterized by decrease in the shape index), both in a concentration‐dependent manner (Fig 3F and G). These findings demonstrate that the direct interaction between BAX and DRP1 has functional consequences for the membrane activities that both proteins exhibit *in vitro*.

### Helices α2, α5, and α9 of BAX participate in the interaction with DRP1, although only the N‐terminus is required for their association in cells

**Figure 4:**
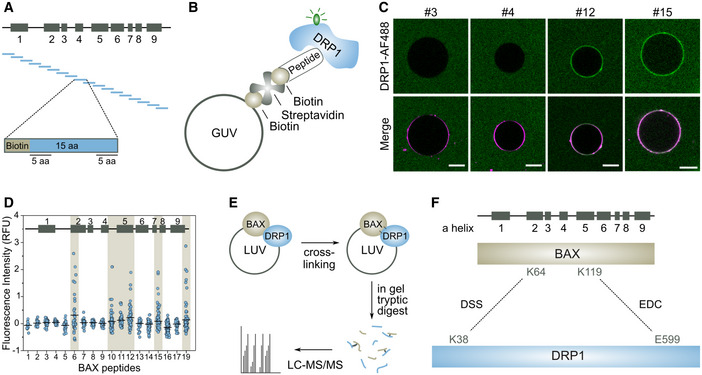
Interaction surfaces between BAX and DRP1 A–DBAX peptide array to define interaction site with DRP1. (A) Schematic representation of the peptide array corresponding to BAX secondary structure with boxes indicating BAX α‐helices 1‐9. BAX sequence was divided in peptides of 15 amino acids (aa) preceded by a biotin head. The last 5 aa of each peptide overlapped with the first 5 aa of the next peptide. (B) Graphical representation of the DRP1 binding assay. Biotin‐containing BAX peptides were attached to GUVs using biotinylated lipids and streptavidin. Binding between BAX peptides and DRP1‐AF488 results in a recruitment of DRP1‐AF488 to the GUV membrane indicated by AF488 fluoresce detected at the rim of the GUV. (C) Representative images of GUVs incubated with DRP1‐AF488, streptavidin and the corresponding BAX peptides (#3, #4, #12, #15). Scale bar 10 µm. (D) Quantification of the relative fluorescence intensity of DRP1‐AF488 at the rim of the GUVs for each BAX peptide of *n* = 3 individual experiments (with *n* = 100 vesicles each). Black lines indicate the mean value. Interacting BAX peptides are highlighted in beige.E, FProtein crosslinking coupled with mass spectrometry of BAX and DRP1 in liposomes. (E) BAX/DRP1 crosslinking analysis workflow. After incubation with crosslinking reagents, proteins were digested using trypsin subjected to LC‐MS/MS analysis. (F) Schematic representation of BAX and DRP1 with detected cross‐linked residues connected by dash lines using DSS and EDC cross‐linkers as indicated.

To support our data that the interaction between BAX and DRP1 is specific and functionally relevant also in cells, we sought to identify the regions in BAX that are involved in the interaction with a combination of orthogonal approaches. To this aim, we used first a peptide array covering the full sequence of BAX as in Alsop *et al*, 2015; Iyer *et al* (2016). Each peptide was 15 residues long and overlapped with the neighboring peptides by 5 amino acids in the N‐terminus and 5 amino acids in the C‐terminus (Fig 4A). Biotin was added at the N‐terminus of each peptide. Since our FCCS data suggested that BAX and DRP1 interact only in the membrane, we implemented an assay based on GUVs with a lipid composition that does not support spontaneous binding of DRP1 and that contains biotinylated lipids and the fluorescent dye DiD. We mixed each of the biotinylated peptides of the BAX array with GUVs in presence of streptavidin to induce the association of the peptide with the membrane and added DRP1‐AF488 (Fig 4B). We imaged the samples by confocal microscopy after 1 h incubation. Remarkably, some of the BAX peptides, but not all of them, promoted binding of DRP1‐AF488 to the membrane, which was evident by the increase in green fluorescence contrast at the vesicle rim (Fig 4C). By comparing the DRP1‐AF488 fluorescence on the GUV membranes in the different peptide samples, we found that the peptides corresponding to the beginning of α2, the regions containing α5 and α7, as well as the C‐terminal anchor α9 in BAX were capable of recruiting DRP1‐AF488 to the membrane (Fig 4D).

**Figure EV3:**
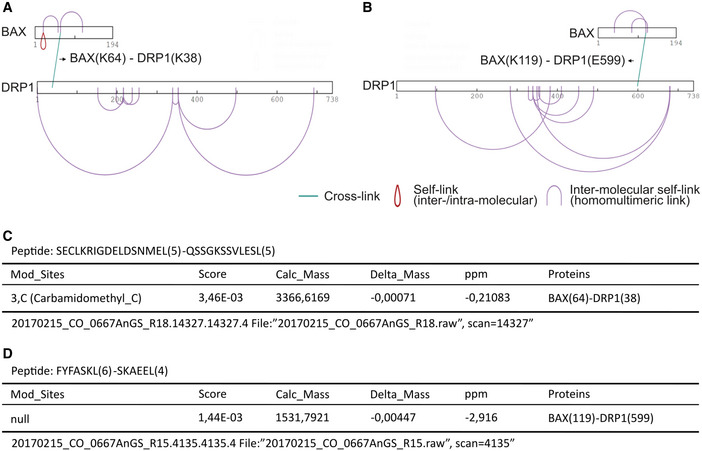
pLink analysis of all crosslinks identified by XL‐MS for BAX and DRP1 interaction in liposomes A, BGraphic representation of all positively identified crosslinks in the BAX/DRP1 complex mediated by DSS (A) and EDC (B).C, DExcerpt from the pLink search result for the BAX(64)‐DRP1(38) crosslinked by DSS (C) and BAX(K119)‐DRP1(K599) crosslinked by EDC (D).

In a complementary approach, we used chemical cross‐linking of BAX and DRP1 embedded in LUVs coupled with mass spectrometry analysis (Fig 4E) (O'Reilly & Rappsilber, 2018). Comparative analysis of the samples incubated with the cross‐linkers EDC and DSS revealed significant cross‐link sites with DRP1 at Lysines 64 and 119 of BAX, which are located at helices α2 and α5 (Figs 4F and EV3), in excellent agreement with the data obtained in the peptide array. Of note, the lack of Lysine residues after helix α5 of BAX does not allow reaction of this part of the protein with the cross‐linkers used.

**Figure 5:**
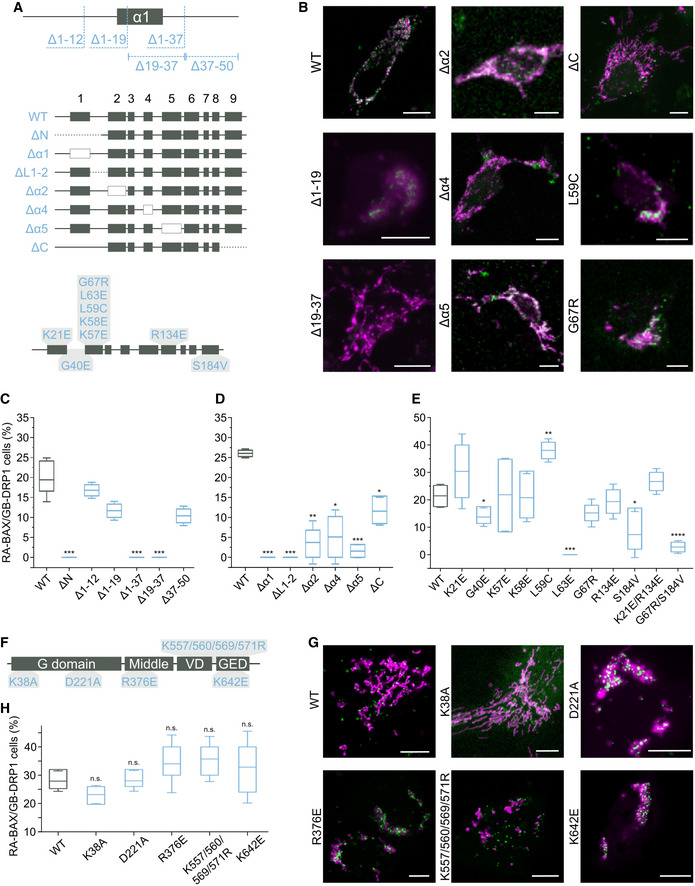
Regions required for interaction between BAX and DRP1 in apoptotic cells ASchematic representation of RA‐BAX mutant and deletion variants analyzed for the interaction with GB‐DRP1. The variants are displayed in the corresponding region of BAX secondary structure (boxes correspond to BAX helices α1‐9 as indicated).BRepresentative confocal microscopy images of U2OS BAX/BAK DKO cells of the interactions between RA‐BAX variants and GB‐DRP1, shown as green signal, 3 h after apoptosis induction. Mitochondria labeled with mito‐BFP in magenta. Scale bar 10 μm.C–EQuantification of the interaction of DRP1 with variants of BAX based on the % cells with detectable RA‐BAX/GB‐DRP1 fluorescence signal (interacting cells) normalized to the number of mito‐BFP positive cells. Box plots represent the interquartile (box), median (line) and SD (whiskers) of *n* = 3 independent experiments (with *n* = 100 cells each). Levels of significance were determined by paired two‐tailed Student's *t*‐test (*P < 0.05, **P < 0.01, ***P < 0.001, ****P < 0.0001) compared to BAX wild type (WT).FSchematic representation of GB‐DRP1 mutant variants analyzed for the interaction with RA‐BAX. The variants are displayed in the domain distribution of DRP1 (amino acid positions correspond to DRP1 isoform 3).GRepresentative confocal microscopy images of U2OS BAX/BAK DKO cells of the interactions between RA‐BAX and GB‐DRP1 variants, shown as green signal, 3 h after apoptosis induction. Mitochondria labeled with mito‐BFP in magenta. Scale bar 10 μm.HQuantification of the interaction of DRP1 variants with BAX based on the % cells with detectable RA‐BAX/GB‐DRP1 fluorescence signal (interacting cells) normalized to the number of mito‐BFP positive cells. Box plots represent the interquartile (box), median (line) and SD (whiskers) of *n* = 3 independent experiments (with *n* = 100 cells each). Significance was tested using paired two‐tailed Student's *t*‐test (n.s. *P* > 0.05) compared to DRP1 WT.

We then used this information to design deletion and point mutation variants of BAX that are deficient for interaction with DRP1 measured with the ddFP assay in cells (Fig 5A–E). Interestingly, deletion of the N‐terminal region of BAX (amino acids 1 to 50) completely abolished the association with DRP1, which we could narrow down to deletion of residues 19–37 (Δ19–37), which includes helix α1 and the loop to helix α2 (Fig 5C). In contrast, deletion of the C‐terminal helix α9 of BAX did not affect binding to DRP1, despite the ability of this region to recruit DRP1 in the assay with the peptide array (Fig 5D).

We then removed individually helices α1, α2, α4, and α5, as well as the loop between helices α1 and α2 (ΔL1–2) in BAX and analyzed the interaction with DRP1 by ddFP. Deletion of α1 or of the loop between α1 and α2 fully abrogated binding with DRP1, but not of α4 or α5, which presented reduced interaction (Fig 5D). Since the BH3 domain of BAX is located in α2, we then designed point mutations reported to block homo‐ and hetero‐dimerization of BAX with other BCL‐2 proteins. Only the exchange L63E disrupted the interaction with DRP1, but not any of the functionally relevant positions within the BH3 tested (Fig 5E), suggesting that the BH3 domain of BAX does not have the same role in the interaction with DRP1 or with other BCL‐2 proteins.

**Figure EV4:**
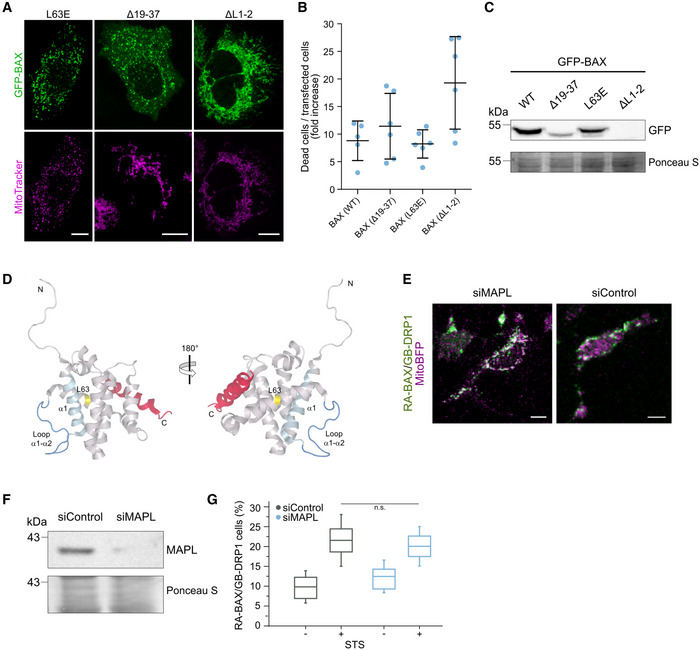
Expression, localization and apoptotic activity of BAX mutants not interacting with DRP1 and effect of MAPL‐mediated DRP1 SUMOylation on the interaction Confocal microscopy images of U2OS BAX/BAK DKO cells transfected with GFP‐BAX mutants L63E, Δ19–37 or ΔL1–2 as indicated (green) and stained with MitoTracker Deep Red FM to visualize mitochondria (magenta). Scale bar 10 µm. Images are representative for *n* = 3 independent experiments.Quantification of cell death induced by overexpression of GFP‐BAX mutants L63E, Δ19–37 and ΔL1–2 compared to wild type (WT) in U2OS BAX/BAK DKO cells. Data are presented as fold increase of dead cells 12 h after transfection normalized to the number of dead cells at the time point of transfection. Values correspond to mean (line) ± SD of *n* = 2 independent experiments. Single data points represent individual measurements from technical and biological replicates.Representative western blot analysis (*n* = 3 independent experiments) of total cell lysates from U2OS BAX/BAK DKO cells overexpressing GFP‐BAX mutants (as indicated) probed against GFP. Whole cell protein staining (Ponceau S) is shown to confirm equal loading.Structural model of BAX highlighting proposed interaction regions for DRP1 and for the BH3 domain of BID. BAX structure is based on the NMR structure of the inactive, full‐length monomer, PDB 1F16. In red, the C‐terminal helix of BAX occupying the hydrophobic groove, which corresponds to the binding site of the BH3 helix of BID. The regions corresponding to residues 19‐37 (in helix 1, light blue), the loop between helices 1 and 2 (blue) and L63 (yellow) are required for the interaction with DRP1.Confocal microscopy image of apoptotic U2OS BAX/BAK DKO cells transfected with RA‐BAX/GB‐DRP1 (green) and mito‐BFP (magenta) after siRNA‐mediated depletion of MAPL (left panel, siMAPL) versus siRNA control (right panel, siControl). Scale bar 10 µm.Western blot analysis of total cell lysates from U2OS BAX/BAK DKO cells after siRNA‐mediated depletion of MAPL (siMAPL) versus siRNA control (siControl) probed against MAPL. Whole cell protein staining (Ponceau S) is shown to confirm equal loading. Data are representative of *n* = 3 independent experiments.Quantification of BAX/DRP1 interaction after siRNA‐mediated MAPL knock‐down (siMAPL) versus knock‐down control (siControl) in healthy or apoptotic conditions (+/− STS). Box plots represent the interquartile (box), median (line) and SD (whiskers) of *n* = 3 independent experiments. Significance was tested by paired two‐tailed Student's *t*‐test (n.s. indicating *P* > 0.05). Source data are available online for this figure.

When we evaluated the expression, localization, and activity of the noninteracting BAX mutants Δ19–37, ΔL1‐2, and L63E (Fig EV4A–C), we found that they all could be expressed, although they seemed aggregated (BAX(Δ19–37)) or appeared constitutively mitochondrial concentrated at discrete foci (BAX(L63E) and BAX(ΔL1–2)). While they all retained their ability to induce apoptosis, BAX mutants Δ19–37 and ΔL1–2 presented an increased tendency to auto‐activate, likely explaining their lower levels detected by Western Blot. In agreement with this, for these BAX mutants, mitochondria appeared stressed under resting conditions.

Following a similar rationale, we investigated whether DRP1 mutants affected in different aspects of its activity exhibited altered interaction with BAX. We tested the K38A mutation, which abolished the GTPase activity (Figueroa‐Romero *et al*, 2009), the D221A mutation interfering with high order oligomerization (Kalia *et al*, 2018), the R376E mutation that blocks binding to MFF (Strack & Cribbs, 2012), the K642E mutation that renders DRP1 mostly monomeric *in vitro* and cytosolic in healthy cells (Frohlich *et al*, 2013) and the combination of K557/560/569/571R mutations that affect DRP1 SUMOylation (Figueroa‐Romero *et al*, 2009) (Fig 5F). As shown in Fig 5G and H, none of these mutants clearly abolished the ddFP signal with BAX in apoptosis. Of note, unlike for wild type DRP1 and the K38A mutation, which produced ddFP signal at early stages of apoptosis induction, the ddFP signal of the rest of the mutants was observed in combination with highly damaged mitochondria typical of advanced stages of apoptosis induction. These results suggest that localization to mitochondria is the most important requirement for DRP1 interaction with BAX and that the catalytic activity is not required. However, from these experiments, we cannot discard that some of the mutants have indeed altered interaction at early time points of apoptosis.

In summary, these results suggest that the N‐terminal region of BAX comprising the segments corresponding to helices α1 and the loop between α1 and α2 are required for complex formation with DRP1 in cells and that α2 seems to be sufficient to promote their association. Interestingly, this DRP1‐interacting region is proximal to the rear site of BH3‐domain binding in BAX, on the opposite side of the canonical hydrophobic groove involved in BID BH3 binding (Fig EV4D). In contrast, deletion of the C‐terminal helix α9 or helices α4 and α5 is not sufficient to disrupt binding to DRP1, although these segments of BAX may still participate in the interaction surfaces.

### Forced interaction between DRP1 and BAX induces their accumulation at apoptotic foci in mitochondria, mitochondrial remodeling, and MOMP

**Figure 6:**
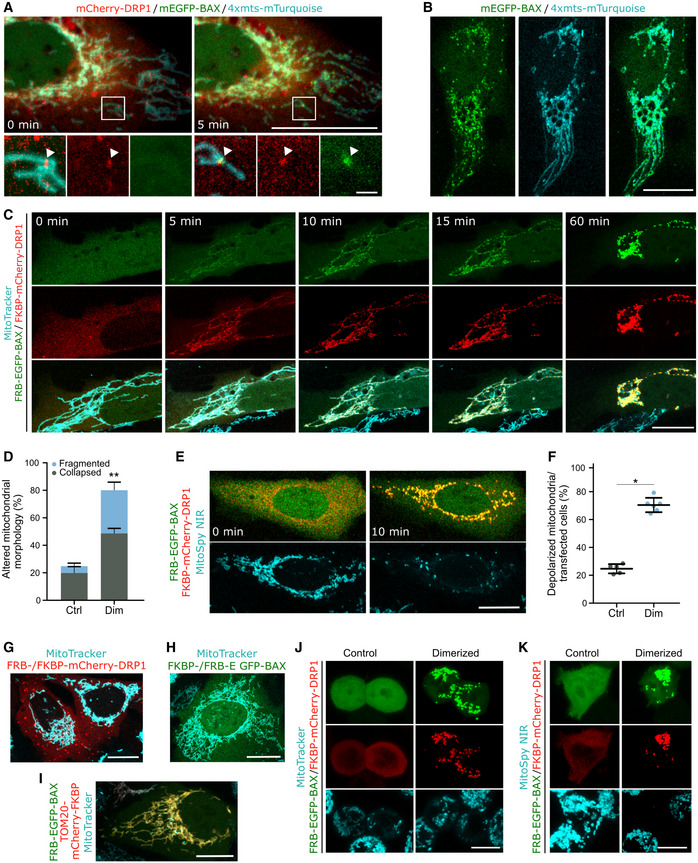
Induced dimerization of BAX and DRP1 induces MOMP AHierarchy of BAX and DRP1 recruitment to mitochondria in apoptotic cells. Confocal microscopy image of U2OS BAX/BAK DKO cells transfected with mCherry‐DRP1 (red), mEGFP‐BAX (green) and 4xmts‐mTurquoise (cyan) to label mitochondria. After apoptosis induction, translocation of DRP1 to mitochondria and foci formation (set to 0 min) was observed before BAX foci formation (5 min later). Zoomed images correspond to crop regions as indicated. Arrowheads highlight co‐localizing foci of DRP1 and BAX. Scale bar 20 µm, zoomed images 2 µm.BRepresentative confocal microscopy image of BAX foci formation in apoptotic U2OS BAX/BAK DKO cells transfected with mEGFP‐BAX (green) and 4xmts‐mTurquoise (cyan) after siRNA‐mediated DRP1 depletion. Scale bar 20 µm.CForced dimerization of BAX and DRP1 in healthy cells induces their translocation to mitochondria and foci formation. Confocal microscopy images of U2OS BAX/BAK DKO cells transfected with FKBP‐mCherry‐DRP1 (red) and FRB‐EGFP‐BAX (green) and stained with MitoTracker Deep Red FM (cyan) were acquired before (0 min) and after induction of BAX/DRP1 dimerization (5–60 min). From top to down, the individual rows correspond to fluorescence signal of BAX and DRP1 and the merge of both together with the MitoTracker signal, respectively. Scale bar 20 µm.DQuantification of mitochondrial morphology normalized to the number of transfected cells before (Ctrl) and after induced dimerization of BAX and DRP1 (Dim). Significance was tested using paired two‐tailed Student's *t*‐test (**P < 0.01) compared to untreated control.EInduced dimerization of BAX and DRP1 causes mitochondrial depolarization. U2OS BAX/BAK DKO cells were transfected as described in (C) and stained with the mitochondrial membrane potential‐sensitive dye MitoSpy NIR (cyan). Confocal microscopy images were acquired before (0 min) and after induced dimerization of BAX and DRP1 (10 min). Scale Bar 20 µm.FQuantification of mitochondrial depolarization normalized to the number of transfected cells before (Ctrl) and after induced dimerization of BAX and DRP1 (Dim). Significance was tested using paired two‐tailed Student's *t*‐test (*P < 0.05) compared to non‐induced control.G–KInduced dimerization of DRP1 with itself (G), BAX with itself (H), BAX with TOM20 (I) in U2OS BAX/BAK DKO cells, or BAX with DRP1 in HCT OctaKO cells (J, K), transected with FKBP‐ and FRB‐mCherry DRP1 (G, red), FKBP‐ and FRB‐EGFP‐BAX (H, green), TOM20‐mCherry‐FKBP (red) and FRB‐EGFP‐BAX (green, I), respectively. Mitochondria were stained using MitoTracker Deep Red FM or MitoSpy NIR (cyan) as indicated. Scale bar 20 µm (G‐I) and 10 µm (J, K). All images are representative of *n* = 3 independent experiments. Data information: Values in (D and F) are presented as mean (bar, line) ± SD of *n* = 3 independent biological experiments (with *n* = 100 cells each). All images are representative of *n* = 3 independent experiments.

The association between BAX and DRP1 at apoptotic foci raised the question whether any of these proteins recruited the other to these sites during apoptosis. To test this possibility, we visualized the kinetics of the accumulation of mEGFP‐BAX and mCherry‐DRP1 at mitochondria of U2OS BAX/BAK DKO cells following apoptosis induction with BCL‐2 and MCL‐1 inhibitors ABT‐737 and S63845. The mitochondria were labeled with the fluorescent protein mTurquoise2 targeted to mitochondria (4xmts‐mTurquoise). Despite the technical difficulties to detect the proteins in the initial stages of apoptotic foci formation because of the low contrast due to high cytosolic background, mCherry‐DRP1 foci repeatedly appeared at mitochondria prior to mEGFP‐BAX in all cases, we could observe (Fig 6A). However, GFP‐BAX also accumulated at discrete apoptotic foci in apoptotic cells depleted of DRP1 (Fig 6B). Together, these observations suggest that DRP1 is recruited to apoptotic mitochondrial foci upstream of BAX and may contribute to BAX translocation; it is not required for this process. Furthermore, knock‐down of MAPL to block DRP1 SUMOylation did not disrupt the RA‐BAX/GB‐DRP1 complex formation in ddFP experiments, indicating that this posttranslational modification of DRP1 in apoptosis is not required for BAX/DRP1 interaction at apoptotic foci and may take place downstream (Fig EV4E–G). Altogether, these results suggest that BAX and DRP1 do not actively recruit each other to the apoptotic mitochondrial foci, but that they form a complex in their mitochondria‐bound form.

**Figure EV5:**
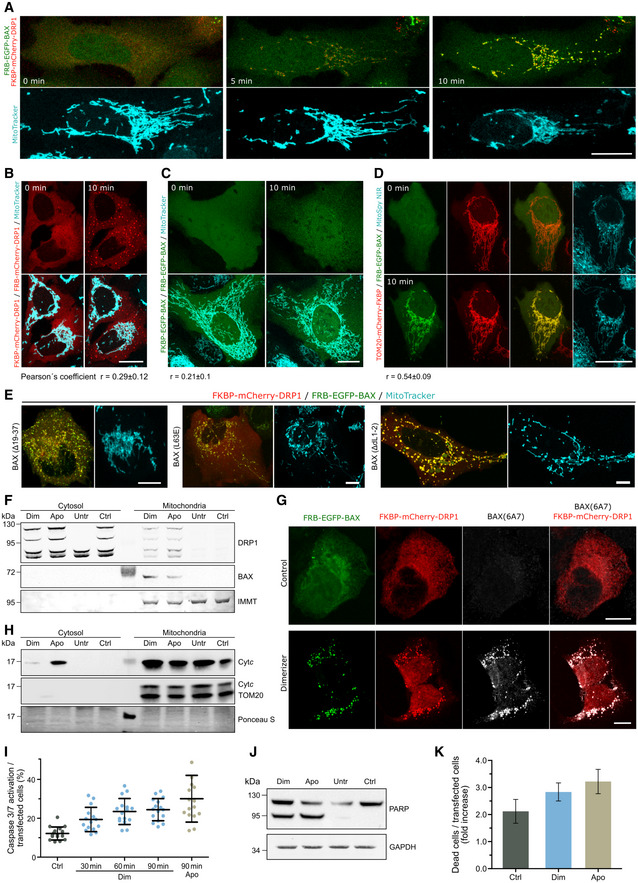
Induced dimerization of BAX and DRP1 induces apoptosis AConfocal microscopy of U2OS BAX/BAK DKO cells transfected with FKBP‐mCherry‐DRP1 (red) and FRB‐EGFP‐BAX (green) and stained with MitoTracker Deep Red FM (cyan). Images were acquired before (0 min) and after induction of BAX/DRP1 dimerization (5, 10 min). Scale bar 20 µm.B–DInduced dimerization of DRP1 to itself (B), BAX to itself (C) or BAX to TOM20 (D) in U2OS BAX/BAK DKO cells transected with FKBP‐ and FRB‐mCherry DRP1 (B, red), FKBP‐ and FRB‐EGFP‐BAX (C, green) or TOM20‐mCherry‐FKBP (red) and FRB‐EGFP‐BAX (green, D) before (0 min) and after induced dimerization (10 min). Mitochondria were stained using MitoTracker Deep Red FM or MitoSpy NIR (cyan) as indicated. Scale bar 20 µm. Pearson's correlation coefficient was calculated between the induced dimerization signal of BAX/BAX, DRP1/DRP1 and BAX/TOM20, respectively, and mitochondria based on MitoTracker Deep Red TM signal as depicted below the images.EInduced dimerization of DRP1 and BAX mutants L63E, Δ19–37 or ΔL1–2. Confocal microscopy of U2OS BAX/BAK DKO cells transfected with FKBP‐mCherry‐DRP1 (red) and mutant variants FRB‐EGFP‐BAX (green) as indicated and stained with MitoTracker Deep Red FM (cyan). Images were acquired 10 min after induction of BAX/DRP1 dimerization. Scale bar 20 µm. All images are representative of *n* = 3 independent experiments.F–HDimerization of BAX and DRP1 induces their translocation to mitochondria, exposure of the BAX 6A7 epitope and cytochrome *c* release. (F) Western blot analysis of cytosolic and mitochondrial fraction from U2OS BAX/BAK DKO overexpressing FKBP‐mCherry‐DRP1 and FRB‐EGFP‐BAX after induction of BAX/DRP1 dimerization (Dim) or apoptosis (Apo) compared to non‐treated (Ctrl) or untransfected cells (Untr). Western blot was probed against DRP1 and BAX. Mitofilin (IMMT) was used to test for purity of cytosolic fraction. Results are representative for *n* = 3 independent experiments. (G) Representative (*n* = 3 independent experiments) confocal microscopy images of U2OS BAX/BAK DKO cells transfected with FKBP‐mCherry‐DRP1 (red) and FRB‐EGFP‐BAX (green). Cells were immunostained against the 6A7 epitope of BAX (grey) after induced dimerization of BAX and DRP1 (Dimerizer) compared to untreated (Control). Images show individual channels or overlay of FKBP‐mCherry‐DRP1 and BAX‐(6A7) immunofluorescence signal. Scale bar 10 µm. (H) Representative (*n* = 3 independent experiments) western blot analysis of the cytosolic and mitochondrial fraction from U2OS BAX/BAK DKO treated as described in F) and probed against cytochrome *c* (Cyt*c*). TOM20 was used to test for purity of cytosolic fraction. Protein staining (Ponceau S) is shown to confirm equal loading.I–KQuantification of caspase activation, PARP cleavage and cell death induced by forced dimerization of BAX and DRP1. (I) U2OS BAX/BAK DKO cells were transfected with FKBP‐mCherry‐DRP1 and FRB‐EGFP‐BAX and analyzed for the percentage of cells with caspase 3/7 activation. Data are normalized to the number of transfected cells at individual time points after inducing dimerization of BAX and DRP1 (Dim, 30, 60 and 90 min) compared to apoptosis induction for 90 min (Apo, 90 min) or untreated cells (Ctrl). (J) Representative western blot analysis (*n* = 3 independent experiments) of total cell lysates from cells transfected as described in (I) after induction of BAX/DRP1 dimerization (Dim) or apoptosis (Apo) compared to non‐treated (Ctrl) or untransfected cells (Untr). Blot was probed against PARP or GAPDH as a loading control. K) U2OS BAX/BAK DKO cells were transfected as described in (I) and induced for BAX/DRP1 dimerization (Dim) or apoptosis (Apo) compared to untreated (Ctrl). Data are presented as fold increase of dead cells 1 h after treatment compared to the number of dead cells before treatment and normalized to the number of transfected cells. Data information: Values in (I) and (K) are representative of *n* = 2 independent experiments and correspond to mean (bar, line) ± SD. Single data points in (I) represent individual measurements from technical and biological replicates. Source data are available online for this figure.

Our findings indicate that interaction between BAX and DRP1 affects the membrane activities of both proteins (BAX pore formation and membrane permeabilization, and DRP1 membrane tethering activity). However, so far it has not been possible to examine the functional relevance of this interaction during apoptosis in cells due to the crucial role of DRP1 in maintaining mitochondrial dynamics and homeostasis. Alteration of DRP1 levels by depletion or overexpression modifies mitochondrial structure and function, which then indirectly affects the sensitivity of cells to apoptosis. Unfortunately, the alteration of the apoptotic activity in the noninteracting BAX mutants in Fig 5 also precluded their use to address this question (Fig EV4A–C). To overcome these limitations, we implemented a cellular inducible protein dimerization system (iDimerize™ Inducible Heterodimer System, Takara Bio). In this system, the addition of an external dimerizer (A/C Heterodimerizer) to the cells induces the artificial dimerization of two protein domains, FK506‐binding protein (FKBP) and FKBP rapamycin‐binding domain of mTOR (FRB), and subsequently of the proteins they are tagged to. We expressed FRB‐EGFP‐BAX and FKBP‐mCherry‐DRP1 in U2OS BAX/BAK DKO cells and added A/C heterodimerizer to induce BAX/DRP1 dimerization. Our live cell imaging experiments strikingly showed that FRB‐EGFP‐BAX and FKBP‐mCherry‐DRP1 rapidly translocated to mitochondria after the addition of the dimerizer, where both proteins accumulated together at discrete sites that resembled apoptotic foci (Fig 6C). Furthermore, FRB‐EGFP‐BAX/FKBP‐mCherry‐DRP1 translocation was accompanied by a reorganization of the mitochondrial network, which appeared to fragment and collapse at peri‐nuclear regions, and by MOMP, measured as loss of mitochondrial potential (Figs 6D–F and EV5A).

As negative controls, we performed the same experiments in cells expressing FRB‐ and FKBP‐tagged with only DRP1 or BAX and quantified their mitochondrial localization by colocalization analysis with the Pearson’s coefficient (Fig EV5B–D). Addition of dimerizer to cells expressing FRB‐ and FKBP‐tagged DRP1 led to the formation of DRP1 dots that did not colocalize with mitochondria (Figs 6G and EV5B). When dimerizer was added to cells expressing FRB‐ and FKBP‐tagged BAX, the protein maintained its cytosolic distribution and neither MOMP nor apoptosis were induced (Figs 6H and EV5C). Of note, this is in contrast to the induced dimerization activation of BAX reported by Gross and colleagues (Gross *et al*, 1998), which could be due to the inclusion of a fluorescent protein as additional spacer between BAX and the FRB/FKBP domains in our constructs. To rule out the possibility that DRP1 activates BAX unspecifically by simply inducing its accumulation in mitochondria, we used an alternative way of targeting BAX to mitochondria by inducing the binding of FRB‐EGFP‐BAX and FKBP‐tagged TOM20 with the dimerizer. As shown in Figs 6I and EV5D, despite BAX relocalization to mitochondria, this combination also failed to induce BAX distribution into foci and MOMP, suggesting that the interaction with DRP1 specifically promotes BAX activation. In addition, when we induced the dimerization of DRP1 with the noninteracting BAX mutants Δ19–37, ΔL1–2, and L63E in living cells, we detected the recruitment of DRP1 to where BAX was initially localized thanks to the FRB/FKBP domains. However, in this case, their association did not lead to major cellular alterations beyond those already induced by the expression of the BAX mutants without dimerizer, compared to the wild type (Fig EV5E). For BAXΔL1–2, the foci‐like distribution was increased in those cells that did not clearly have it before dimerization. Remarkably, chemically induced dimerization of FRB‐EGFP‐BAX and FKBP‐mCherry‐DRP1 in HCT116 OctaKO cells, which are depleted of BH3‐only proteins, also led to their accumulation into mitochondrial foci, mitochondrial reorganization and depolarization (Fig 6J and K).

Finally, we established that the mitochondrial alterations promoted by the induction of BAX/DRP1 interaction were accompanied by the hallmarks of apoptosis induction (Figs 6F and EV5F–K). We confirmed that dimerizer treatment promoted FRB‐EGFP‐BAX and FKBP‐mCherry‐DRP1 accumulation in mitochondria by Western Blot. This was accompanied by BAX activation and exposure of the 6A7 epitope, loss of mitochondrial potential, cyt *c* release into the cytosol, caspase activation, PARP cleavage, and cell death.

Altogether, these findings indicate that although DRP1 and BAX are not required for each other’s recruitment to apoptotic foci, their interaction promotes their accumulation at discrete sites in mitochondria as well as BAX activation independently of BH3‐only proteins, which results in the induction of apoptosis.

## Discussion

While a connection between BAX and DRP1 was proposed more than a decade ago based on the colocalization that both proteins exhibit during apoptosis, the underlying molecular basis and its relevance in apoptosis have remained rather obscure and a matter of debate (Karbowski *et al*, 2002; Parone *et al*, 2006; Estaquier & Arnoult, 2007; Sheridan *et al*, 2008). A thorough study previously failed to detect physical contacts between the two proteins and instead proposed that the effect of DRP1 on BAX apoptotic activity would be solely mediated by the membrane remodeling induced by DRP1, without interaction (Montessuit *et al*, 2010). Here, we used a combination of multiple orthogonal approaches both *in vitro* and in cells to challenge this notion and demonstrate the physical interaction between BAX and DRP1 under several experimental conditions.

First, BAX and DRP1 colocalize at mitochondrial apoptotic foci in SMLM images with a spatial resolution of around 30 nm. Given that both proteins are known to form supra‐molecular larger structures (Frohlich *et al*, 2013; Große *et al*, 2016; Salvador‐Gallego *et al*, 2016), our results are a strong indicator of molecular contact between them. Second, we resolve the spatiotemporal dynamics of the interaction between BAX and DRP1 in apoptosis by ddFP, which reveals that the two proteins form part of the same complex (< 10 nm) (Ding *et al*, 2015) in tight correlation with the loss of mitochondrial potential upon MOMP and that they stay together until the death of the cell. Although these results pinpoint the association of BAX and DRP1 in the physiological environment of the cell and specifically in the context of apoptosis induction, they cannot discern whether BAX and DRP1 binding is direct or mediated by other cellular components. This issue is solved by a third approach in which the detection of codiffusion of BAX and DRP1 by FCCS in reconstituted systems reveals that the two proteins only associate when bound to the membrane. It also shows that the affinity between them is high, as we detect significant complex formation at the low membrane densities used in FCCS, and that the interaction can be regulated, based on the competition observed by cBID. Our findings with the peptide array in GUVs and with cross‐linking coupled to mass spectrometry in LUVs provide additional evidence for the direct binding between both proteins *in vitro*. Thus, our results clearly establish a direct interaction between BAX and DRP1, which may have escaped previous characterization due to the technical difficulties linked to the necessity of a lipid membrane for the interaction.

The requirement of the membrane for the interaction between BAX and DRP1 has important implications. Both proteins present distinct conformations and oligomeric states when they are soluble in the cytosol or embedded in the mitochondrial outer membrane. Our findings thereby strongly suggest that it is specifically the membrane‐bound conformation of these proteins that fulfills the structural requirements for association. Although the conformation of DRP1 assemblies on constricted lipid tubes has been solved (Frohlich *et al*, 2013), we only have incomplete or low‐resolution information about the structure of BAX in the membrane (Czabotar *et al*, 2013; Salvador‐Gallego *et al*, 2016; Hauseman *et al*, 2020; Lv *et al*, 2021). It will be very interesting to uncover the structural details of the association between the macromolecular structures formed by both proteins in membranes.

We also show how the binding between BAX and DRP1 affects their biochemical activities on membrane model systems. The enhancement of pore formation by BAX is in line with a pro‐apoptotic role of DRP1. Although our data are not at odds with an additional indirect effect of the membrane remodeling activities exerted by both proteins (Montessuit *et al*, 2010), the implication of the pore‐forming regions and the C‐terminal transmembrane anchor of BAX in the interaction with DRP1 suggest that DRP1 could affect BAX permeabilizing activity by directly tuning the BAX pore (Garcia‐Saez *et al*, 2005; Zhang *et al*, 2016). On the other hand, the effect of BAX on the membrane tethering activity of DRP1 suggests a role on the extensive mitochondrial fragmentation mediated by DRP1 during apoptosis, which would not be at the level of its GTPase activity. We propose that the participation of these membrane‐interacting regions of BAX in contact with DRP1 may impact the membrane fission process mediated by DRP1.

In cells, we find that forcing the interaction between BAX and DRP1 shifts the equilibrium toward their membrane‐bound active conformations. Two main aspects underlie the difficulties so far in defining the functional role of the association between BAX and DRP1 by genetic ablation. First, the impossibility of genetic approaches to disentangle a direct effect of DRP1 on BAX activity *versus* an indirect effect on mitochondrial structure and function that ultimately impacts BAX activity and apoptosis. Second, the functional overlap of the multiple regulatory functions by BCL‐2 family members, special the BH3‐only proteins, which provide the cell with multiple mechanisms for BAX activation that can replace for each other. We now solve this conundrum by using artificial dimerizers, which provide temporal control of the interaction and a direct readout of the specific consequences of bringing BAX and DRP1 together, which lead to BAX activation and apoptosis even in the absence of other BH3‐only proteins. Our findings thus establish an activating role for their interaction and open up new and intriguing scenarios in which DRP1, which lacks a BH3 domain, could replace and or modulate the canonical BH3‐mediated activation of BAX.

**Figure EV6:**
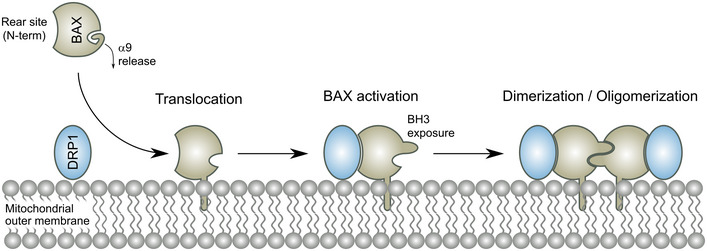
Model for the interaction between BAX and DRP1 in apoptosis During apoptosis, DPR1 binds to mitochondrial BAX via multiple interfaces, with the N‐terminus of BAX being required for the interaction. The interaction between BAX and DRP1 promotes BAX activation and apoptosis induction.

In this regard, our results that the N‐terminal region of BAX restricts its activation and is also required for the interaction with DRP1 suggest a mechanism how DRP1 could promote BAX activation. Previous studies have shown that, besides the canonical hydrophobic groove of BAX involved in regulatory interactions with BH3 domains, binding to a rear site in soluble BAX can trigger its activation (Gavathiotis *et al*, 2008). Furthermore, Kluck and colleagues elegantly dissected the role of the N‐terminus of BAX and BAK activation and reported that antibodies binding to the α1‐α2 loop are capable of activating mitochondria‐associated BAX (Westphal *et al*, 2014; Alsop *et al*, 2015; Iyer *et al*, 2016). Taking this into consideration, our data support a new mechanism by which DRP1 would activate membrane‐bound BAX by binding to its N‐terminal region and triggering the conformational changes leading to BAX activation and oligomerization (Fig EV6). To our best knowledge, DRP1 would represent the first cellular component using this site in membrane‐bound inactive BAX to promote its activation.

In summary, here we report that BAX and DRP1 directly interact specifically during apoptosis and that their association is spatially restricted to apoptotic foci on mitochondria. We identify the domains in BAX involved in the association between these two proteins, which include the pore‐forming hairpin and the transmembrane anchor, and require the N‐terminal region. Our results establish the pro‐death function resulting from the interplay between BAX and DRP1, which promotes the activation of BAX via a non‐BH3 mechanism, leading to MOMP and apoptotic cell death. Together, our findings support a new model for how the physical contact with DRP1 promotes both the activation and pore activity of BAX, thus defining the molecular and functional interplay between these two proteins.

## Materials and Methods

### Plasmids and antibodies

The plasmids and antibodies used in this study are listed in Tables 1 and 2, respectively.

**Table 1:** Plasmids.

Name (short name)	Resistance	Promoter	Description
pEGFP‐C1‐BAX (GFP‐BAX)	Kan	CMV	For expression of EGFP‐tagged BAX (or mutant versions of BAX) in mammalian cells.
pAcGFP‐C1‐GB‐BAX (GB‐BAX)	Kan	CMV	For mammalian expression of GB‐tagged BAX.
pAcGFP‐C1‐RA‐BAX (RA‐BAX)	Kan	CMV	For mammalian expression of RA‐tagged BAX (or mutant versions of BAX as indicated).
pAcGFP‐C1‐GB‐DRP1 (GB‐DRP1)	Kan	CMV	For mammalian expression of GB‐tagged DRP1 (or mutant versions of DRP1 as indicated).
pAcGFP‐C1‐mitoBFP (mitoBFP)	Kan	CMV	For expression of mitochondria‐localized BFP in mammalian cells. Provided by Dr. Gia Voeltz lab.
pAcGFP‐C1‐RA‐BCLxL (RA‐BCL‐xL)	Kan	CMV	For mammalian expression of RA‐tagged BCL‐xL.
pSmac‐GFP	Kan	CMV	For expression of GFP‐tagged Smac in mammalian cells. Addgene no. #40881
pAcGFP‐C1‐mCherry‐DRP1 (mCherry‐DRP1)	Kan	CMV	For mammalian expression of mCherry‐tagged DRP1 isoform 3 (or mutant versions of DRP1 as indicated).
pEGFP‐A206K_BAX_C1 (mEGFP‐BAX)	Kan	CMV	For expression of monomeric EGFP(A206K)‐tagged BAX in mammalian cells.
pEGFP‐N1‐4xmt‐mTurquoise2 (4xmts‐mTurquoise)	Kan	CMV	For visualization of mitochondria with mTurquoise2 in mammalian cells.
pFKBP‐EGFP‐BAX	Kan	CMV	For mammalian expression of EGFP‐tagged BAX fused to the FKBP inducible dimerization domain.
pFRB‐EGFP‐BAX	Kan	CMV	For mammalian expression of EGFP‐tagged BAX (or mutant versions of BAX) fused to the FRB inducible dimerization domain.
pFKBP‐mCherry‐DRP1	Kan	CMV	For mammalian expression of mCherry‐tagged DRP1 fused to the FKBP inducible dimerization domain.
pFKBP‐EGFP‐DRP1	Kan	CMV	For mammalian expression of GFP‐tagged DRP1 fused to the FKBP inducible dimerization domain.
pFRB‐mCherry‐DRP1	Kan	CMV	For mammalian expression of mCherry‐tagged DRP1 fused to the FRB inducible dimerization domain.
pAcGFP‐TOM20‐mCherry‐FKBP (TOM20‐mCherry‐FKBP)	Kan	CMV	For mammalian expression of mCherry‐tagged TOM20 fused to the FKBP inducible dimerization domain.

**Table 2:** Antibodies.

Antibody	Dilution	Source	Application
α‐BAX (6A7)	1:100	Thermo Fischer (MA5‐14003)	IF
α‐BAX	1:1,000	CST (#2772)	WB
α‐Cyt*c*	1:1,000	BD (556433)	WB
α‐DRP1	1:30	CST (#26954)	IF
α‐DRP1 (D6C7)	1:1,000	CST (#8570)	WB
α‐GAPDH (D4C6R)	1:1,000	CST(#97166)	WB
α‐GFP	1:1,000	Roche (11814460001)	WB
α‐GFP nanobody AF647	1:2,000	Chromotek (home labeled)	IF
α‐IMMT (Mitofilin)	1:1,000	10179‐AP	WB
α‐MAPL (MUL1)	1:1,000	Sigma (SAB2702071)	WB
α‐mouse CF680	1:500	Sigma (SAB4600371)	IF
α‐mouse AF647	1:500	Thermo Fischer (A28181)	IF
α‐mouse IgG‐HRP	1:10,000	JIR (115‐035‐003)	WB
α‐mouse IgG‐IRDye 800CW	1:10,000	Li‐Cor (926‐32212)	WB
α‐PARP	1:1,000	CST (#9542)	WB
α‐rabbit IgG‐HRP	1:10,000	JIR (111‐035‐003)	WB
α‐rabbit IgG‐IRDye 680LT	1:20,000	Li‐Cor (926‐68023)	WB
α‐Tom20 (D8T4N)	1:1,000	CST #42406	WB

IF, Application Immunofluorescence; WB, Western Blot.

### Cell culture

Human osteosarcoma U2OS double *BAX* ^−/−^ *BAK* ^−/−^ (BAX/BAK DKO), MEF *DRP1* ^−/−^ (DRP1 KO) and HeLa cells were cultivated in DMEM, HCT116 *BAD* ^−/−^ *BID* ^−/−^ *BIK* ^−/−^ *BIM* ^−/−^ *BMF* ^−/−^ *HRK* ^−/−^ *NOXA* ^−/−^ *PUMA* ^−/−^ (HCT OctaKO) cells in McCoy 5A medium supplemented with 10% (v/v) FBS and 1% (v/v) penicillin/streptomycin (Invitrogen, Germany) at 37°C and 5% (v/v) CO_2_. Cells were transfected one day after seeding at 70–80% confluence using Lipofectamine™ (Invitrogen, Germany) and Opti‐MEM™ reduced serum medium (Gibco). The HeLa cell line used in this study was authenticated confirming 100% match to the DNA profile of HeLa (ATCC^®^ CCL‐2™) and 100% match over all 15 autosomal short tandem repeats to the DNA profile of HeLa (Cellosaurus, RRID:CVCL_0030 [PubMed=25877200]). HeLa and U2OS BAX/BAK DKO cell lines were tested mycoplasma negative.

### siRNA‐mediated knock‐down

The expression of the MAPL or DRP1 was downregulated using siRNA‐mediated knock‐down. Cells were cultivated in 6‐well plates to 70–80% confluence and transfected with 5 nM siRNA against MAPL (siMUL1, L‐007062‐00‐0005), DRP1 (5′‐GGAGCCAGCTAGATATTAA‐3′) or a control scramble siRNA (D‐001810‐01‐05, all siRNAs were purchased from Dharmacon™) in 1 ml Opti‐MEM reduced serum medium (Gibco) using 1 µl of Lipofectamine 2000 (Invitrogen, Germany). After 6 h, 1 ml of DMEM supplemented with 20% (v/v) FBS and 1% (v/v) penicillin/streptomycin (Invitrogen, Germany) was added. siRNA‐mediated knock‐down was performed 4 days before microscopy experiments. Efficient protein downregulation was verified by western blot (antibodies see Table 2).

### Western blot

Cells were harvested with Trypsin‐EDTA (Sigma), resuspended in culture medium, collected by centrifugation at 300–400 *g* at 4°C for 5 min, and washed twice with ice‐cold PBS. For lysis, cell pellets were resuspended in RIPA lysis buffer (50 mM of Tris/HCl pH 8.0, 150 mM NaCl, 1% (v/v) Triton™ X‐100, 0.5% (w/v) sodium deoxycholate, 0.1% (w/v) SDS), incubated on ice for 20 min and spun at 20,000 *g* at 4°C for 20 min to remove cellular debris.

Protein concentration was determined by Bradford protein assay (Bio‐Rad) according to the manufacturer’s protocol. A total amount of 50–100 µg protein was boiled in SDS‐PAGE sample buffer (62.5 mM of Tris/HCl pH 6.8, 2% (w/v) SDS, 10% (v/v) glycerol, 0.005% (v/v) ß‐Mercaptoethanol, 0.01% (w/v) bromophenol blue) for 5 min at 95°C prior to SDS‐PAGE. Proteins were transferred to nitrocellulose membrane (Trans‐Blot Turbo, BioRad) and equal sample loading was tested using Ponceau S staining. Blots were washed with TBST (50 mM of Tris/HCl pH 7.5, 150 mM of NaCl, 0.1% (v/v) Tween 20) and blocked with 5% (w/v) low‐fat milk in TBST for 60 min. Primary antibodies (Table 2) were diluted in 5% (w/v) milk in TBST and incubated for 16 h at 4°C. After 3x washing with TBST for 5 min, HRP‐coupled or fluorescent secondary antibodies (Table 2) were incubated for 1 h at room temperature. Blots were washed 3x with TBST and detected with SuperSignal™ West Pico PLUS chemiluminescenct substrate (Thermo Scientific) using the Fusion SL Gel Chemiluminescence Documentation System (Vilber Lourmat) or scanned using an Odyssey CLx imaging system (Li‐Cor Biosciences) when fluorescent antibodies were used. Images were adjusted in brightness and contrast and cropped using Fiji/ImageJ (Schindelin *et al*, 2012).

### Single‐molecule localization microscopy

HeLa cells were grown on 24 mm round glass coverslips for 24 h and transfected with 200 ng pEGFP‐C1‐BAX (Table 1) for 12 h. Apoptosis was induced with 1 µM STS (Sigma) for 3 h and then cells were fixed in 4% (v/v) PFA in PBS for 15 min at room temperature. Coverslips were incubated for 15 min in PBS with 50 mM of NH_4_Cl and permeabilized in PBS with 0.25% (v/v) Triton™ X‐100 for 5 min. Cells were washed 3x for 5 min in PBS and blocked for 45 min in PBS with 1% (w/v) BSA. Labeling was done with AF647–anti‐GFP nanobodies and/or anti‐DRP1 primary antibodies (Table 2) in 1% (w/v) BSA for 90 min. After extensive washes with PBS, coverslips were incubated for 30 min at room temperature with CF680‐ or AF647‐coupled secondary antibodies (Table 2). Samples were mounted and imaged in a custom‐made microscope (Deschamps *et al*, 2016) and covered with 300 µl of imaging buffer (50 mM of Tris‐HCl pH 8, 10 mM of NaCl, 10% (w/v) glucose, 35 mM of cysteamine (MEA), 0.5 mg/ml of glucose oxidase (Sigma) and 40 μg/ml of catalase (Sigma)). An exposure time of 15 ms for single color and 30 s for dual color measurements was used with an EM gain of 100. Imaging laser intensity at 640 nm was 2.5 kW/cm^2^ and the 405 nm activation laser intensity was automatically adjusted to keep a constant number of localizations per frame. Typically, 70,000–100,000 frames were recorded. Analysis was performed using SMAP (Ries, 2020). Localizations with uncertainties above 15 nm were discarded. Images were rendered using a Gaussian with a width according to the localization precision. Image analysis was done with Fiji/ImageJ (Schindelin *et al*, 2012). Quantification of the distance between BAX and DRP1 was done by analyzing all colocalizing BAX and DRP1 structures (maximal distance 180 nm) and measuring the distance from the center of every BAX structure to the center of the DRP1 structures. Quantification was done from *n* = 4 independent experiments (with a total of 720 BAX structures) and performed in a blinded fashion to exclude confirmation bias.

### Confocal imaging

Confocal microscopy was performed using an LSM710 confocal microscope with a C‐Apochromat 40X/1.2 water immersion objective (Zeiss, Jena, Germany) with laser lines to excite at 405, 488, 561, or 633 nm. Emitted fluorescence was separated using a spectral beam guide and detected on photomultiplier tube (PMT) or avalanche photodiode (APD) detectors. Confocal imaging for induced dimerization experiments was performed on a TCS SP8 (Leica Microsystems) inverse confocal laser scanning microscope equipped with a PL Apo 63×/1.40 Oil CS2 objective and a tunable white light laser (470–670 nm). Fluorescence emission was detected using HyD SMD detectors. Live cell imaging was performed under 5% (v/v) CO_2_ and temperature control at 37°C. Images have been adjusted for brightness and contrast using Fiji/ImageJ (Schindelin *et al*, 2012).

### Dimerization‐dependent fluorescent protein (ddFP) experiments

Dimerization‐dependent fluorescent protein (ddFP) was described in Ding *et al* (2015). Cells were seeded in 8‐well chambered cover glass µ‐slides (IBIDI) and transfected with 50 ng pAcGFP‐C1‐RA‐BAX (or respective N‐terminal truncation, deletion or point mutation variants of BAX), 100 ng pAcGFP‐C1‐GB‐DRP1 (or mutation variants of DRP1), and 100 ng pAcGFP‐C1‐mitoBFP or 100 ng pSmac‐GFP (Table 1) to visualize the release of Smac from mitochondria. Apoptosis was induced using 1 µM of STS (Sigma) in DMEM without phenol red and cells were imaged after 3 h of apoptosis induction. To inhibit caspase activation or block the permeability transition pore cells were treated with 20 µM of Q‐VD‐OPh (Hölzel Biotech) for 3 h or 50 µM CsA (Sigma) for 30 min, respectively. Interaction of BAX and DRP1 was quantified from background‐corrected, maximum intensity z‐projection images by normalizing the number of RA‐BAX/GB‐DRP1 positive cells (cells that show RA‐BAX/GB‐DRP1 foci at mitochondria) to mito‐BFP positive cells. Interaction of BAX and DRP1 was additionally verified in apoptotic U2OS BAX/BAK DKO and MEF DRP1 KO cells transfected as described above and with apoptosis induction using 100 nM of Paclitaxel for 24 h or 10 μM of Etoposide for 6 h. Data analysis was performed form *n* = 3 independent experiments (with *n* = 100 cells each) in a blinded fashion to exclude confirmation bias. Release of Smac‐GFP from mitochondria during apoptosis was quantified by measuring the background‐corrected fluorescent intensity in a region of interest in the cytosol at different time points after apoptosis induction. Levels of significance were determined using Student’s *t*‐test.

### Protein production and labeling

Full‐length mouse BID, full‐length human BAX, and full‐length BCL‐xL were expressed in *E*. *coli* and purified as described in (Bleicken *et al*, 2013b, 2014; Ugarte‐Uribe *et al*, 2014). Cleaved BID (cBID) was obtained from BID as described in Bleicken *et al* (2014). Bovine cytochrome *c* (Cyt *c*) and allophycocyanine (APC) were purchased at Sigma‐Aldrich (Munich, Germany). Protein labeling was performed at single cysteine residues as described in Bleicken *et al* (2013b). Human DRP1 (isoform 1) was cloned into pTYB2 vector, expressed in *E. coli* at 14°C for 18 h and purified using chitin resin (New England Biolabs, Inc.). Labeling of DRP1 at amine groups was performed at pH 7.4 when DRP1 was still bound to the resin as in Ugarte‐Uribe *et al* (2014). The cleavage reaction proceeded at pH 8 overnight at 4°C with 30 mM of DTT, after which the protein was eluted from the column and dialyzed against 20 mM of HEPES/KOH pH 7.4, 500 mM of NaCl and 1 mM of MgCl_2_. In some cases, an additional step of anion exchanger column (Q Sepharose) was used for purity improvement. Purified protein was stored with 50% (v/v) glycerol.

### Composition of the lipid mixtures

The lipid mixture mimicking the MOM composition was prepared as in Bleicken *et al* (2016) with 49% egg l‐α‐phosphatidyl‐choline (PC), 27% egg l‐α phosphatidyl–ethanolamine (PE), 10% bovine liver l‐α‐phosphatidyl–inositol (PI), 10% 18:1 phosphatidyl–serine (PS) and 4% cardiolipin (CL) (all percentages represent molar ratio). In addition, a lipid composition with a high CL content was used in order to mimic the contact sites between the inner and the outer membranes of mitochondria (PC:CL:PE 54:26:20, molar ratio) (Montessuit *et al*, 2010). For the BAX peptide array, a lipid composition containing the biotinylated lipid 1,2‐distearoyl‐sn‐glycero‐3‐phosphoethanolamine‐N‐[biotinyl(polyethyleneglycol)‐2000] (DSPE‐PEG(2000) Biotin) was used (DOPC:DSPE‐PEG(2000) Biotin 8:2, molar ratio) to tether BAX peptides to the membrane. All lipids were purchased from Avanti polar lipids (Alabaster, AL, USA). The lipidic dye, DiD (1,1'‐Dioctadecyl‐3,3,3',3'‐Tetramethylindodicarbocyanine perchlorate), and the lipid, L‐α‐phosphatidylethanolamine‐N‐(lissamine rhodamine B sulfonyl) (Rhod‐PE), were used to visualize membranes in the confocal microscope and in the flow cytometer, respectively.

### LUV and GUV preparation

Large unilamellar vesicles (LUVs) for flow cytometry were prepared as described in Ugarte‐Uribe *et al* (2014). The lipid solution was passed 31 times through a membrane (200 nm pore size) using an extruder from Avestin (LiposoFast‐Basic, Avestin Inc., Mannheim, Germany). Giant unilamellar vesicles (GUVs) were produced by electroformation as described in Ugarte‐Uribe *et al* (2014).

### Fluorescence cross‐correlation spectroscopy

All experiments were performed at 22°C on an LSM710 microscope with a C‐Apochromat 40×/1.2 water immersion objective (Zeiss, Oberkochen, Germany). Excitation light came from Argon (488 nm) or HeNe lasers (633 nm). FCCS and two‐focus scanning FCCS measurements were performed using a Confocor 3 module as described in Bleicken *et al* (2017) and Ugarte‐Uribe *et al* (2018). Photon arrival times were recorded with a hardware correlator Flex 02‐01D/C (http://correlator.com; https://www.mathworks.com/matlabcentral/fileexchange/20784‐flex02‐01d‐correlator‐interface). For solution FCS measurements, the proteins of interest were mixed with GTPase buffer (20 mM of HEPES pH 7.4, 150 mM of KCl, 1 mM of MgCl_2_) with 0.1 mM of GTP in a total volume of 200 µl and incubated at least 30 min before measurements. Incubation and measurements were done in Nunc™ Lab‐Tek™ 8‐well chamber slides (Thermo Scientific) that were blocked with BSA before use. Solution FCCS analysis was carried out with Fluctuation Analyzer 4G software (http://fluctuations.de/downloads.html). For scanning FCCS on GUV membranes, recombinant proteins (20 nM of DRP1‐AF488, 50 nM of BAX‐AF633, and 200–300 nM of cBID) were mixed (nucleosides or M‐divi1 were added if needed) in GTPase buffer to a final volume of 300 µl and added to a blocked well in a Nunc™ Lab‐Tek™ 8‐well chamber slide (Thermo Scientific). GUVs were added to the protein solution and incubated for 10–15 min or heated at 42°C for 30 min (for BAX activation). All analyzed GUVs had a similar size of ~ 20 μm. Photons were collected during 300 s per measurement. The detection volume with two perpendicular lines across a GUV equator was repeatedly scanned. Data analysis was performed with home‐written software as in Garcia‐Saez *et al* (2009). The photon stream was binned in 2 µs and arranged it as a matrix such that every row corresponded to one‐line scan. Membrane movements were corrected by calculating the maximum of running average over several hundred line scans and shifting it to the same column. An average over all rows was fitted with a Gaussian and added only the elements of each row between −2.5 and 2.5σ to construct the intensity trace. Autocorrelation and spectral and spatial cross‐correlation curves were computed from the intensity traces and irregular curves resulting from instability and distortion were excluded. Auto‐ and cross‐correlation functions were fitted with a nonlinear least‐squares global fitting algorithm (Garcia‐Saez *et al*, 2009). In addition, values for the diffusion coefficients of BAX and DRP1 were obtained under the different experimental conditions used (Fig EV2A and B).

### LUV permeabilization assay

Experiments were performed as in Bleicken *et al* (2017). Briefly, LUVs were prepared by solving 2.5 mg dried lipid mixture in 500 µl buffer (20 nM of HEPES/KOH, pH 7.4, 150 mM of NaCl, and 80 mM of Calcein [fluorescein‐bis‐methyl‐iminodiacetic acid at pH 7.5]) using intensive vortexing paused by 4 cycles of freezing and thawing. The multilamellar vesicles were passed 31 times through an extruder (Avestin) using membranes with 400 nm of pore size (Avestin), followed by size exclusion column purification (Sephadex G‐50 beads, GE Healthcare). Calcein was entrapped in the vesicles at a self‐quenching concentration so that its release in external medium was accompanied by an increase of the intensity of fluorescence. 100 µM LUVs were incubated with proteins of interest (100 nM BAX, 100 nM DRP1, 10 nM cBID) in GTPase buffer (20 mM HEPES pH 7.4, 150 mM KCl, 1mM MgCl_2_) with 1 mM GTP, at room temperature (in the presence of cBID) or at 42°C (in the absence of cBID in order to activate BAX by heat). The kinetics of calcein release were measured using a Tecan Infinite M200 microplate reader (Tecan, Männedorf, Switzerland). The percentage of release was calculated as described in (Bleicken *et al*, 2017). The experiment was performed three times in duplicate and the results are presented as mean ± SD.

### GUV permeabilization assay

The experiments were performed as described in Bleicken *et al* (2013a). Cyt *c*_488_, APC, and the proteins of interest (20 nM BAX, 100 nM DRP1, 20 nM cBID) were mixed in Nunc™ Lab‐Tek™ 8‐well chamber slides (Thermo Scientific) with GTPase buffer (20 mM of HEPES pH 7.4, 150 mM of KCl, and 1 mM of MgCl_2_) and 0.1 mM of GTP. Afterwards, 70 µl of the GUVs suspension was added to get a final volume of 300 µl. Confocal imaging was performed after 60 min incubation at room temperature. Images were processed with a homemade analysis software (Hermann *et al*, 2014) detecting the filling and the size of each GUVs. Per sample, well 100–200 GUVs were analyzed. The experiment was performed four times and the results are presented as mean ± SD. Levels of significance were determined by paired two‐tailed Student's *t*‐test and a confidence level of greater than 95% (*P* < 0.05) was used to establish statistical significance.

### Flow cytometry

Flow cytometry experiments were conducted using CytoFlex, and data were analyzed using the FACSDiva Software (Beckman Coulter). DRP1 binding per normalized liposome and membrane tethering was described previously (Ugarte‐Uribe *et al*, 2014). Shape index values (*y* axis) above 1 are indicative of an increase (negative membrane curvature or tethering) in a rhodamine‐derived signal averaged for all liposome size gates. The experiment was performed three times in duplicate and the results are presented as mean ± SD. Levels of significance were determined by paired two‐tailed Student's *t*‐test and a confidence level of greater than 95% (*P* < 0.05) was used to establish statistical significance.

### GTPase activity assay

The GTPase activity of DRP1 was assayed using a colorimetric assay as described in Ugarte‐Uribe *et al* (2014). Briefly, 0.5 μM of DRP1 was added to 1 mM of GTP in the absence or presence of BAX (0.25, 0.5, or 1 µM) and/or LUVs (150 µM) over a 20 to 180 min time course at 37°C in GTPase buffer (20 mM of HEPES/KOH pH 7.4, 150 mM of NaCl, and 1 mM of MgCl_2_). Reactions were stopped at the indicated times by diluting 20 μl of the sample in 100 mM of EDTA (final concentration) in a microtiter plate. Samples were then incubated with 150 μl of malachite green stock solution (1 mM of malachite green and 10 mM of ammonium molybdate in 1 N HCl), and the absorbance at 620 nm was determined using an Infinite M200 microplate reader (Tecan, Mainz, Germany). The experiment was performed four times in duplicate and the results are presented as mean ± SD.

### Solid‐phase synthesis of BAX peptides and DRP1 binding assay in GUVs

N‐terminally biotinylated BAX peptides (with SGSG linker sequence) were synthesized by Fmoc‐based solid‐phase peptide chemistry using a Syro I synthesizer (MultiSynTech GmbH) as described in (Liokatis *et al*, 2012). They were 15‐mers with five‐residue overlap, spanning all residues of human BAX (isoform alpha, NP_620166). Biotin was coupled manually with 4‐fold molar excess and Pyoxim was used as activator of the reaction. Except one peptide (peptide no. 18) for which the synthesis was not successful, all peptides were obtained with an average purity of > 90%.

GUVs (70 μl from the electroformation chamber) composed of DOPC:DSPE‐PEG(2000) Biotin (8:2, molar ratio) and labeled with DiD were mixed in a Nunc™ Lab‐Tek™ 8‐well chamber slide (Thermo Scientific) at a final concentration of 0.65 μM streptavidin with 3 μM of the corresponding BAX peptide, 0.1 mM GTP and 0.5 μM DRP1‐AF488 and incubated for 1 h. This way, the peptides were anchored via streptavidin to the GUV surface. Binding of DRP1‐AF488 to the GUVs was visualized with confocal microscopy and analyzed by quantifying the radial intensity in individual GUVs using home built software (Hermann *et al*, 2014). Data analysis was performed from *n* = 3 independent experiments (with *n* = 100 vesicles each) in an automated and blinded fashion to avoid observer bias.

### Protein cross‐linking coupled to mass spectrometry

#### Crosslink sample preparation and LC‐MS/MS data acquisition

Recombinant BAX and DRP1 (final concentrations of 2 μM and 0.8 μM, respectively) were mixed with 1 mM of LUVs in GTPase buffer (20 mM HEPES/KOH pH 7.4, 150 mM NaCl and 1 mM MgCl_2_) containing 100 μM of GTP and incubated at 42°C for 30 min. Then cross‐linkers EDC or DSS were added at a final concentration of 25 mM and incubated for 1h (for EDC) and 45 min (for DSS) at room temperature. Then reactions were quenched for 15 min with Tris/HCl pH8 at a concentration of 50 nM (in case of EDC) and 60 mM (in case of DSS) and β‐mercaptoethanol was added at a final concentration of 20 mM. The samples were then run 1 cm into a denaturing SDS‐PAGE gel before being excised and digested in‐gel using trypsin and chymotrypsin as previously described (Borchert *et al*, 2010). The resultant peptides were analyzed on an EASY‐nLC 1200 (Thermo Fisher Scientific) coupled to Q Exactive HF mass spectrometer (Thermo Fisher Scientific). Peptides were loaded onto a 75 μm (ID), 20 cm column packed in‐house with reversed‐phase ReproSil‐Pur 120 C18‐AQ 1.9 μm resin (Dr. Maisch GmbH). Peptides were eluted using a 43 min linear gradient of solvent B (80% ACN in 0.1% formic acid) from 10 to 33% at 200 nl/min. Full‐scans were recorded between 300 and 1,650 Thompson at a resolution of 60,000 with an AGC target of 1E6. The 7 most intense ions from each full scan were selected for fragmentation (MS/MS) by higher‐energy collisional dissociation (HCD) using an NCE of 27 and an AGC target of 1E5 in 110 ms at a resolution of 60,000.

#### XL‐MS data analysis

Crosslinks were identified using pLink software (http://pfind.ict.ac.cn/software/pLink) (Fan *et al*, 2015). Mass error tolerance for precursors was set to 5 ppm for 5 isotopes including the monoisotopic mass. Minimum peptide length was set to 6 amino acids with oxidation (Met) and carbamidomethylation (Cys) set as variable and fixed modifications respectively. Both trypsin and chymotrypsin cleavage sites were defined for the digestion enzyme and 5 missed cleavages were allowed. Digestion efficiency and raw mass accuracy were determined in a dedicated processing using MaxQuant software (Cox & Mann, 2008) using default settings. Based on the MaxQuant analysis searching against the human BAX and DRP1 proteins and the complete *E. coli* uniprot database (4,313 sequences) a database containing the top 20 most abundant *E. coli* proteins as decoys and the two human proteins was constructed for the pLink search. The same approach was used to identify both EDC and DSS cross‐linked peptides. The interaction maps between complex proteins were generated via xiNET‐Crosslink Viewer (http://crosslinkviewer.org).

### Recruitment hierarchy of BAX and DRP1 to apoptotic mitochondria

To test the hierarchy of the recruitment of BAX and DRP1 to mitochondria in apoptosis, U2OS BAX/BAK DKO cells were seeded in 8‐well chambered cover glass µ‐slides (IBIDI) and transfected with 50 ng pEGFP‐A206K_BAX_C1, 50 ng pAcGFP‐C1‐mCherry‐DRP1, 50 ng pEGFP‐N1‐4xmt‐mTurquoise2 (Table 1) using 0.5 µl of Lipofectamine 2000 for 12 h. For visualizing BAX foci formation after down‐regulation of DRP1, U2OS BAX/BAK DKO cells depleted for DRP1 (see section siRNA‐mediated knock‐down) were transfected with 50 ng pEGFP‐A206K_BAX_C1 and 50 ng pEGFP‐N1‐4xmt‐mTurquoise2 (Table 1). Before imaging the growth medium was changed to DMEM without phenol red and apoptosis was induced using 1 µM ABT‐737, 1 µM S63845 and 10 µM Q‐VD‐OPh (Hölzel Biotech) to inhibit effector caspases. Three‐dimensional time‐lapse confocal microscopy of individual cells was performed with a time resolution of 1 min for a duration of 30 min or until foci formation was observed. Maximum intensity z‐projection images were cropped and adjusted in brightness and contrast using Fiji/ImageJ (Schindelin *et al*, 2012).

### Induced dimerization experiments

The induced dimerization system (iDimerize™ Inducible Heterodimer System, Takara Bio) is based on the dimerization of the FK506‐binding protein (FKBP) and the FKBP rapamycin‐binding domain of mTOR (FRB) in the presence of rapamycin analogues (Bayle *et al*, 2006). BAX (or mutation variants of BAX), DRP1, and TOM20 were fused to the dimerization domains FKBP (also called dimerization domain A, DmrA) and/or FRB (dimerization domain C, DmrC) in order to artificially induce their dimerization using A/C Heterodimerizer (AP21967, Takara Bio). U2OS BAX/BAK DKO or HCT OctaKO cells were seeded in 8‐well chambered cover glass µ‐slides (IBIDI) and transfected with 50 ng pFRB‐EGFP‐BAX (or mutation variants of BAX) and 100 ng pFKBP‐mCherry‐DRP1, 100 ng pFRB‐mCherry‐DRP1 and 100 ng pFKBP‐mCherry‐DRP1, 50 ng pFRB‐EGFP‐BAX and 50 ng pFKBP‐EGFP‐BAX or 100 ng pAcGFP‐TOM20‐mCherry‐FKBP and 50 ng pFRB‐EGFP‐BAX (Table 1) for 16 h before imaging using 0.3 µl Lipofectamine 2000. Effects of induced dimerization were analyzed using confocal microscopy after changing the growth medium to DMEM without phenol red containing 300 nM A/C Heterodimerizer. Mitochondria were stained using 100 nM MitoTracker Deep Red TM (Invitrogen, Germany) or 10 nM MitoSpy TM NIR DiIC1(5) (BioLegend) to detect loss in mitochondrial membrane potential. Individual time‐lapse z‐stacks of 10–15 images with an z‐interval of ~ 350 nm were acquired with a time resolution of 5 min for 1 h. Maximum intensity z‐projection images were cropped and modified in brightness and contrast using Fiji/ImageJ (Schindelin *et al*, 2012). Altered mitochondrial morphology and mitochondrial depolarization was quantified visually in cells transfected with both constructs (FRB‐EGFP‐BAX and FKBP‐mCherry‐DRP1) before and after induced dimerization form *n* = 3 independent experiments (with *n* = 100 cells each) in a blinded fashion to avoid confirmation bias. Levels of significance were determined using Student’s *t*‐test. Pearson's correlation coefficient was calculated between the induced dimerization signal of BAX/BAX, DRP1/DRP1 and BAX/TOM20, respectively, and mitochondria based on MitoTracker Deep Red TM signal using the JACoP plugin (Bolte & Cordelieres, 2006) (https://imagej.nih.gov/ij/plugins/track/jacop2.html) in Fiji/ImageJ (Schindelin *et al*, 2012).

### Cell death analysis

U2OS BAX/BAK DKO cells were seeded in a 48‐well plate and transfected with 50 ng pFRB‐EGFP‐BAX and 100 ng pFKBP‐EGFP‐DRP1 or 50 ng GFP‐BAX (or mutant versions of BAX) (Table 1) for 16 h. Cells were stained with DRAQ7 (Thermo Fischer Scientific) in a 1:3,000 dilution in DMEM to stain the DNA of dead cells. Dimerization of BAX and DRP1 was induced using 300 nM A/C Heterodimerizer (AP21967, Takara Bio) and apoptosis was induced using 1 µM ABT‐737 and 1 µM S63845 (Hölzel Biotech). Cell death and mitochondrial depolarization were measured using the IncuCyte bioimaging platform (Essen). Time‐lapse measurements of fluorescent intensity were performed every hour in nine positions of each well, which were averaged for analysis. Number of DRAQ7 positive cells was normalized to the number of transfected cells (based on GFP signal) using the *cell‐by‐cell* analysis mode. Data analysis was performed from *n* = 2 independent experiments each of them in technical duplicates. Levels of significance were determined using Student’s *t*‐test.

### Cytochrome c release assay and protein translocation to mitochondria

In order to measure release of cytochrome *c* from mitochondria or BAX/DRP1 translocation to mitochondria after forcing their dimerization, 5 × 10^5^ U2OS BAX/BAK DKO cells were transfected with 300 ng pFRB‐EGFP‐BAX and 600 ng pFKBP‐mCherry‐DRP1 (Table 1) for 6–8 h and treated with 300 nM A/C Heterodimerizer (AP21967, Takara Bio) to induce BAX/DRP1 dimerization or 1 µM ABT‐737, 1 µM S63845, and 10 µM Q‐VD‐OPh (Hölzel Biotech) to induce apoptosis for 2 h. For separation of mitochondria and cytosol, cells were harvested by trypsinization, washed in PBS, and permeabilized using permeabilization buffer (20 of mM HEPES/KOH pH7.5, 100 of mM sucrose, 2.5 mM of MgCl_2_, 100 mM of KCl, freshly added 0.025% (w/v) digitonin and protease inhibitor cocktail in PBS) for 10 min on ice. Cellular membranes were pelleted by centrifugation at 15,000 *g* for 10 min at 4°C. After removing the supernatant (cytosolic fraction), the membranes were solubilized using RIPA buffer. Protein levels were analyzed using western blot.

### Measurement of caspase 3/7 activation

For measurement of caspase activation after induced dimerization of BAX and DRP1, U2OS BAX/BAK DKO cells were seeded in a 48‐well plate and transfected with 50 ng FRB‐EGFP‐BAX and 100 ng pFKBP‐EGFP‐DRP1 (Table 1) for 16 h. Cells were treated with 300 nM A/C Heterodimerizer (AP21967, Takara Bio) to force BAX/DRP1 dimerization or 1 µM ABT‐737 and 1 µM S63845 (Hölzel Biotech) to induce apoptosis for a time‐course of 90 min. Caspase activation was measured at individual time points as indicated using the Image‐iT™ LIVE Red Caspase‐3 and ‐7 Detection Kit (Thermo Fischer Scientific) according to the manufacturers protocol and detected with the IncuCyte bioimaging platform (Essen). Percentage of caspase activation was quantified by measuring the area of thresholded red fluorescence intensity normalized to the area of transfected cells (based on GFP fluorescence) of *n* = 2 independent experiments.

### Detection of BAX activation (exposure of N‐terminal 6A7 epitope)

For detection of BAX activation after induced dimerization of BAX and DRP1, the exposure of the N‐terminal 6A7 motif of BAX was detected by immunofluorescence confocal microscopy. U2OS BAX/BAK DKO cells were seeded on round 15 mm 1.5H glass coverslips and transfected with 150 ng FRB‐EGFP‐BAX and 300 ng FKBP‐mCherry‐DRP1 (Table 1) for 16 h. Cells were treated with 300 mM A/C Heterodimerizer (AP21967, Takara Bio) to force BAX/DRP1 dimerization or 1 µM ABT‐737, 1 µM S63845, and 10 µM Q‐VD‐OPh (Hölzel Biotech) for 40 min to induce apoptosis. Cells were fixed with prewarmed 4% (v/v) PFA in DMEM for 8 min at room temperature, washed 3x with PBS for 5 min, and permeabilized using 0.25% (v/v) Triton™ X‐100 in PBS. After blocking with 3% (w/v) BSA in PBS for 45 min at room temperature, the cells were incubated with α‐BAX(6A7) primary antibody (Table 1) for 16 h at 4°C, washed 3x with PBS for 5 min, and incubated with CF680‐coupled secondary antibody (Table 2) for 1 h at room temperature. Coverslips were washed 3x with PBS, mounted using ProLong™ Gold antifade reagent, and imaged by confocal microscopy. Representative images were chosen from *n* = 3 independent experiments.

## Author contributions

**Andreas Jenner:** Formal analysis; Investigation; Visualization; Methodology; Writing—review and editing. **Aida Peña‐Blanco:** Formal analysis; Investigation; Methodology; Writing—review and editing. **Raquel Salvador Gallego:** Formal analysis; Investigation; Visualization; Methodology; Writing—review and editing. **Begoña Ugarte‐Uribe:** Formal analysis; Investigation; Visualization; Methodology; Writing—review and editing. **Cristiana Zollo:** Formal analysis; Investigation; Visualization; Methodology. **Tariq Ganief:** Resources; Investigation; Visualization; Methodology. **Jan Bierlmeier:** Resources; Methodology. **Markus Mund:** Resources; Methodology; Writing—review and editing. **Jason E Lee:** Resources; Methodology. **Jonas Ries:** Resources; Supervision; Methodology; Writing—review and editing. **Dirk Schwarzer:** Resources; Supervision; Methodology; Writing—review and editing. **Boris Macek:** Resources; Supervision; Methodology; Writing—review and editing. **Ana J Garcia‐Saez:** Conceptualization; Supervision; Funding acquisition; Writing—original draft; Project administration; Writing—review and editing.

In addition to the CRediT author contributions listed above, the contributions in detail are:

AJ, AP‐B, BU‐U, RS‐G and CZ performed research and analyzed data. RS‐G did SMLM, FCS and ddFP experiments. BU‐U carried out *in vitro* activity experiments, peptide array and cross‐linking coupled to MS, as well as FCS experiments. AP‐B and CZ carried out ddFP experiments. AJ did recruitment and forced dimerization experiments. TG and BM did MS experiments and analyzed data. JEL contributed to ddFP experiments. MM and JR contributed to SMLM experiments. JB and DS prepared peptide array. AJG‐S conceived the project, designed and supervised research and wrote the manuscript with the help of all other authors.

## Supporting information



Expanded View Figures PDF

Source Data for Expanded View

## Data Availability

This study includes no data deposited in external repositories.
