# Supplementary material for: DRP1 interacts directly with BAX to induce its activation and apoptosis
Source: EMBO J. 2022 Jan 13;41(8):e108587. doi: 10.15252/embj.2021108587 (PMC9016351; doi:10.15252/embj.2021108587)
Supplement: Supplementary file 1 — Expanded View Figures PDF [file EMBJ-41-e108587-s003.pdf]

Expanded View Figures

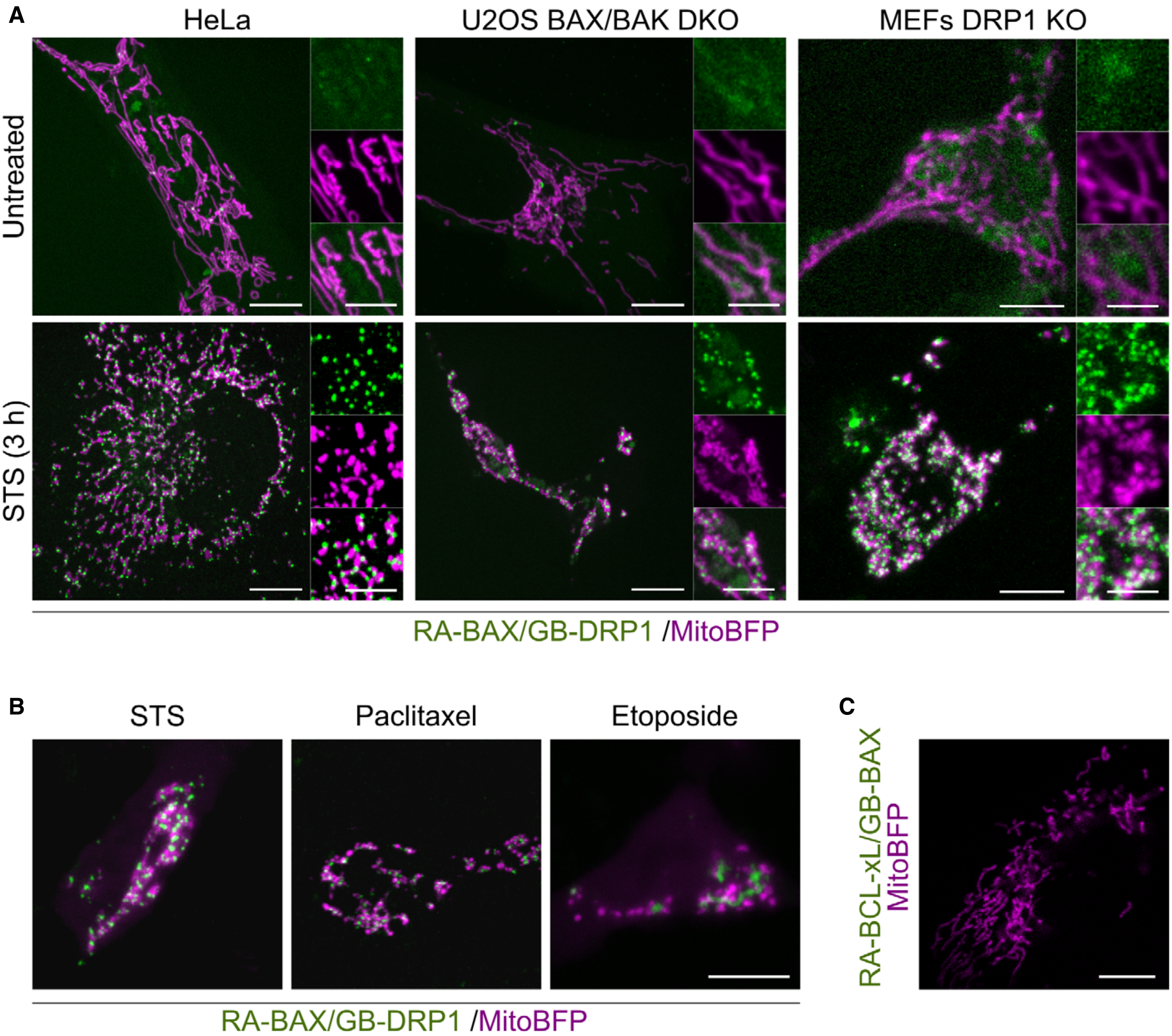

Figure EV1. Interaction of BAX and DRP1 is independent of cell type and apoptotic stimuli.

A Representative confocal microscopy images of ddFP of RA-BAX and GB-DFP1 (green) in untreated or apoptotic (STS) HeLa (same images as used in Fig 1E), U2OS BAX/BAK DKO, and MEF DRP1 KO cells. Mitochondria are visualized using mito-BFP (magenta). Scale bar 10 μm. Right panels are zoomed areas representing individual and merged channels. Scale bar 5 μm.

B Representative confocal microscopy images of RA-BAX/GB-DRP1 (green) and mito-BFP (magenta) overexpressed in U2OS BAX/BAK DKO cells after apoptosis induction using STS, Paclitaxel or Etoposide as indicated. Scale bar 10 μm.

C Representative confocal microscopy image of RA-BCL-xL/GB-DRP1 (green) and mito-BFP (magenta) overexpressed in U2OS BAX/BAK DKO cells after apoptosis induction using STS. Scale bar 10 μm. Images are representative for $n = 3$ independent experiments.

Data information: Images are representative for $n = 3$ independent experiments.

© 2022 The Authors

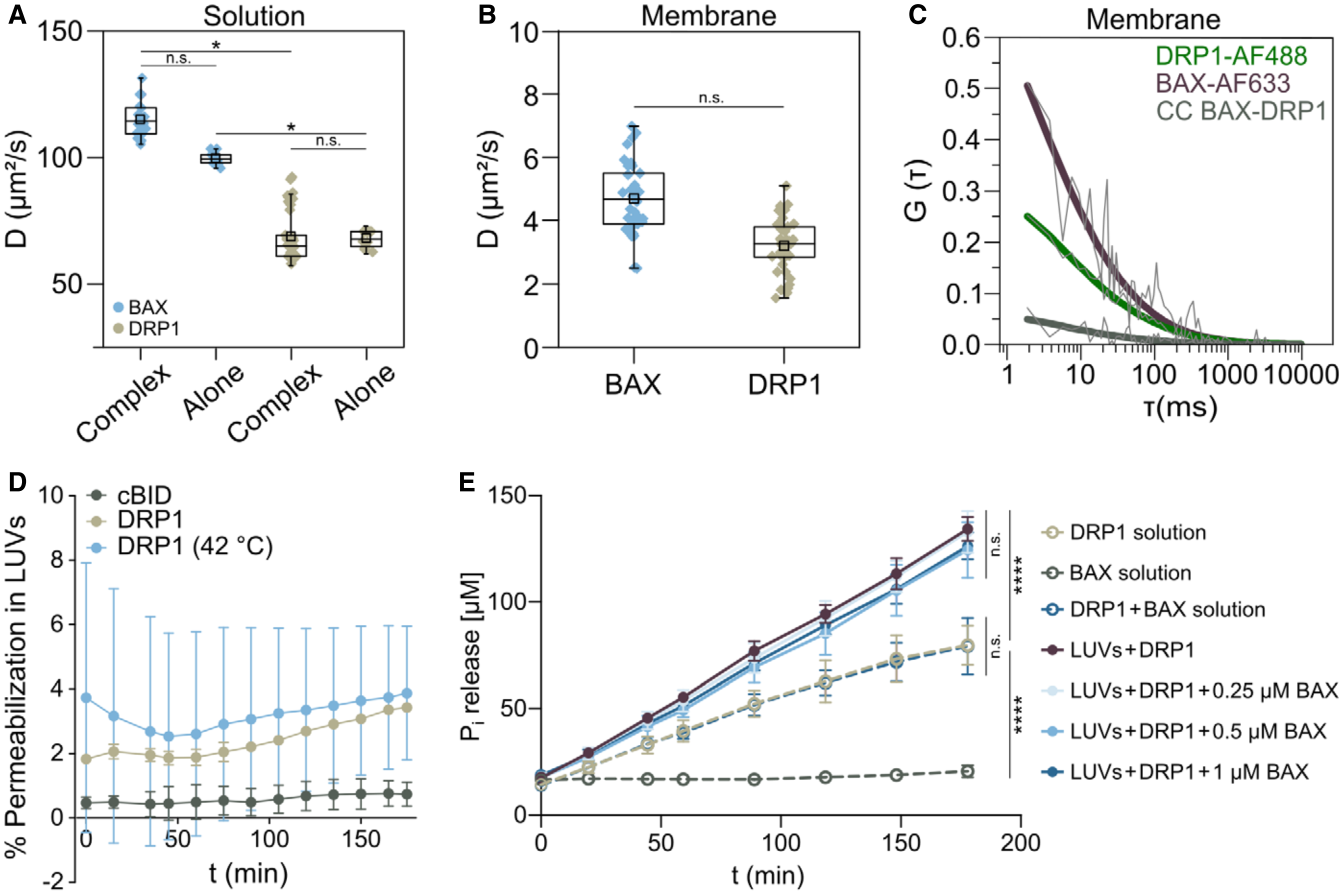

Figure EV2. Interaction between BAX and DRP1 and activity analysis *in vitro*.

A, B Diffusion coefficients derived from FCCS analysis of BAX-AF633 (blue) and DRP1-AF488 (beige) in solution in complex with each other and alone as indicated (A) or in the membrane (B). Box plots (in A and B) represent the interquartile (outer box), mean (inner box), median (line) and range (whiskers). Levels of significance were determined by paired two-tailed Student's *t*-test (n.s. $P > 0.05$, *$P < 0.05$) from $n \geq 25$ individual measurements for BAX or DRP1 in complexes and $n \geq 10$ measurements of BAX and DRP1 alone in solution (A) or $n \geq 33$ individual measurements for BAX or DRP1 in the membrane (B).

C Representative auto- (green and violet curves) and cross-correlation (CC, BAX-DRP1, grey curves) curves of DRP1-AF488 and BAX-AF633 measured in the membrane of GUVs in presence of unlabeled cBID. Thin grey lines depict raw data and thick lines correspond to data fitting.

D Negative control of LUV permeabilization by cBID and DRP1 alone. Calcein release was measured in calcein-encapsulated LUVs incubated with cBID (grey) or DRP1 at room temperature (beige) or after mild heating (42°C, blue). Data are presented as mean ± SD of $n = 3$ independent experiments.

E Effect of BAX on the GTPase activity of DRP1 in solution or in the membrane. Time course of GTP hydrolysis by DRP1 measured at 37°C in the absence or presence of LUVs and different concentrations of BAX as indicated. Data are presented as mean ± SD of $n = 4$ independent experiments. Significance was determined by paired two-tailed Student's *t*-test (n.s. $P > 0.05$, ****$P < 0.0001$).

© 2022 The Authors

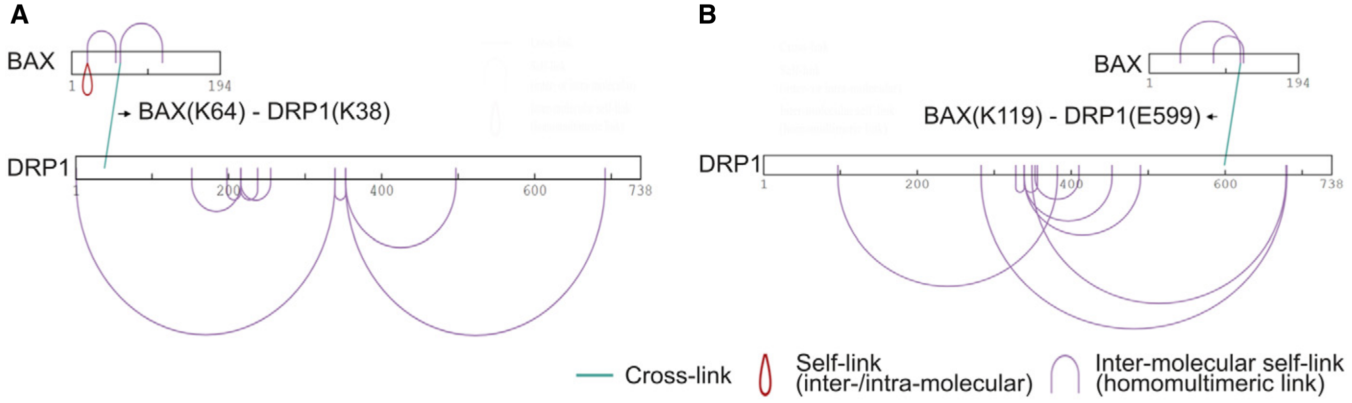

C

Peptide: SECLKRIGDELDSNMEL(5)-QSSGKSSVLESL(5)

Mod_Sites	Score	Calc_Mass	Delta_Mass	ppm	Proteins
3,C (Carbamidomethyl_C)	3,46E-03	3366,6169	-0,00071	-0,21083	BAX(64)-DRP1(38)

20170215_CO_0667AnGS_R18.14327.14327.4 File:"20170215_CO_0667AnGS_R18.raw", scan=14327"

D

Peptide: FYFASKL(6)-SKAEEL(4)

Mod_Sites	Score	Calc_Mass	Delta_Mass	ppm	Proteins
null	1,44E-03	1531,7921	-0,00447	-2,916	BAX(119)-DRP1(599)

20170215_CO_0667AnGS_R15.4135.4135.4 File:"20170215_CO_0667AnGS_R15.raw", scan=4135"

Figure EV3. pLink analysis of all crosslinks identified by XL-MS for BAX and DRP1 interaction in liposomes.

A, B Graphic representation of all positively identified crosslinks in the BAX/DRP1 complex mediated by DSS (A) and EDC (B).

C, D Excerpt from the pLink search result for the BAX(64)-DRP1(38) crosslinked by DSS (C) and BAX(K119)-DRP1(K599) crosslinked by EDC (D).

 © 2022 The Authors

Figure EV4. Expression, localization and apoptotic activity of BAX mutants not interacting with DRP1 and effect of MAPL-mediated DRP1 SUMOylation on the interaction.

A Confocal microscopy images of U2OS BAX/BAK DKO cells transfected with GFP-BAX mutants L63E, Δ19–37 or ΔL1–2 as indicated (green) and stained with MitoTracker Deep Red FM to visualize mitochondria (magenta). Scale bar 10 μm. Images are representative for $n = 3$ independent experiments.

B Quantification of cell death induced by overexpression of GFP-BAX mutants L63E, Δ19–37 and ΔL1–2 compared to wild type (WT) in U2OS BAX/BAK DKO cells. Data are presented as fold increase of dead cells 12 h after transfection normalized to the number of dead cells at the time point of transfection. Values correspond to mean (line) ± SD of $n = 2$ independent experiments. Single data points represent individual measurements from technical and biological replicates.

C Representative western blot analysis ($n = 3$ independent experiments) of total cell lysates from U2OS BAX/BAK DKO cells overexpressing GFP-BAX mutants (as indicated) probed against GFP. Whole cell protein staining (Ponceau S) is shown to confirm equal loading.

D Structural model of BAX highlighting proposed interaction regions for DRP1 and for the BH3 domain of BID. BAX structure is based on the NMR structure of the inactive, full-length monomer, PDB 1F16. In red, the C-terminal helix of BAX occupying the hydrophobic groove, which corresponds to the binding site of the BH3 helix of BID. The regions corresponding to residues 19-37 (in helix 1, light blue), the loop between helices 1 and 2 (blue) and L63 (yellow) are required for the interaction with DRP1.

E Confocal microscopy image of apoptotic U2OS BAX/BAK DKO cells transfected with RA-BAX/GB-DRP1 (green) and mito-BFP (magenta) after siRNA-mediated depletion of MAPL (left panel, siMAPL) versus siRNA control (right panel, siControl). Scale bar 10 μm.

F Western blot analysis of total cell lysates from U2OS BAX/BAK DKO cells after siRNA-mediated depletion of MAPL (siMAPL) versus siRNA control (siControl) probed against MAPL. Whole cell protein staining (Ponceau S) is shown to confirm equal loading. Data are representative of $n = 3$ independent experiments.

G Quantification of BAX/DRP1 interaction after siRNA-mediated MAPL knock-down (siMAPL) versus knock-down control (siControl) in healthy or apoptotic conditions (+/− STS). Box plots represent the interquartile (box), median (line) and SD (whiskers) of $n = 3$ independent experiments. Significance was tested by paired two-tailed Student's t-test (n.s. indicating $P > 0.05$).

Source data are available online for this figure.

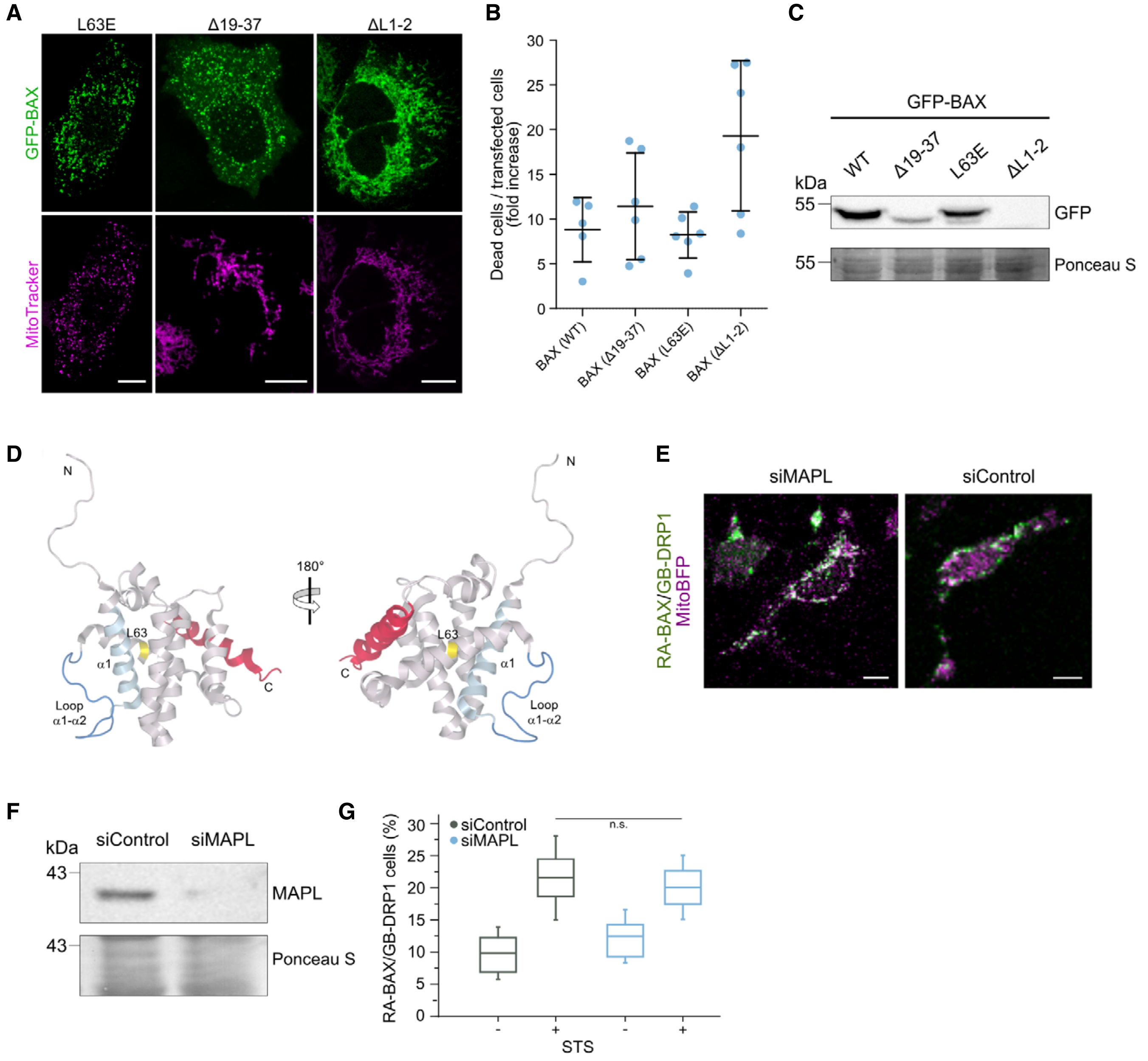

Figure EV4.

 © 2022 The Authors

Figure EV5. Induced dimerization of BAX and DRP1 induces apoptosis.

A Confocal microscopy of U2OS BAX/BAK DKO cells transfected with FKBP-mCherry-DRP1 (red) and FRB-EGFP-BAX (green) and stained with MitoTracker Deep Red FM (cyan). Images were acquired before (0 min) and after induction of BAX/DRP1 dimerization (5, 10 min). Scale bar 20 µm.

B–D Induced dimerization of DRP1 to itself (B), BAX to itself (C) or BAX to TOM20 (D) in U2OS BAX/BAK DKO cells transected with FKBP- and FRB-mCherry DRP1 (B, red), FKBP- and FRB-EGFP-BAX (C, green) or TOM20-mCherry-FKBP (red) and FRB-EGFP-BAX (green, D) before (0 min) and after induced dimerization (10 min). Mitochondria were stained using MitoTracker Deep Red FM or MitoSpy NIR (cyan) as indicated. Scale bar 20 µm. Pearson's correlation coefficient was calculated between the induced dimerization signal of BAX/BAX, DRP1/DRP1 and BAX/TOM20, respectively, and mitochondria based on MitoTracker Deep Red TM signal as depicted below the images.

E Induced dimerization of DRP1 and BAX mutants L63E, Δ19–37 or ΔL1–2. Confocal microscopy of U2OS BAX/BAK DKO cells transfected with FKBP-mCherry-DRP1 (red) and mutant variants FRB-EGFP-BAX (green) as indicated and stained with MitoTracker Deep Red FM (cyan). Images were acquired 10 min after induction of BAX/DRP1 dimerization. Scale bar 20 µm. All images are representative of $n = 3$ independent experiments.

F–H Dimerization of BAX and DRP1 induces their translocation to mitochondria, exposure of the BAX 6A7 epitope and cytochrome *c* release. (F) Western blot analysis of cytosolic and mitochondrial fraction from U2OS BAX/BAK DKO overexpressing FKBP-mCherry-DRP1 and FRB-EGFP-BAX after induction of BAX/DRP1 dimerization (Dim) or apoptosis (Apo) compared to non-treated (Ctrl) or untransfected cells (Untr). Western blot was probed against DRP1 and BAX. Mitofilin (IMMT) was used to test for purity of cytosolic fraction. Results are representative for $n = 3$ independent experiments. (G) Representative ($n = 3$ independent experiments) confocal microscopy images of U2OS BAX/BAK DKO cells transfected with FKBP-mCherry-DRP1 (red) and FRB-EGFP-BAX (green). Cells were immunostained against the 6A7 epitope of BAX (grey) after induced dimerization of BAX and DRP1 (Dimerizer) compared to untreated (Control). Images show individual channels or overlay of FKBP-mCherry-DRP1 and BAX-(6A7) immunofluorescence signal. Scale bar 10 µm. (H) Representative ($n = 3$ independent experiments) western blot analysis of the cytosolic and mitochondrial fraction from U2OS BAX/BAK DKO treated as described in F) and probed against cytochrome *c* (Cytc). TOM20 was used to test for purity of cytosolic fraction. Protein staining (Ponceau S) is shown to confirm equal loading.

I–K Quantification of caspase activation, PARP cleavage and cell death induced by forced dimerization of BAX and DRP1. (I) U2OS BAX/BAK DKO cells were transfected with FKBP-mCherry-DRP1 and FRB-EGFP-BAX and analyzed for the percentage of cells with caspase 3/7 activation. Data are normalized to the number of transfected cells at individual time points after inducing dimerization of BAX and DRP1 (Dim, 30, 60 and 90 min) compared to apoptosis induction for 90 min (Apo, 90 min) or untreated cells (Ctrl). (J) Representative western blot analysis ($n = 3$ independent experiments) of total cell lysates from cells transfected as described in (I) after induction of BAX/DRP1 dimerization (Dim) or apoptosis (Apo) compared to non-treated (Ctrl) or untransfected cells (Untr). Blot was probed against PARP or GAPDH as a loading control. K) U2OS BAX/BAK DKO cells were transfected as described in (I) and induced for BAX/DRP1 dimerization (Dim) or apoptosis (Apo) compared to untreated (Ctrl). Data are presented as fold increase of dead cells 1 h after treatment compared to the number of dead cells before treatment and normalized to the number of transfected cells.

Data information: Values in (I) and (K) are representative of $n = 2$ independent experiments and correspond to mean (bar, line) ± SD. Single data points in (I) represent individual measurements from technical and biological replicates.
Source data are available online for this figure.

© 2022 The Authors

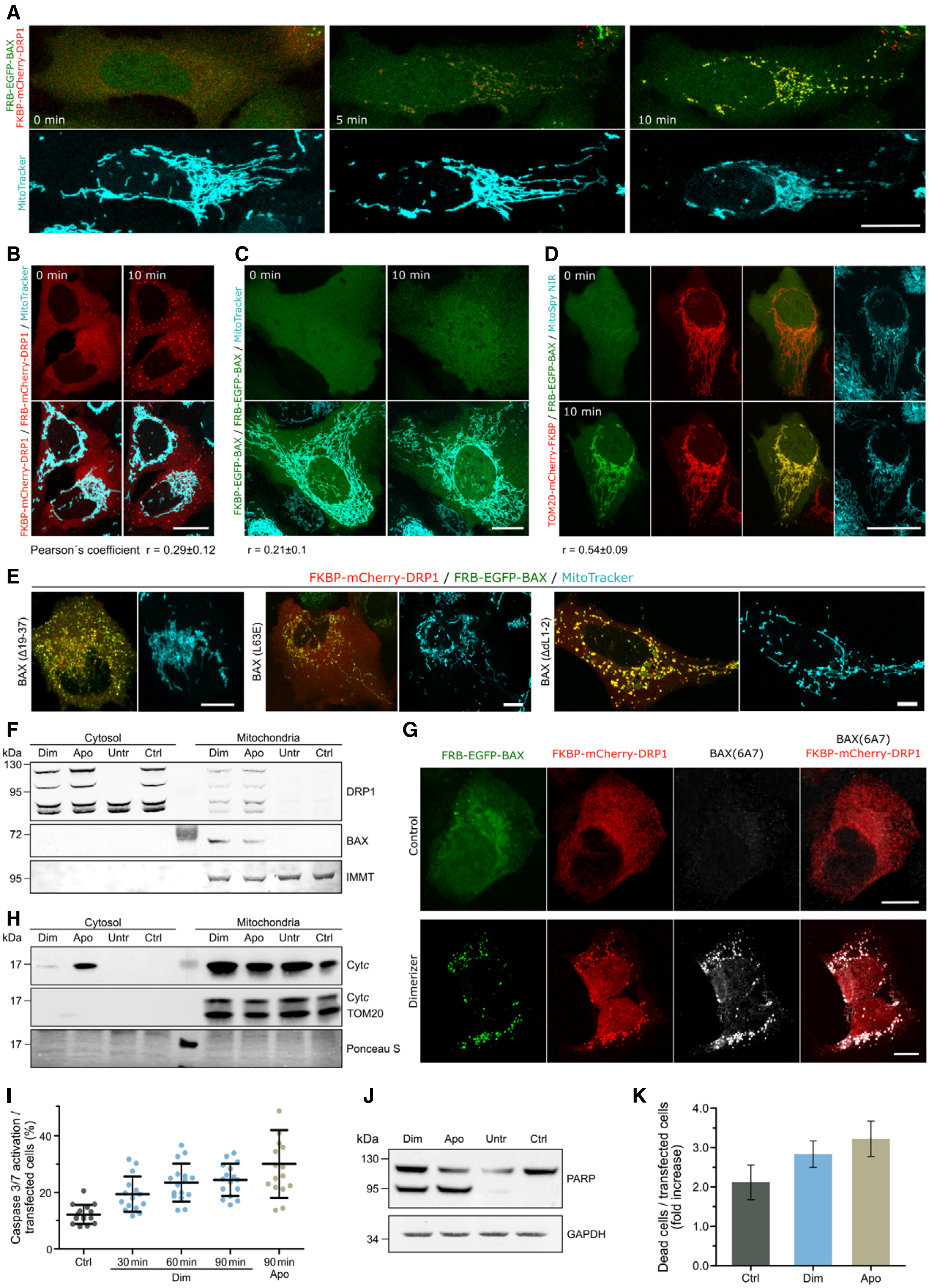

Figure EV5.

 © 2022 The Authors

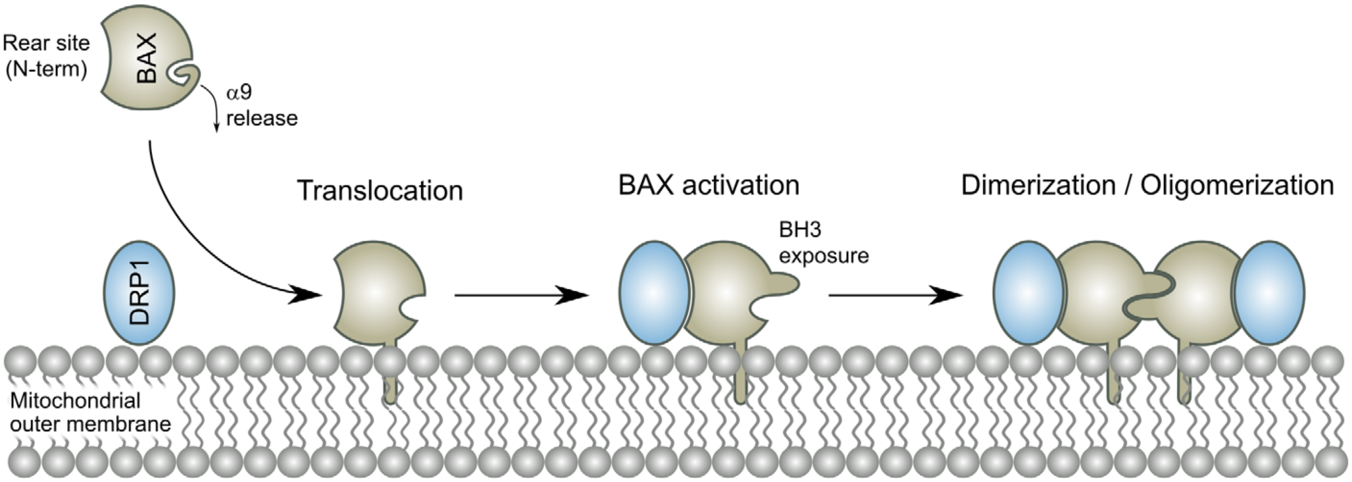

Figure EV6. Model for the interaction between BAX and DRP1 in apoptosis.

During apoptosis, DPR1 binds to mitochondrial BAX via multiple interfaces, with the N-terminus of BAX being required for the interaction. The interaction between BAX and DRP1 promotes BAX activation and apoptosis induction.

© 2022 The Authors
