# Supplementary figures and images for: DRP1 interacts directly with BAX to induce its activation and apoptosis
Source: EMBO J. 2022 Jan 13;41(8):e108587. doi: 10.15252/embj.2021108587 (PMC9016351; doi:10.15252/embj.2021108587)

## EV4 C

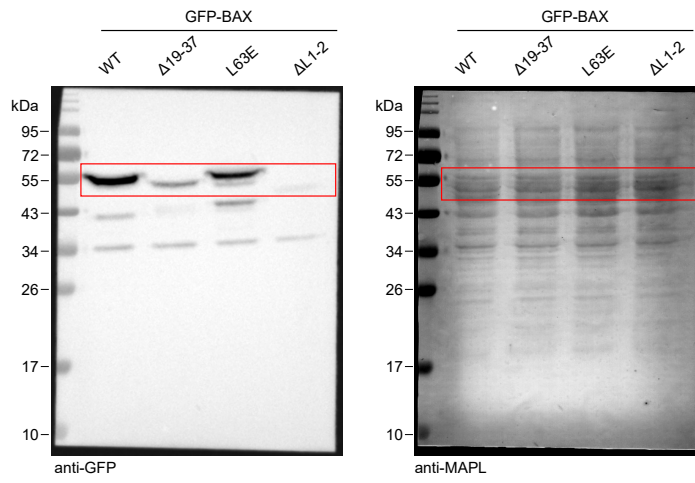

## EV4 F

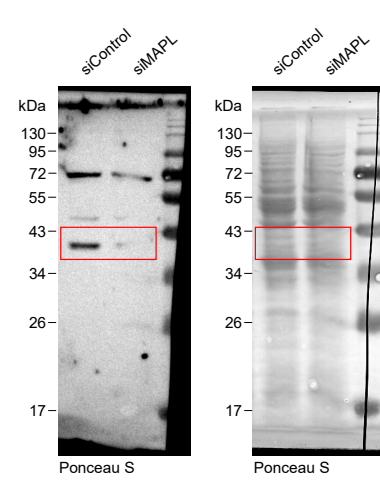

Supplement: Supplementary file 2 — Source Data for Expanded View [file EMBJ-41-e108587-s002.zip › EV_Figure_Source_Data/EMBOJ-2021-108587R1-Figure_Source_Data_EV4-sd.pdf]

EV5 F

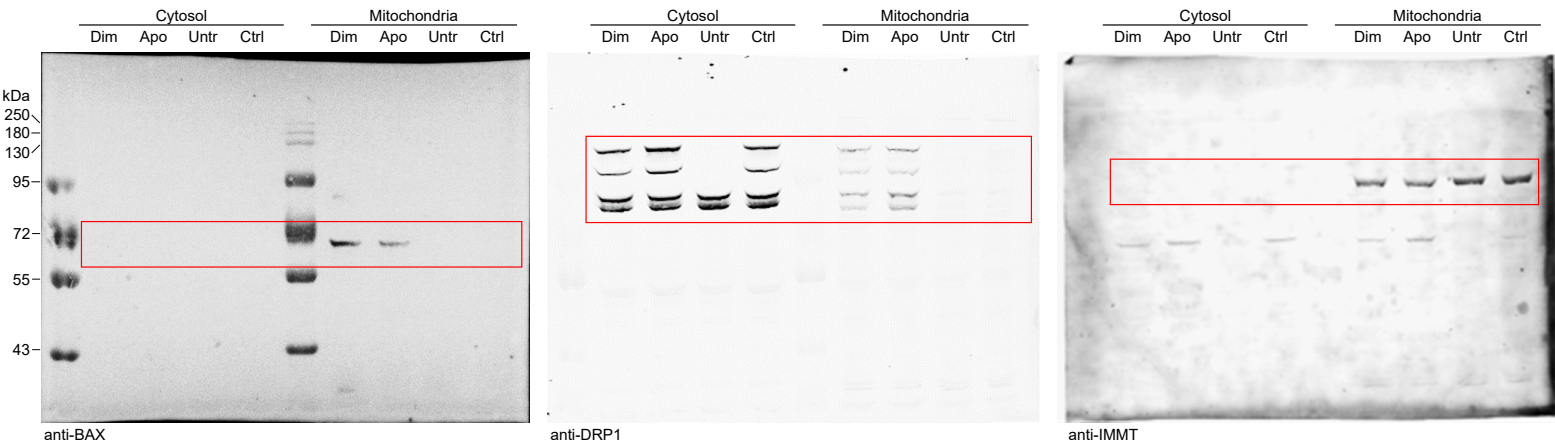

EV5 H

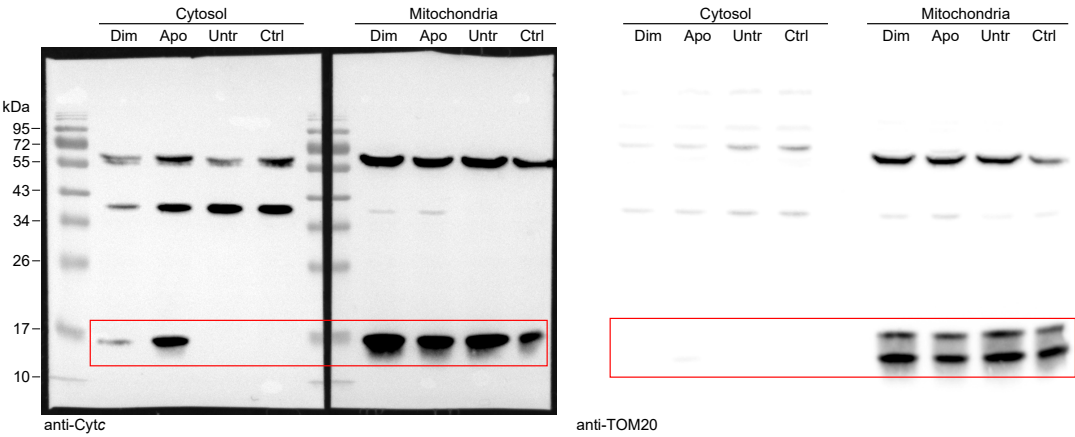

EV5 J

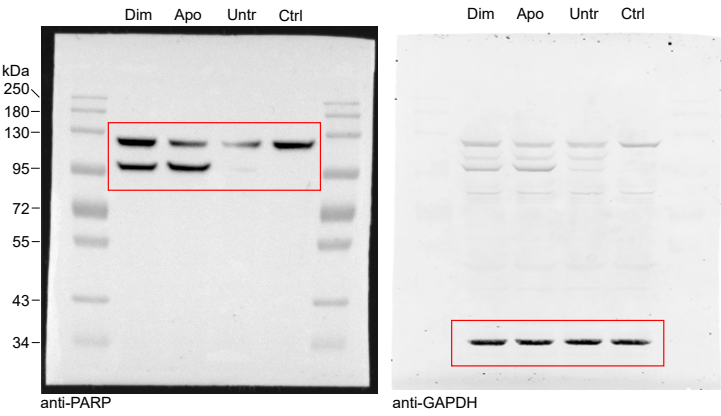

Supplement: Supplementary file 2 — Source Data for Expanded View [file EMBJ-41-e108587-s002.zip › EV_Figure_Source_Data/EMBOJ-2021-108587R1-Figure_Source_Data_EV5-sd.pdf]
