# Supplementary material for: DRP1 interacts directly with BAX to induce its activation and apoptosis
Source: EMBO J. 2022 Jan 13;41(8):e108587. doi: 10.15252/embj.2021108587 (PMC9016351; doi:10.15252/embj.2021108587)
Supplement: Supplementary file 3 [file EMBJ-41-e108587-s001.pdf]

THE EMBO JOURNAL

Direct interaction with DRP1 activates BAX and induces apoptosis

Andreas Jenner, Aida Peña-Blanco, Raquel Salvador Gallego, Begoña Ugarte-Uribe, Cristiana Zollo, Tariq Ganief, Jan Bierlmeier, Markus Mund, Jason Lee, Jonas Ries, Dirk Schwarzer, Boris Macek, and Ana Garcia-Saez
DOI: 10.15252/embj.2021108587

Corresponding author(s): Ana Garcia-Saez (ana.garcia@uni-koeln.de)

Review Timeline:

Submission Date:	28th Apr 21
Editorial Decision:	21st May 21
Revision Received:	22nd Sep 21
Editorial Decision:	8th Nov 21
Revision Received:	1st Dec 21
Accepted:	3rd Dec 21

Editor: Elisabetta Argenzio/Karin Dumstrei

Transaction Report:

(Note: With the exception of the correction of typographical or spelling errors that could be a source of ambiguity, letters and reports are not edited. Depending on transfer agreements, referee reports obtained elsewhere may or may not be included in this compilation. Referee reports are anonymous unless the Referee chooses to sign their reports.)

Dear Ana,

Thank you for submitting your manuscript entitled "Direct interaction with DRP1 activates BAX and induces apoptosis" [EMBOJ-2021-108587] to The EMBO Journal. Your study has now been assessed by three reviewers, whose reports are enclosed below.

As you can see, the referees concur with us on the potential interest of your findings. However, they also raise several critical points that need to be addressed before they can support publication here.

Given the overall interest of your study, I am pleased to invite submission of a manuscript revised as indicated in the reports attached herein. I would like to point it out that addressing all referees' points in a conclusive manner, as well as a strong support from the reviewers, would be essential for publication in The EMBO Journal. I should also add that it is our policy to allow only a single round of major revision. Therefore, acceptance of your manuscript will depend on the completeness of your responses in this revised version.

We generally grant three months as standard revision time. As we are aware that many laboratories cannot function at full capacity owing to the COVID-19 pandemic, we may relax this deadline. Also, we have decided to apply our 'scooping protection policy' to the time span required for you to fully revise your manuscript and address the experimental issues highlighted herein. Nevertheless, please inform us as soon as a paper with related content is published elsewhere.

I realize that addressing all the referees' criticisms will require time and additional efforts that might also be technically challenging. I would therefore understand if you were to choose not to undergo an extensive revision here and rather pursue a submission elsewhere, in which case please inform us about your decision at your earliest convenience.

When preparing your letter of response to the referees' comments, please bear in mind that this will form part of the Review Process File and will therefore be made available online. For more details on our Transparent Editorial Process, please visit our website: http://emboj.embopress.org/about#Transparent_Process.

Before submitting your revised manuscript, deposit any primary datasets and computer code produced in this study in an appropriate public database (see http://msb.embopress.org/authorguide#dataavailability). Please remember to provide a reviewer password, in case such datasets are not yet public. The accession numbers and database names should be listed in a formal "Data Availability" section (placed after Materials & Method). Provide a "Data availability" section even if there are no primary datasets produced in the study.

I thank you again for the opportunity to consider this work for publication and look forward to your revision.

Best regards,

Elisabetta

Elisabetta Argenzio, PhD
Editor
The EMBO Journal

Instructions for preparing your revised manuscript:

Please make sure you upload a letter of response to the referees' comments together with the revised manuscript.

Please also check that the title and abstract of the manuscript are brief, yet explicit, even to non-specialists.

When assembling figures, please refer to our figure preparation guideline in order to ensure proper formatting and readability in print as well as on screen:
https://bit.ly/EMBOPressFigurePreparationGuideline

IMPORTANT: When you send the revision we will require
- a point-by-point response to the referees' comments, with a detailed description of the changes made (as a word file).
- a word file of the manuscript text.

- individual production quality figure files (one file per figure)
- a complete author checklist, which you can download from our author guidelines (https://www.embopress.org/page/journal/14602075/authorguide).
- Expanded View files (replacing Supplementary Information)
Please see out instructions to authors
https://www.embopress.org/page/journal/14602075/authorguide#expandedview

Please remember: Digital image enhancement is acceptable practice, as long as it accurately represents the original data and conforms to community standards. If a figure has been subjected to significant electronic manipulation, this must be noted in the figure legend or in the 'Materials and Methods' section. The editors reserve the right to request original versions of figures and the original images that were used to assemble the figure.

Further information is available in our Guide For Authors: https://www.embopress.org/page/journal/14602075/authorguide

Revision to The EMBO Journal should be submitted online within 90 days, unless an extension has been requested and approved by the editor; please click on the link below to submit the revision online before 19th Aug 2021:

https://emboj.msubmit.net/cgi-bin/main.plex

Referee #1:

Jenner and colleagues investigate the relationship between DRP1 and BAX during apoptosis. Using a variety of approaches, the authors find co-localization of DRP1 and BAX at foci in apoptotic cells, consistent with earlier findings. They proceed to demonstrate a direct interaction between BAX and DRP1 using different methodss, showing that this is dependent on membrane and the alpha 1 helix, alpha 1-2 loop in BAX. Using liposome assays they determine a role for this interaction in the activities of both proteins. Finally, they show that enforced dimerization of DRP1 and BAX is sufficient to cause BAX activation and cell death.

The study is rigorous, well presented and the results largely support the authors' conclusions. However, as discussed further, two major issues center on the lack of investigation that the DRP-1/BAX interaction is relevant for mitochondrial permeabilization (possibly is an epiphenomenon that occurs after the cell has committed to cell death) and alternative (possibly trivial) reasons for the conclusion that DRP1 is a direct activator of BAX.

- The role of mitochondrial fission/DRP-1 as having a causative role in apoptosis is controversial. The cell data presented here is consistent with an interaction between DRP-1 and BAX occurring, but this is only apparent when cells have already undergone mitochondrial permeabilisation (i.e. in mitochondrial associated apoptotic foci), suggestive of a consequence of cell death. This raises the question of the relevance of this interaction for the initial BAX activating step. Do the authors find a role for DRP-1 in BAX induced mitochondrial permeabilisation in these cells? Given the authors' have identified key regions of BAX required for DRP-1 activation, if upon deletion these do not perturb BAX activation per se (testable using liposomes) they would serve as useful mutants to test a role for DRP-1 in BAX activation in cells (re-expressing in BAX deleted cells).

- The implication that DRP-1 may be a direct activator of BAX is really exciting. The control to imply that this is a specific event is that enforced dimerization of BAX with TOMM20 has no effect. In my opinion, this is subject to overinterpretation, we don't know what the topology of TOMM20 associated BAX is relative to DRP-1 associated BAX, it could be that TOMM20 associated BAX is distal from the mitochondrial membrane (relative to DRP-1 associated BAX) and therefore less likely to be active. A better control would be to carry out similar experiments using the BAX deletion mutants described earlier that can no longer bind DRP1, in this case does DRP-1 still activate these, killing cells?

- A key experiment to support the authors' hypothesis (DRP-1 can directly activate BAX) will be to carry out similar enforced dimerization experiments in cells lacking BH3-only (activator) proteins, HCT116 OCTA ko cells described elsewhere (PMID 27056669)

Referee #2:

There is a long-lasting question in the apoptosis field whether the pro-fission Drp1 protein is actively involved in the apoptosis process. In this paper, Jenner et al elegantly demonstrate using sophisticated tolls that Drp1 and the pro-apoptotic Bcl-2 family member Bax interact in lipid membranes and affect each other's activities in-vitro. They also mapped the interaction sites, and

used live cell imaging to demonstrate that Bax and Drp1 co-localize at discrete foci during apoptosis. Moreover, Jenner et al demonstrate that enforced dimerization of Drp1 and Bax trigger their translocation to mitochondria, accumulation at discrete sites, mitochondria fragmentation, and depolarization of mitochondria.

Thus, Jenner et al use many in-vitro tools and several tolls in intact cells - live cell imaging and enforced dimerization - to conclude that Drp1 interacts and activates Bax and induces apoptosis. Why does Bax require Drp1 if it has a super-efficient activator such as truncated tBid, as shown by the authors with tBid (cBID) in Fig 3? Does Drp1 compete with tBid or with Bax itself on binding to Bax? On the other hand, why does Drp1 require Bax for mitochondria translocation if it has a super-efficient receptor like MFF? Does Bax compete with MFF?

Despite these concerning issues, this reviewer is open to the authors' novel model that Drp1 plays an active role in Bax translocation/activation and vice versa, and if this model is correct, it will be an important contribution to our field. However, at this stage, this model is premature and there are several critical experiments to be performed in cells to ground it.

1) Do endogenous Drp1 and Bax directly interact in the intact mitochondrial membrane of apoptotic cells? This should be demonstrated by using cross-linkers as previously demonstrated for Bax homodimers/oligomers. Do Bax mutants that fail to cross-link to Drp1 in-vitro also fail to cross-link to Drp1 in intact mitochondria? Do these mutants possess reduced ability to induce apoptosis? I would also recommend using the Blue-native approach to strengthen this point.

2) The Korsmeyer group has demonstrated in 1998 (Gross et al, EMBO J) that "Enforced dimerization of BAX results in its translocation, mitochondrial dysfunction and apoptosis". Have the Korsmeyers reached the wrong conclusion? Probably not. It probably depends on how the fusion proteins are constructed and in the case of Jenner et al the dimerization of their fusion proteins did not result in Bax activation. In any case, I think it is important to cite the Gross et al paper and discuss it.

Moreover, to reach their conclusions, Gross at al used the following methods:
a) The FKBP/FK1012 dimerization system (and importantly FK506 to reverse the dimerization)
b) cross-linkers + Western blots
c) subcellular fractionations + Western blots (to monitor Bax translocation and cytochrome c release)
d) caspase activation assays
e) FACS analysis (to measure mitochondria membrane potential, reactive oxygen species (ROS) production and cell death)

Jenner at al should use similar approaches to prove that enforced dimerization of Bax and Drp1 results in their hetero-dimerization, translocation to mitochondria (using also Western blot analysis), N-terminal exposure of Bax, cyt c release, caspase activation, and FACS analysis to measure the parameters described in the Gross et al paper (and numerous additional papers). Jenner at al used MitoSpy NIR to measure mitochondria membrane potential and concluded that mitochondria depolarization is an indication of mitochondria outer membrane permeabilization (MOMP), which is incorrect.

3) Fig 1: In the ddFP experiments the authors are advised to measure the dimerization of Bax-Bax (RA-Bax and GB-Bax) and Bax-tBid (RA-Bax and GB-tBid) as positive references to assess the strength of the RA-Bax and GB-Drp1 dimerization. Why does DMSO inhibit the interaction between RA-Bax and GB-Drp1 (Fig 2C)?

4) Fig 2A: The representative pictures are confusing. The appearance of Drp1 and Bax interaction does not seem convincing since all the mitochondria are already collapsed. The increase in the intensity of Smac-GFP seems to come from signal accumulation on mitochondria, and not cytosol, of the dying cell, which seems to shine brighter. The experiments which include translocation of proteins from the cytosol to mitochondria should include markers for both compartments.

5) On page 9 (2nd paragraph from top) the authors conclude that "These results suggest that the binding between BAX and DRP1 in apoptosis is independent of the membrane constriction function of DRP1, for which the GTPase activity is required". Thus, it is possible that the function of Drp1 on the post-fissed mitochondria is to recruit Bax and this is the reason why their interaction is independent of Drp1's GTPase activity.

6) Figure 6, S1, S2, S5: add quantification of the images and statistics
Figure 1F, 2C, 2D, 3C, S3: mark statistically significant results and mention the level of significance in the legends
Figure 6D: mitochondrial fragmentation as well as mitochondrial membrane potential should be quantified
Figure 6E-G: perform co-localization analysis

Referee #3:

In manuscript EMBOJ-2021-108587 entitled "Direct interaction with DRP1 activates BAX and induces apoptosis" Andreas Jenner, Ana Garcia-Saez and colleagues revisit earlier observations regarding the mitochondrial interplay of BAX, an effector of MOMP, and DRP1, a mitochondrial fission executioner, through a high magnification lens of super-resolution microscopy. The new approach (single molecule localization microscopy) confirms that these proteins colocalize within 30 nM (the size of about 7

side-by-side BAX molecules). The authors back up this observation by dissecting their interaction biochemically and through fluorescence microscopy in vitro and in cells. The authors conclude that the two proteins influence each other's membrane activities, with DRP1 promoting permeabilization of vesicles (LUVs and GUVs) by BAX, and BAX promoting accumulation of DRP1 to membranes. The authors clarify some of the previous observations supporting a direct interaction of DRP1 and BAX, yet they do not exclude previous observations suggesting that DRP1-mediated membrane remodeling recruits BAX. The authors propose that DRP1 acts as BAX activator by interacting with the N-terminal portion of BAX, including helix 1, helix 2 and the loop joining them. The interplay between BAX and DRP1 at the mitochondria is important to understand as it may directly influence the threshold for MOMP in apoptosis initiation in pathophysiology. Once BAX induces MOMP it may promote accumulation of DRP1 to induce mitochondrial fission and drive the biology downstream of MOMP. The manuscript uses elegant approaches, flows logically, is well written, and will be significant in elucidating the relationship between mitochondrial apoptosis and mitochondrial fission. The following comments are meant to help the authors clarify several aspects of the findings:

1. The authors used the dimerization dependent fluorescent protein (ddFP) technique to monitor BAX-DRP1 interaction with a resolution of 10 nM, stating on page 5:
"As negative control, the combination of the anti-apoptotic BCL-2 protein BCL-xL tagged with RA and GB-DRP1 did not give any fluorescent signal neither in untreated nor in treated cells."
I could not find the data, but this negative control is very important and should be included in the manuscript.

2. The authors note delay in release of mitochondrial proteins through MOMP and appearance of BAX/DRP1 foci on page 6 related to Figure 2A and 2B:
"The short delay between the redistribution of Smac-GFP and the appearance of BAX/DRP1 complexes is comparable to that observed with the formation of GFP-BAX foci and Smac release during apoptosis (Salvador-Gallego et al., 2016)."
Figures 2A and 2B show that even at 270 min (timing in Figure 2A), when SMAC-GFP was almost completely released from the mitochondrial network (some perinuclear SMAC-GFP structure persist), there are no visible RA-BAX/GB-DRP1 foci. In Figure 2B it appears that foci are noted after 100 min. This implies that MOMP occurred but that the sensitivity of the ddFP assay precludes earlier detection of RA-BAX/GB-DRP1 foci. This discrepancy poses limitations in interpreting the functional consequences of BAX/DRP1 interactions at the earlier time points (<100 min). One way of overcoming this would be to identify DRP1 mutants that disrupt the interaction with BAX and test their kinetics of association relative to SMAC-GFP release. This could be clarified in future research, but the authors need to acknowledge this limitation in the discussion. As it stands, during initial time points after apoptosis induction DRP1-BAX complex is "invisible" by microscopy and one cannot definitively conclude that the two proteins interact.
Minor comments related to Figure 2A and 2B: Time scales could be match with the x-axis at 0 in Figure 2B. It may be useful to include the 100 min image in Figure 2A.

3. The authors do not show the relative levels of expression (immunoblotting) for the different constructs and mutants investigated in Figure 5, nor their apoptotic activities. Presumably they have this data, which would be useful in drawing mechanistic conclusions. For instance, are h1 deletion, L1-2 deletions, and L63E active? (Some of the other deletions may be more disruptive to BAX fold relevant to apoptosis function.) The full panel of constructs does not need to be investigated but it would be useful to test the three RA-BAX constructs functionally {plus minus} GB-DRP1.

4. Related to point 3, if active, how do any of the three constructs impaired in DRP1 binding perform in apoptotic response upon forced dimerization as presented in Figure 6?

5. Vesicle permeabilization studies suggest that DRP1 does not activate BAX but that it cooperates with BID and heat activated BAX in permeabilization. Yet, the authors conclude that DRP1 may act by directly binding at a site similar to the noncanonical site engaged by BIM SAHB, based on the peptide array, deletion, and mutagenesis analyses. Could the authors include a cartoon model showing the sites on BAX that may be engaged by BID and DRP1 to make this more accessible to readership outside the field.

Additional minor comments:

6. Pg 4. "Endogenous DRP1 was immunostained and labeled with the cyanine-based fluorescent (CF) dye CF680."
The authors could rewrite this sentence inferring that the secondary Ab was CF680 labeled.

7. The authors need to move Figure 2D and associated supplementary figure later in the manuscript when this panel is discussed on pages 9-10 (possibly move to Supplementary Figure S5).

8. Are BAX and DRP1 derivatized with AF probes on lysines or thiols? Please add these details in the methods. Derivatization at some sites may affect DRP1 binding and function.

9. The authors should include the cross-correlation data in panel 3B for membrane+BID experiment in panel 3C.

10. The authors need to mention what the rhodamine signal represents in Figure 3F.

POINT-BY-POINT ANSWERS TO THE REVIEWERS

Referee #1:

Jenner and colleagues investigate the relationship between DRP1 and BAX during apoptosis. Using a variety of approaches, the authors find co-localization of DRP1 and BAX at foci in apoptotic cells, consistent with earlier findings. They proceed to demonstrate a direct interaction between BAX and DRP1 using different methods, showing that this is dependent on membrane and the alpha 1 helix, alpha 1-2 loop in BAX. Using liposome assays they determine a role for this interaction in the activities of both proteins. Finally, they show that enforced dimerization of DRP1 and BAX is sufficient to cause BAX activation and cell death.

The study is rigorous, well presented and the results largely support the authors' conclusions. However, as discussed further, two major issues center on the lack of investigation that the DRP-1/BAX interaction is relevant for mitochondrial permeabilization (possibly is an epiphenomenon that occurs after the cell has committed to cell death) and alternative (possibly trivial) reasons for the conclusion that DRP1 is a direct activator of BAX.

We thank the reviewer for the positive evaluation of our work.

- The role of mitochondrial fission/DRP-1 as having a causative role in apoptosis is controversial. The cell data presented here is consistent with an interaction between DRP-1 and BAX occurring, but this is only apparent when cells have already undergone mitochondrial permeabilisation (i.e. in mitochondrial associated apoptotic foci), suggestive of a consequence of cell death. This raises the question of the relevance of this interaction for the initial BAX activating step. Do the authors find a role for DRP-1 in BAX induced mitochondrial permeabilisation in these cells? Given the authors' have identified key regions of BAX required for DRP-1 activation, if upon deletion these do not perturb BAX activation per se (testable using liposomes) they would serve as useful mutants to test a role for DRP-1 in BAX activation in cells (re-expressing in BAX deleted cells).

We agree with the reviewer that the causative role of DRP1 on apoptosis has remained controversial and we think this is largely due to the effects of altering DRP1 levels on mitochondrial function, and thereby indirectly on apoptosis sensitivity. The lag time between detection of BAX/DRP1 interactions and mitochondrial permeabilization is comparable to the lag time that we consistently observe between BAX-mediated SMAC release and the accumulation of GFP-BAX foci in mitochondria (Salvador-Gallego et al., EMBO J 2016). This suggests that the delay between the experimental observation of both processes is likely related to their relative efficiency of detection, since we need to achieve a strong signal-to-noise ratio. Nevertheless, and although we observe a clear temporal correlation, we cannot exclude that one happens before the other from these experiments. We have now explained this issue better in the text.

One of our initial goals to identify mutants of BAX that do not interact with DRP1 in cells was precisely to devise a way to address the functional relevance of the interaction. Although we indeed identified BAX mutants that do not bind to DRP1 in our experimental system, unfortunately all of these mutants had altered localization

patterns and apoptotic activity, thereby invalidating this approach (see answer to reviewer 3 below). As an alternative to BAX mutants, and also following the suggestion of reviewer 3, now we have also explored the effect of DRP1 mutations on the interaction with BAX. Concretely, we have studied the effect on the interaction with BAX of 5 mutant versions of DRP1 that affect different aspects of its activity. Unfortunately, we could not identify any DRP1 mutant with a clear-cut disruption of the ddFP signal with BAX (see Figure 5F-H). In summary, despite all our efforts, it has proven extremely difficult to identify BAX or DRP1 mutants that do not interact while retaining their activity. As a result, we cannot disentangle the effect on the BAX/DRP1 interaction from potential indirect effects on mitochondrial fitness using the approach of non-interacting mutants.

However, we think that the experiments of forced dimerization offer an excellent alternative to address the question about the role of BAX/DRP1 interaction. It is unique in that it enables temporal control over this process and a direct comparison of the status of each cell before and after treatment with the dimerizer. Thus, in the revised version of the manuscript, we have strengthened these experiments with additional controls and analysis. Please see our responses to the comments below and to reviewers 2 and 3. Given our observations that induced dimerization between BAX and DRP1, but not of the control samples, clearly induces apoptosis in absence of any apoptotic stimulus, the most reasonable and likely explanation is that the interaction has a pro-death role. Even if, besides this pro-death function by specific allosteric regulation that we propose based on our observations, DRP1 would contribute to BAX accumulation in mitochondria thus leading to autoactivation (hypothesis not supported by the controls of induced dimerization with TOM20 and non-interacting mutants of BAX), the outcome of the interaction is still pro-death. That said, we do not exclude that the BAX/DRP1 interaction may also play a role in signaling steps downstream of the initial activation and perhaps even cytochrome c and SMAC release, which are only now starting to become more evident, for example with the acknowledgement of mtDNA release. While not exempt of technical caveats, we think that our findings that the induced interaction between BAX and DRP1 leads to BAX activation and apoptosis provide the strongest evidence to date in support of the pro-death role of their interplay.

- The implication that DRP-1 may be a direct activator of BAX is really exciting. The control to imply that this is a specific event is that enforced dimerization of BAX with TOMM20 has no effect. In my opinion, this is subject to overinterpretation, we don't know what the topology of TOMM20 associated BAX is relative to DRP-1 associated BAX, it could be that TOMM20 associated BAX is distal from the mitochondrial membrane (relative to DRP-1 associated BAX) and therefore less likely to be active. A better control would be to carry out similar experiments using the BAX deletion mutants described earlier that can no longer bind DRP1, in this case does DRP-1 still activate these, killing cells?

We thank the reviewer for acknowledging the interest of our findings and for this suggestion.

Regarding the potential distance of BAX to the mitochondrial membrane when dimerized to TOM20 or to DRP1, we would like to mention that TOM20 is a 20kDa protein in which the N-terminus acts as a transmembrane anchor and only its C-

terminal residues 25-145 are cytoplasmic. It is laterally associated with the rest of the TOM complex, likely via weak interactions. In contrast, DRP1 is a larger protein of ~80kDa, only peripherally bound to mitochondria with membrane interactions via the variable domain and with the GTPase domain exposed to the cytosol. In our constructs, we added a fluorescent protein (FP) and the FRP/FKBP domains on the N-terminus next to the GTPase domain of DRP1 and on the C-terminus of TOM 20 (see answers to reviewer 2). Considering the current structural knowledge about these proteins (see figure R1 below), it is very unlikely that BAX is more proximal to mitochondria when dimerized to DRP1 than when dimerized to TOM20, which supports the use of TOM20 as a negative control.

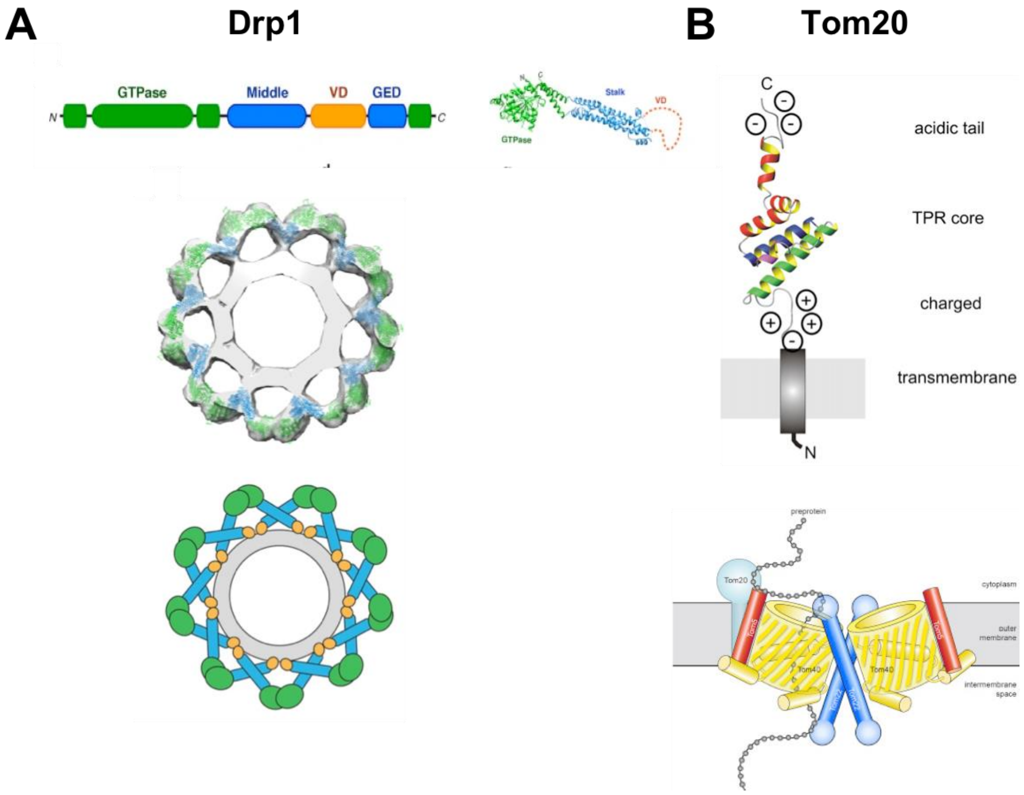

Figure R1. A) Top, domain scheme of DRP1 (left) and monomeric structure (right). Below, structural organization of DRP1 assembly in the membrane-bound conformation. Cryo-EM data in the center and corresponding scheme in the bottom. From DOI:10.1038/s41598-017-11008-3. The FKBP and FP in our constructs are fused to the GTPase domain (green). B) Structure of TOM20 (top, from DOI:10.1016/j.plaphy.2007.12.012, pdb ident: 1OM2) and scheme of the TOM complex (bottom, from DOI:10.1016/j.cell.2017.07.012).

Regarding the controls of induced dimerization with BAX mutants, as shown in the responses to reviewer 3, the mutants of BAX that do not interact with DRP1 have altered behavior (new Figure EV4A-C). They seem aggregated (BAXΔ19-37) or appear constitutively mitochondrial distributed in discrete foci (BAXL63E and BAXΔL1-2). Furthermore, for BAXL63E, mitochondria appear fragmented and stressed under resting conditions, which could also affect cell death sensitivity. In the cell death assay, the mutants lacking helix 1 or the loop between helices 1 and 2 exhibited a higher tendency to auto-activation compared to the wild type.

Considering these aspects and following the suggestion of this reviewer and reviewer 3, we have now performed the induced dimerization experiments with DRP1

analyzing only living cells expressing the non-interacting mutants of BAX, which are shown in Fig EV5E. Interestingly, forced dimerization of these mutants with DRP1 only recruited DRP1 to where BAX initially localized, thanks to the FRB/FKBP domains, but did not lead to major cellular alterations beyond those already induced by the expression of the mutants without dimerizer, compared to the wild type. For BAXΔL1-2, the foci-like distribution was increased in those cells that did not clearly have it before dimerization.

BAX N-terminus has been shown to participate in the initial steps of BAX activation by dislodging from the globular BCL-2 fold and allosterically favoring the exposure of the C-terminus and the binding of BH3 domains to the hydrophobic groove (for example, Dengler et al. CDD 2021, for BAK in Sandow JJ et al EMBO J 2021). We think that the N-terminus region acts as lock that restricts BAX activation and, upon mutation/deletion of the N-terminus, BAX more easily becomes activated. Based on our collective results in this study and the literature, our findings support a model in which DRP1 promotes BAX activation by favoring the dislodgement of the inhibitory N-terminus, now further strengthened by the new experiments.

- A key experiment to support the authors' hypothesis (DRP-1 can directly activate BAX) will be to carry out similar enforced dimerization experiments in cells lacking BH3-only (activator) proteins, HCT116 OCTA ko cells described elsewhere (PMID 27056669)

We thank the reviewer for this excellent idea. Following the reviewer's suggestion, we performed the BAX/DRP1 forced dimerization experiment in the HCT116 OCTA ko cell line and found that induced dimerization led to their translocation to mitochondria, accumulation into foci and loss of mitochondrial potential, as in the cell line containing BH3-only proteins. These results thereby indicate that no additional BH3-only proteins are required for BAX activation induced by interaction with DRP1, and strengthen the role of DRP1 as a non-BCL-2 activator of BAX. These experiments are now shown in Figure 6J,K.

Referee #2:

There is a long-lasting question in the apoptosis field whether the pro-fission Drp1 protein is actively involved in the apoptosis process. In this paper, Jenner et al elegantly demonstrate using sophisticated tools that Drp1 and the pro-apoptotic Bcl-2 family member Bax interact in lipid membranes and affect each other's activities in-vitro. They also mapped the interaction sites, and used live cell imaging to demonstrate that Bax and Drp1 co-localize at discrete foci during apoptosis. Moreover, Jenner et al demonstrate that enforced dimerization of Drp1 and Bax trigger their translocation to mitochondria, accumulation at discrete sites, mitochondria fragmentation, and depolarization of mitochondria.

We thank the reviewer for the positive evaluation of our work.

Thus, Jenner et al use many in-vitro tools and several tolls in intact cells - live cell imaging and enforced dimerization - to conclude that Drp1 interacts and activates Bax and induces apoptosis. Why does Bax require Drp1 if it has a super-efficient

activator such as truncated tBid, as shown by the authors with tBid (cBID) in Fig 3? Does Drp1 compete with tBid or with Bax itself on binding to Bax? On the other hand, why does Drp1 require Bax for mitochondria translocation if it has a super-efficient receptor like MFF? Does Bax compete with MFF?

This question is very interesting and perhaps even philosophical. Indeed, (partially) overlapping functions abound among the proteins of the BCL-2 family to regulate mitochondrial permeabilization in apoptosis and provide fine-tuning. Examples are BAX and BAK, or the existence of multiple activator BH3-only proteins, like BID, BIM and PUMA. Recent data also suggest that BH3-only proteins are not even completely required, as BAX and BAK can also autoactivate in absence of other BCL-2 proteins (O'Neil et al, Genes&Dev 2016). Adding DRP1 to the list of activators of BAX is not at odds with BH3-only activation of BAX, even if they are not equally efficient, and provides another level for regulation of BAX activity. This concept is now reinforced by the new induced dimerization experiments in HCT octaKO cells, which lack BH3-only proteins (Figure 6J-K), which we performed following the suggestion of reviewer 1.

The same applies to DRP1. It not only has MFF as a receptor, but also MDIV49/51 and maybe hFIS1, which are believed to fine-tune DRP1 activity in different cellular contexts. Even DRP1 alone is able to interact with membranes, at least in vitro. We think that BAX would act in addition to the known adaptors of DRP1 and provide an additional way of regulating its activity.

Despite these concerning issues, this reviewer is open to the authors' novel model that Drp1 plays an active role in Bax translocation/activation and vice versa, and if this model is correct, it will be an important contribution to our field. However, at this stage, this model is premature and there are several critical experiments to be performed in cells to ground it.

We thank the reviewer for recognizing the relevance of our work.

1) Do endogenous Drp1 and Bax directly interact in the intact mitochondrial membrane of apoptotic cells? This should be demonstrated by using cross-linkers as previously demonstrated for Bax homodimers/oligomers. Do Bax mutants that fail to cross-link to Drp1 in-vitro also fail to cross-link to Drp1 in intact mitochondria? Do these mutants possess reduced ability to induce apoptosis? I would also recommend using the Blue-native approach to strengthen this point.

This is a good point. We have made a big effort and explored different approaches to identify the interaction of endogenous BAX and DRP1 by pull-down, with and without cross-linkers as the reviewer suggested. Unfortunately, we could not find experimental conditions under which we could detect robust interaction between both proteins. We think that, without cross-linkers, the addition of detergents for sample preparation is likely to disrupt any interaction between BAX and DRP1, which requires the membrane environment. When we used cross-linkers, we detected bands reactive to BAX antibodies in the apoptotic samples pulled down with DRP1, but they migrated in the gel at a high molecular weight that so far we could not assign unequivocally with a BAX-specific band. We also think that the cross-linking reaction leads to the formation of very large complexes, in agreement with the high oligomer formation reported for both BAX and DRP1, that cannot be easily analyzed because

they may no longer enter the poly-acrylamide gel. While we think that these are common difficulties in the study of membrane protein complexes, the best we can do for now is to openly acknowledge this issue in the main text.

2) The Korsmeyer group has demonstrated in 1998 (Gross et al, EMBO J) that "Enforced dimerization of BAX results in its translocation, mitochondrial dysfunction and apoptosis". Have the Korsmeyers reached the wrong conclusion? Probably not. It probably depends on how the fusion proteins are constructed and in the case of Jenner et al the dimerization of their fusion proteins did not result in Bax activation. In any case, I think it is important to cite the Gross et al paper and discuss it.

We agree with the reviewer that how the constructs are designed can affect the outcome for BAX activation upon forced dimerization. In figure R2 we show a scheme of the constructs used in our study.

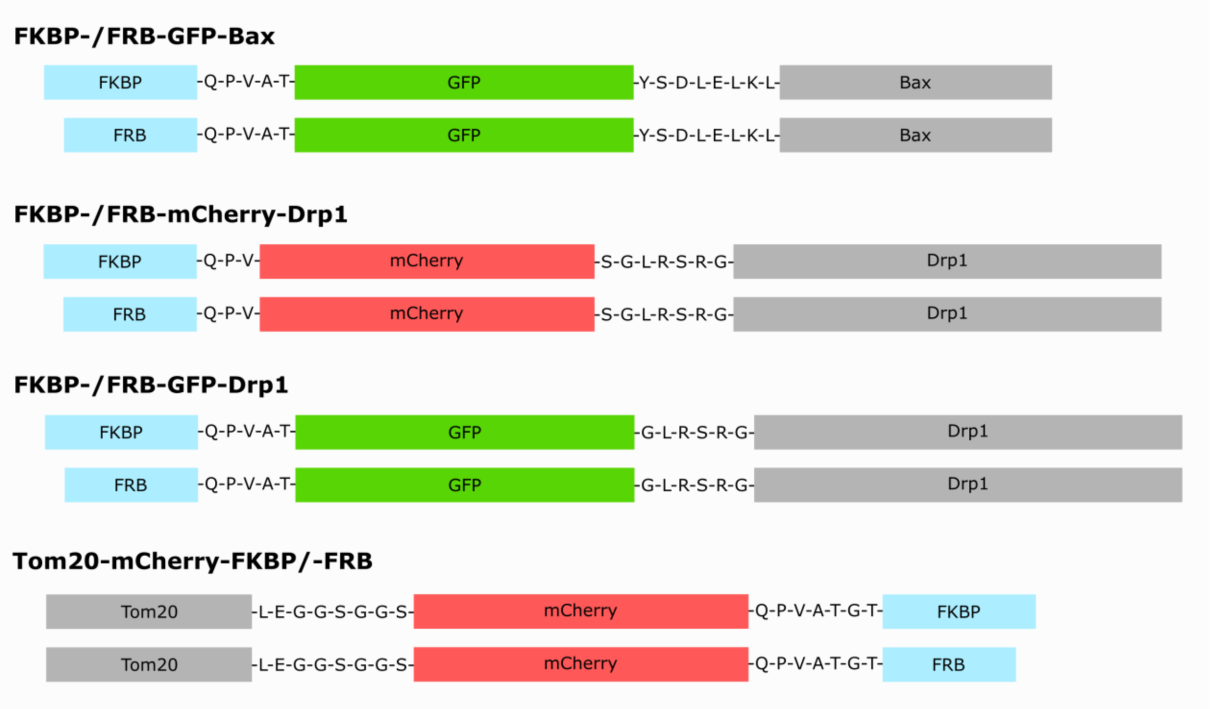

Figure R2. Scheme of the constructs used for the induced dimerization experiments.

In contrast to Gross et al, EMBO J 1998, where the FKBP/FRP domains where directly tagged on the N-terminus of BAX, our constructs include a FP that separates the protein from the dimerization domains. As a result, induction of BAX dimerization in Gross et al. likely brought closer association of the BAX molecules than in our experiments, which could more easily engage into auto-activating BH3-into-groove interactions, thereby leading to BAX activation, in contrast to our constructs. We have now cited the study by Gross et al. in the manuscript.

Moreover, to reach their conclusions, Gross at al used the following methods:
a) The FKBP/FK1012 dimerization system (and importantly FK506 to reverse the dimerization)
b) cross-linkers + Western blots
c) subcellular fractionations + Western blots (to monitor Bax translocation and cytochrome c release)

d) caspase activation assays
e) FACS analysis (to measure mitochondria membrane potential, reactive oxygen species (ROS) production and cell death)

Jenner at al should use similar approaches to prove that enforced dimerization of Bax and Drp1 results in their hetero-dimerization, translocation to mitochondria (using also Western blot analysis), N-terminal exposure of Bax, cyt c release, caspase activation, and FACS analysis to measure the parameters described in the Gross et al paper (and numerous additional papers). Jenner at al used MitoSpy NIR to measure mitochondria membrane potential and concluded that mitochondria depolarization is an indication of mitochondria outer membrane permeabilization (MOMP), which is incorrect.

We thank the reviewer for this suggestion. In the revised version of the manuscript, we have strengthened the evidence that forced BAX/DRP1 dimerization promotes apoptosis by confirming that it induces the translocation of BAX and DRP1 to mitochondria by Western Blot, that BAX is activated and exposes the 6A7 antigen by immunostaining, that cyt c is released by Western Blot, that mitochondria are depolarized by loss of MitoView staining, that caspases are activated with a caspase 3/7 assay, that PARP is cleaved by Western Blot and that cells die by Incucyte imaging. These new data are now shown in Figures 6F and EV5F-K.

3) Fig 1: In the ddFP experiments the authors are advised to measure the dimerization of Bax-Bax (RA-Bax and GB-Bax) and Bax-tBid (RA-Bax and GB-tBid) as positive references to assess the strength of the RA-Bax and GB-Drp1 dimerization. Why does DMSO inhibit the interaction between RA-Bax and GB-Drp1 (Fig 2C)?

Following the reviewer's suggestion, we have now added the interaction between RA-BAX and GB-BAX in Figure 1F-G.

With respect to the experiment in Figure 2C, rather that DMSO inhibiting the interaction between BAX and DRP1, what we observed is that CSA and caspase inhibitors allowed the formation of more foci, likely by decreasing and/or delaying cell death, respectively. We have now included the statistical analysis indicating that the differences are not significant.

4) Fig 2A: The representative pictures are confusing. The appearance of Drp1 and Bax interaction does not seem convincing since all the mitochondria are already collapsed. The increase in the intensity of Smac-GFP seems to come from signal accumulation on mitochondria, and not cytosol, of the dying cell, which seems to shine brighter. The experiments which include translocation of proteins from the cytosol to mitochondria should include markers for both compartments.

We understand the concerns of the reviewer. Unfortunately, we have not managed to obtain better movies of the process, because visualization of the spatiotemporal dynamics of the interaction between BAX and DRP1 in dying cells is technically very difficult. For example, one never knows when exactly which cell is going to die and also the process is quite fast, making it difficult to detect from the beginning and extremely time consuming. The purpose of Figure 2 is to illustrate the temporal

dynamics of the process with respect to SMAC release and we think that this is achieved.

That said, we provide a number of still images of cells in which the interaction between BAX and DRP1 is detected and where it can be clearly observed that the signal is not a by-product of mitochondrial collapse, see Figures 1E and EV1. If the reviewer thinks it is necessary, we can include more representative pictures in EV1.

For the calculation of the SMAC release, we quantified the background-corrected fluorescent intensity in a region of interest in the cytosol at different time points after apoptosis induction and we did not include any region with mitochondrial signal. The impression that the reviewer has from the images in Figure 2A is due to the intensity contrast between the signal in mitochondria and the cytosol, but it does not affect our calculations.

Regarding the labeling of the cytosolic and mitochondrial compartments, this becomes complicated if we want also to visualize in the same living cell the signal of the interaction between BAX and DRP1 and the signal of SMAC, since using 4 colors would create cross-talk problems. For these reasons, we generally collect the transmission image that allows to set the cell borders, and for mitochondria, we prefer to detect the organelle with mitoBFP in alternative experiments, as shown in Figure 1E and EV1.

5) On page 9 (2nd paragraph from top) the authors conclude that "These results suggest that the binding between BAX and DRP1 in apoptosis is independent of the membrane constriction function of DRP1, for which the GTPase activity is required". Thus, it is possible that the function of Drp1 on the post-fissed mitochondria is to recruit Bax and this is the reason why their interaction is independent of Drp1's GTPase activity.

While we cannot discard this option, BAX is still recruited to mitochondria and forms foci in absence of DRP1. So DRP1 may contribute to the recruitment, but it is not required for it. We have explained this in the text more clearly in p.10.

6) Figure 6, S1, S2, S5: add quantification of the images and statistics

Figure 1F, 2C, 2D, 3C, S3: mark statistically significant results and mention the level of significance in the legends

Figure 6D: mitochondrial fragmentation as well as mitochondrial membrane potential should be quantified

Figure 6E-G: perform co-localization analysis

We have corrected the figures accordingly.
For Fig 6E-G, we have performed a Pearson co-localization analysis which is now shown in Figure EV5B-D.

Referee #3:

In manuscript EMBOJ-2021-108587 entitled "Direct interaction with DRP1 activates BAX and induces apoptosis" Andreas Jenner, Ana Garcia-Saez and colleagues revisit earlier observations regarding the mitochondrial interplay of BAX, an effector of MOMP, and DRP1, a mitochondrial fission executioner, through a high magnification lens of super-resolution microscopy. The new approach (single molecule localization microscopy) confirms that these proteins colocalize within 30 nM (the size of about 7 side-by-side BAX molecules). The authors back up this observation by dissecting their interaction biochemically and through fluorescence microscopy in vitro and in cells. The authors conclude that the two proteins influence each other's membrane activities, with DRP1 promoting permeabilization of vesicles (LUVs and GUVs) by BAX, and BAX promoting accumulation of DRP1 to membranes. The authors clarify some of the previous observations supporting a direct interaction of DRP1 and BAX, yet they do not exclude previous observations suggesting that DRP1-mediated membrane remodeling recruits BAX. The authors propose that DRP1 acts as BAX activator by interacting with the N-terminal portion of BAX, including helix 1, helix 2 and the loop joining them. The interplay between BAX and DRP1 at the mitochondria is important to understand as it may directly influence the threshold for MOMP in apoptosis initiation in pathophysiology. Once BAX induces MOMP it may promote accumulation of DRP1 to induce mitochondrial fission and drive the biology downstream of MOMP. The manuscript uses elegant approaches, flows logically, is well written, and will be significant in elucidating the relationship between mitochondrial apoptosis and mitochondrial fission. The following comments are meant to help the authors clarify several aspects of the findings:

We thank the reviewer for acknowledging the significance of our study.

1. The authors used the dimerization dependent fluorescent protein (ddFP) technique to monitor BAX-DRP1 interaction with a resolution of 10 nM, stating on page 5: "As negative control, the combination of the anti-apoptotic BCL-2 protein BCL-xL tagged with RA and GB-DRP1 did not give any fluorescent signal neither in untreated nor in treated cells."
I could not find the data, but this negative control is very important and should be included in the manuscript.

Following the reviewer's suggestion, we have now included the images corresponding to this control in supplementary Figure EV1C.

2. The authors note delay in release of mitochondrial proteins through MOMP and appearance of BAX/DRP1 foci on page 6 related to Figure 2A and 2B: "The short delay between the redistribution of Smac-GFP and the appearance of BAX/DRP1 complexes is comparable to that observed with the formation of GFP-BAX foci and Smac release during apoptosis (Salvador-Gallego et al., 2016)." Figures 2A and 2B show that even at 270 min (timing in Figure 2A), when SMAC-GFP was almost completely released from the mitochondrial network (some perinuclear SMAC-GFP structure persist), there are no visible RA-BAX/GB-DRP1 foci. In Figure 2B it appears that foci are noted after 100 min. This implies that MOMP occurred but that the sensitivity of the ddFP assay precludes earlier detection of RA-BAX/GB-DRP1 foci. This discrepancy poses limitations in interpreting the functional consequences of BAX/DRP1 interactions at the earlier time points (<100 min). One way of overcoming this would be to identify DRP1 mutants that disrupt the

interaction with BAX and test their kinetics of association relative to SMAC-GFP release. This could be clarified in future research, but the authors need to acknowledge this limitation in the discussion. As it stands, during initial time points after apoptosis induction DRP1-BAX complex is "invisible" by microscopy and one cannot definitively conclude that the two proteins interact.

We apologize for this misunderstanding. The time points in Figure 2A are relative to the timing of the movie using treatment as time 0 and do not reflect the time differences between SMAC release and formation of BAX/DRP1 complexes. Also, unfortunately the times shown in Figures 2A and 2B did not correspond to each other. In Figure 2B, one should look at the time lag between the purple and the green curves, which is about 10 minutes average at 50% increase of both signals. To address this problem, we have now explained this better in the text and changed the relative timing of the experiments in 2A and 2B so that they correspond.

Following the reviewer’s suggestion and the concern of reviewer 1, we have also now explained better that there is this time difference of about 10 minutes in the detection of both events and how it limits the study. Although we agree with the reviewer that the lag time between the detection of SMAC release and the ddFP signal limits the interpretation of the functional relevance of BAX/DRP1 interaction in the context of the ddFP experiment, we would like to note that the experiments of induced dimerization provide additional support to this relevance in a complementary approach. Please see also answers to reviewer 1.

We thank the reviewer for suggesting to identify mutants of DRP1 that do not interact with BAX, which we found very interesting and thought it could also help answer one of the concerns of reviewer 1. Accordingly, we have designed several mutants of DRP1 that are affected at different stages during its mechanism of action and tested their ability to interact with BAX, which we show now in Figure 5F-H. Unfortunately, unlike with BAX, we could not identify any mutation in DRP1 that caused a clear-cut disruption of the interaction with BAX. In contrast to wild type DRP1 and the K38A mutant, which also produced ddFP signal at early apoptotic stages, the other DRP1 mutants gave rise to ddFP signal with BAX in discrete foci in close proximity of mitochondria that seemed at advanced stages of apoptotic induction. This suggests that localization to mitochondria is the most important requirement for DRP1 interaction with BAX. Since it was not feasible to perform a temporal analysis of the interaction for these DRP1 versions, we cannot discard that some of the mutants have indeed altered interaction at early time points of apoptosis. We have included these considerations in the revised text.

Minor comments related to Figure 2A and 2B: Time scales could be match with the x-axis at 0 in Figure 2B. It may be useful to include the 100 min image in Figure 2A.

We have now modified the time scales in the figure 2A for clarity.

3. The authors do not show the relative levels of expression (immunoblotting) for the different constructs and mutants investigated in Figure 5, nor their apoptotic activities. Presumably they have this data, which would be useful in drawing mechanistic conclusions. For instance, are h1 deletion, L1-2 deletions, and L63E active? (Some of the other deletions may be more disruptive to BAX fold relevant to

apoptosis function.) The full panel of constructs does not need to be investigated but it would be useful to test the three RA-BAX constructs functionally {plus minus} GB-DRP1.

We thank the reviewer for raising this point. We have now analyzed the levels of expression for the mutants BAXΔ19-37, BAXL63E and BAXΔL1-2. We also analyzed their localization and apoptotic activity. The new data are shown in Figure EV4A-C. While the bulk expression levels of some mutants were lower as seen by WB, they were all still clearly detectable in single cells by microscopy, indicating that they could be expressed. They seemed aggregated (BAXΔ19-37) or appeared constitutively mitochondrial distributed in discrete foci (BAXL63E and BAXΔL1-2). Furthermore, for BAXL63E, mitochondria appeared stressed under resting conditions, which could also affect cell death sensitivity. We also found that these mutants retained their ability to induce apoptosis. Indeed mutants BAXΔ19-37 and BAXΔL1-2, lacking helix 1 and the loop between helices 1 and 2, showed a higher tendency to auto-activation, likely explaining their lower levels detected by WB.

4. Related to point 3, if active, how do any of the three constructs impaired in DRP1 binding perform in apoptotic response upon forced dimerization as presented in Figure 6?

We thank the reviewer for this suggestion, which was also proposed by reviewer 1. We have now performed experiments of forced dimerization with these non-interacting BAX mutants, which are shown in Figure EV5E. Focusing our analysis on living cells, we found that forced dimerization of these mutants with DRP1 only recruited DRP1 to where BAX initially localized, thanks to the FRB/FKBP domains, but did not lead to major cellular alterations beyond those already induced by the expression of the mutants without dimerizer, compared to the wild type. For BAXΔL1-2, the foci-like distribution was increased in those cells that did not clearly have it before dimerization.

BAX N-terminus has been shown to participate in the initial steps of BAX activation by dislodging from the globular BCL-2 fold and allosterically favoring the exposure of the C-terminus and the binding of BH3 domains to the hydrophobic groove (for example, Dengler et al. CDD 2021, for BAK in Sandow JJ et al EMBO J 2021). We think that the N-terminus region acts as lock that restricts BAX activation and upon mutation/deletion of the N-terminus, BAX more easily becomes activated. Based on our collective results in this study and the literature, our findings support a model in which DRP1 promotes BAX activation by favoring the dislodgement of the inhibitory N-terminus, now further strengthened by the new experiments.

5. Vesicle permeabilization studies suggest that DRP1 does not activate BAX but that it cooperates with BID and heat activated BAX in permeabilization. Yet, the authors conclude that DRP1 may act by directly binding at a site similar to the noncanonical site engaged by BIM SAHB, based on the peptide array, deletion, and mutagenesis analyses. Could the authors include a cartoon model showing the sites on BAX that may be engaged by BID and DRP1 to make this more accessible to readership outside the field.

This is a good point. Our explanation is that the interaction between BAX and DRP1 only happens in the context of the membrane, which in the liposome assay in vitro

needs to be promoted by adding cBID or by using mild heat. This is in good agreement with the BAX activating antibody identified by the Kluck lab (doi: 10.1038/ncomms11734), which also binds to the N-terminus, but only activates specifically the mitochondrial fraction of BAX.

Following the reviewer's suggestion, we have now made a cartoon model where we have highlighted the binding site of the BH3 of BID and main the sites of DRP1 interaction based on our data. The model is shown in Figure EV4D.

Additional minor comments:

6. Pg 4. "Endogenous DRP1 was immunostained and labeled with the cyanine-based fluorescent (CF) dye CF680."
The authors could rewrite this sentence inferring that the secondary Ab was CF680 labeled.

We have now corrected this.

7. The authors need to move Figure 2D and associated supplementary figure later in the manuscript when this panel is discussed on pages 9-10 (possibly move to Supplementary Figure S5).

Following the reviewer's suggestion, we have now moved Figure 2D to new Figure EV4E-G.

8. Are BAX and DRP1 derivatized with AF probes on lysines or thiols? Please add these details in the methods. Derivatization at some sites may affect DRP1 binding and function.

We have now added this information to the manuscript. We have checked in previous studies that the labeling protocols used for BAX and DRP1 do not affect their activity (Bleicken et al. Biophys J, 2013; Ugarte-Uribe et al. JBC, 2014).

9. The authors should include the cross-correlation data in panel 3B for membrane+BID experiment in panel 3C.

Since in FCCS experiments it is very difficult that the Y axis is comparable for different individual GUVs, which makes the interpretation confusing, we have now included these data in Figure EV2C to address the reviewer's suggestion.

10. The authors need to mention what the rhodamine signal represents in Figure 3F.

Following the reviewer's suggestion, we have now added this information in the figure caption.

Dear Ana,

Thank you for submitting your revised manuscript to The EMBO Journal. Your study has now been seen by the original referees. As you can see below, the referees appreciate the introduced changes and support publication here.

Referee #3 has a few remaining concerns that I would like to ask you to take into consideration in a final revision.

When you submit the revised version will you also consider the following editorial points.

We are missing a Data Availability section - placed after the Materials and methods and before Acknowledgements This is the place to enter accession numbers etc. In case no data needs to be submitted to database please state: This study includes no data deposited in external repositories.

Reference list: for articles with more than 10 authors, the author list should be cut after 10 authors followed by et al.

Did you reuse Figure 1E in EV1A? If so please state that in the legend.

Our publisher has also done their pre-publication check on your manuscript. When you log into the manuscript submission system you will see the file "Data Edited Manuscript file". Look at the word file and the comments regarding the figure legends and respond to the issues.

We encourage the publication of source data, particularly for electrophoretic gels and blots, with the aim of making primary data more accessible and transparent to the reader. It would be great if you could provide me with a PDF file per figure that contains the original, uncropped and unprocessed scans of all or key gels used in the figure? The PDF files should be labelled with the appropriate figure/panel number and should have molecular weight markers; further annotation could be useful but is not essential. The PDF files will be published online with the article as supplementary "Source Data" files.

We include a synopsis of the paper (see http://emboj.embopress.org/). Please provide me with a general summary statement and 3-5 bullet points that capture the key findings of the paper.

We also need a summary figure for the synopsis. The size should be 550 wide by [200-400] high (pixels).

That should be all. Let me know if you have any further questions.

With best wishes

Karin

Karin Dumstrei, PhD
Senior Editor
The EMBO Journal

Guide For Authors: https://www.embopress.org/page/journal/14602075/authorguide

The revision must be submitted online within 90 days; please click on the link below to submit the revision online before 6th Feb 2022.

https://emboj.msubmit.net/cgi-bin/main.plex

Referee #1:

The authors have comprehensively addressed all my comments, clearly some experiments were not technically feasible (I appreciate the authors considerable efforts) but I think they convincingly demonstrate direct activation of BAX in a DRP1 dependent manner - this in itself is a major finding, implicating the possibility that other proteins can also have this effect.

Referee #2:

The authors have adequately addressed my comment.

I recommend the accept the MS

Referee #3:

The authors have addressed my comments satisfactorily. Even though the additional mutation analysis has not been fruitful due to the difficulty in pinpointing the interaction interface(s) at the resolution of this study, the authors acknowledge the need to perform future structural analysis of membrane associated BAX and Drp1 to resolve this dynamic complex at higher resolution, which should enable successful mutagenesis.

I want to bring a point that I previously missed, and which has not been emphasized by other reviewers related to the interaction of BAX-DC with Drp-1 presented in Figure 4A, B, D. Considering that BAX-DC is a very well behaved folded globular protein (used in vitro and structural analyses), one interpretation could be that Drp-1 interacts with the canonical hydrophobic groove of BAX promoting BAX targeting to the mitochondria also by competing with helix 9, which it also binds according to the peptide array. This point could be made in the discussion. I suggest inclusion of a non-structural model (beyond that shown in EV4D and more resembling a graphical abstract) to show the BAX structural elements that may be engaged by Drp-1, including the canonical hydrophobic groove which suggests possible temporal multi-site interactions that evolve as BAX changes conformation (i.e. Drp-1 binds FL BAX and then the hydrophobic groove to help target it and possibly even activate it through canonical interactions; this is essentially proposed for the BH3 activators that target the non-canonical site to allosterically pop helix 9 from the groove followed by canonical groove binding to activate BAX).

Minor:

In the discussion, authors should reference this manuscript (Lv F et al EMBO J. 2021 Jul 15;40(14):e106438. doi: 10.15252/embj.2020106438) related to the following statement:

"Although the conformation of DRP1 assemblies on constricted lipid tubes has been solved (Frohlich et al., 2013), we only have incomplete or low-resolution information about the structure of BAX in the membrane (Czabotar et al., 2013, Hauseman, Harvey et al., 2020, Salvador-Gallego et al., 2016)."

Typo in this discussion sentence, "bring" should be bringing
We now solve this conundrum by using artificial dimerizers, which provide temporal control of the interaction and a direct readout of the specific consequences of bring BAX and DRP1 together, which lead to BAX activation and apoptosis even in the absence of other BH3-only proteins.

POINT-BY-POINT ANSWERS TO THE REVIEWERS

Referee #1:

The authors have comprehensively addressed all my comments, clearly some experiments were not technically feasible (I appreciate the authors considerable efforts) but I think they convincingly demonstrate direct activation of BAX in a DRP1 dependent manner - this in itself is a major finding, implicating the possibility that other proteins can also have this effect.

We thank the reviewer for the appreciation of our work, as well as for acknowledging the relevance of our study.

Referee #2:

The authors have adequately addressed my comment.

I recommend the accept the MS

We thank the reviewer for the positive evaluation of our work.

Referee #3:

The authors have addressed my comments satisfactorily. Even though the additional mutation analysis has not been fruitful due to the difficulty in pinpointing the interaction interface(s) at the resolution of this study, the authors acknowledge the need to perform future structural analysis of membrane associated BAX and Drp1 to resolve this dynamic complex at higher resolution, which should enable successful mutagenesis.

We thank the reviewer for the evaluation and appreciation of our work.

I want to bring a point that I previously missed, and which has not been emphasized by other reviewers related to the interaction of BAX-DC with Drp-1 presented in Figure 4A, B, D. Considering that BAX-DC is a very well behaved folded globular protein (used in vitro and structural analyses), one interpretation could be that Drp-1 interacts with the canonical hydrophobic groove of BAX promoting BAX targeting to the mitochondria also by competing with helix 9, which it also binds according to the peptide array. This point could be made in the discussion. I suggest inclusion of a non-structural model (beyond that shown in EV4D and more resembling a graphical abstract) to show the BAX structural elements that may be engaged by

Drp-1, including the canonical hydrophobic groove which suggests possible temporal multi-site interactions that evolve as BAX changes conformation (i.e. Drp-1 binds FL BAX and then the hydrophobic groove to help target it and possibly even activate it through canonical interactions; this is essentially proposed for the BH3 activators that target the non-canonical site to allosterically pop helix 9 from the groove followed by canonical groove binding to activate BAX).

We thank the reviewer for this comment. We would like to point out that in Figure 4 we didn't investigate BAX-DC, but tested the ability of different peptide fragments covering the sequence of BAX to interact with DRP1. In addition, the in vitro experiments were carried out with full-length BAX. Regarding BAX-DC, we tested for interaction with DRP1 using ddFP in living cells (Figure 5D) and found that, despite showing reduced interaction compared to BAX wild type, the C-terminal region of BAX is not required for the interaction with DRP1. Considering our finding that BAX and DRP1 exclusively interact in the membrane, the reduced interaction of DRP1 with BAX-DC might result from the altered membrane association of BAX lacking the C-terminal membrane anchor.
In Figure 5D we show that deletion of helices α1, 2, 4, whose tertiary structure contributes to the hydrophobic groove of BAX, impaired the interaction with DRP1 (Fig 5D). However, considering the impact of deletion of one of these helices for the overall folding and function of BAX, this result doesn't provide a direct proof that DRP1 binds BAX in its hydrophobic groove. Since we lack the experimental evidence in support of the interaction model proposed by the reviewer, we decided not to discuss further this possibility in the manuscript.

Following the reviewer's suggestion, we have now included a non-structural model in EV6 showing a model for the interaction of BAX and DRP1 that triggers BAX activation and pore formation.

Minor:

In the discussion, authors should reference this manuscript (Lv F et al EMBO J. 2021 Jul 15;40(14):e106438. doi: 10.15252/embj.2020106438) related to the following statement: "Although the conformation of DRP1 assemblies on constricted lipid tubes has been solved (Frohlich et al., 2013), we only have incomplete or low-resolution information about the structure of BAX in the membrane (Czabotar et al., 2013, Hauseman, Harvey et al., 2020, Salvador-Gallego et al., 2016)."

We have now included this citation in the text.

Typo in this discussion sentence, "bring" should be bringing
We now solve this conundrum by using artificial dimerizers, which provide temporal control of the interaction and a direct readout of the specific consequences of bring BAX and DRP1 together, which lead to BAX activation and apoptosis even in the absence of other BH3-only proteins.

Thank you, we have corrected the typo.

3rd Revision - Editorial Decision 3rd Dec 2021

Dear Ana,

Thanks for submitting your revised manuscript to The EMBO Journal. I have now had a chance to take a look at the revised version and all looks good!

I am therefore very pleased to accept the manuscript for publication here.

Congratulations on a nice study!

Best Karin

Karin Dumstrei, PhD
Senior Editor
The EMBO Journal

Please note that it is EMBO Journal policy for the transcript of the editorial process (containing referee reports and your response letter) to be published as an online supplement to each paper. If you do NOT want this, you will need to inform the Editorial Office via email immediately. More information is available here:
https://www.embopress.org/page/journal/14602075/authorguide#transparentprocess

Your manuscript will be processed for publication in the journal by EMBO Press. Manuscripts in the PDF and electronic editions of The EMBO Journal will be copy edited, and you will be provided with page proofs prior to publication. Please note that supplementary information is not included in the proofs.

Please note that you will be contacted by Wiley Author Services to complete licensing and payment information. The 'Page Charges Authorization Form' is available here: https://www.embopress.org/pb-assets/embo-site/tej_apc.pdf

Should you be planning a Press Release on your article, please get in contact with embojournal@wiley.com as early as possible, in order to coordinate publication and release dates.

If you have any questions, please do not hesitate to call or email the Editorial Office. Thank you for your contribution to The EMBO Journal.

** Click here to be directed to your login page: https://emboj.msubmit.net

EMBO PRESS

YOU MUST COMPLETE ALL CELLS WITH A PINK BACKGROUND

PLEASE NOTE THAT THIS CHECKLIST WILL BE PUBLISHED ALONGSIDE YOUR PAPER

Corresponding Author Name: Ana J. Garcia-Saez
Journal Submitted to: EMBO Journal
Manuscript Number: EMBOJ-2021-108587

Reporting Checklist For Life Sciences Articles (Rev. June 2017)

This checklist is used to ensure good reporting standards and to improve the reproducibility of published results. These guidelines are consistent with the Principles and Guidelines for Reporting Preclinical Research issued by the NIH in 2014. Please follow the journal's authorship guidelines in preparing your manuscript.

A- Figures

1. Data

The data shown in figures should satisfy the following conditions:

- the data were obtained and processed according to the field's best practice and are presented to reflect the results of the experiments in an accurate and unbiased manner.
- figure panels include only data points, measurements or observations that can be compared to each other in a scientifically meaningful way.
- graphs include clearly labeled error bars for independent experiments and sample sizes. Unless justified, error bars should not be shown for technical replicates.
- if n< 5, the individual data points from each experiment should be plotted and any statistical test employed should be justified
- Source Data should be included to report the data underlying graphs. Please follow the guidelines set out in the author ship guidelines on Data Presentation.

2. Captions

Each figure caption should contain the following information, for each panel where they are relevant:

- a specification of the experimental system investigated (eg cell line, species name).
- the assay(s) and method(s) used to carry out the reported observations and measurements
- an explicit mention of the biological and chemical entity(ies) that are being measured.
- an explicit mention of the biological and chemical entity(ies) that are altered/varied/perturbed in a controlled manner.

- the exact sample size (n) for each experimental group/condition, given as a number, not a range;
- a description of the sample collection allowing the reader to understand whether the samples represent technical or biological replicates (including how many animals, litters, cultures, etc.).
- a statement of how many times the experiment shown was independently replicated in the laboratory.
- definitions of statistical methods and measures:
  - common tests, such as t-test (please specify whether paired vs. unpaired), simple χ^2 tests, Wilcoxon and Mann-Whitney tests, can be unambiguously identified by name only, but more complex techniques should be described in the methods section;
  - are tests one-sided or two-sided?
  - are there adjustments for multiple comparisons?
  - exact statistical test results, e.g., P values = x but not P values < x;
  - definition of 'center values' as median or average;
  - definition of error bars as s.d. or s.e.m.

Any descriptions too long for the figure legend should be included in the methods section and/or with the source data.

In the pink boxes below, please ensure that the answers to the following questions are reported in the manuscript itself. Every question should be answered. If the question is not relevant to your research, please write NA (non applicable). We encourage you to include a specific subsection in the methods section for statistics, reagents, animal models and human subjects.

B- Statistics and general methods

Please fill out these boxes (Do not worry if you cannot see all your text once you press return)

1.a. How was the sample size chosen to ensure adequate power to detect a pre-specified effect size?	Sample size was chosen using a target variance that would allow to detect differences between the samples using a t-test with at least 95% confidence.
1.b. For animal studies, include a statement about sample size estimate even if no statistical methods were used.	N/A
2. Describe inclusion/exclusion criteria if samples or animals were excluded from the analysis. Were the criteria pre-established?	N/A
3. Were any steps taken to minimize the effects of subjective bias when allocating animals/samples to treatment (e.g. randomization procedure)? If yes, please describe.	N/A
For animal studies, include a statement about randomization even if no randomization was used.	N/A
4.a. Were any steps taken to minimize the effects of subjective bias during group allocation or/and when assessing results (e.g. blinding of the investigator)? If yes please describe.	The microscopy images of SMLM, of ddFP, of DRP1 binding to GUVs were analyzed and quantified in a blinded fashion. No special measures were considered for the rest of the experiments.
4.b. For animal studies, include a statement about blinding even if no blinding was done	N/A
5. For every figure, are statistical tests justified as appropriate?	Yes
Do the data meet the assumptions of the tests (e.g., normal distribution)? Describe any methods used to assess it.	Due to the nature of the data and analysis, we did not assess this.
Is there an estimate of variation within each group of data?	N/A

USEFUL LINKS FOR COMPLETING THIS FORM

http://www.antibodypedia.com
http://1degreebio.org
http://www.equator-network.org/reporting-guidelines/improving-bioscience-research-repor
http://grants.nih.gov/grants/olaw/olaw.htm
http://www.mrc.ac.uk/Ourresearch/Ethicsresearchguidance/Useofanimals/index.htm
http://ClinicalTrials.gov
http://www.consort-statement.org
http://www.consort-statement.org/checklists/view/32-consort/66-title
http://www.equator-network.org/reporting-guidelines/reporting-recommendations-for-tumo
http://datadryad.org
http://figshare.com
http://www.ncbi.nlm.nih.gov/gap
http://www.ebi.ac.uk/ega
http://biomodels.net/
http://biomodels.net/miriam/
http://jjj.biochem.sun.ac.za
http://oba.od.nih.gov/biosecurity/biosecurity_documents.html
http://www.selectagents.gov/

Is the variance similar between the groups that are being statistically compared?	N/A

C- Reagents

6. To show that antibodies were profiled for use in the system under study (assay and species), provide a citation, catalog number and/or clone number, supplementary information or reference to an antibody validation profile. e.g., Antibodypedia (see link list at top right), 1DegreeBio (see link list at top right).	done
7. Identify the source of cell lines and report if they were recently authenticated (e.g., by STR profiling) and tested for mycoplasma contamination.	We do not have this information at the moment.

* for all hyperlinks, please see the table at the top right of the document

D- Animal Models

8. Report species, strain, gender, age of animals and genetic modification status where applicable. Please detail housing and husbandry conditions and the source of animals.	N/A
9. For experiments involving live vertebrates, include a statement of compliance with ethical regulations and identify the committee(s) approving the experiments.	N/A
10. We recommend consulting the ARRIVE guidelines (see link list at top right) (PLoS Biol. 8(6), e1000412, 2010) to ensure that other relevant aspects of animal studies are adequately reported. See author guidelines, under 'Reporting Guidelines'. See also: NIH (see link list at top right) and MRC (see link list at top right) recommendations. Please confirm compliance.	N/A

E- Human Subjects

11. Identify the committee(s) approving the study protocol.	N/A
12. Include a statement confirming that informed consent was obtained from all subjects and that the experiments conformed to the principles set out in the WMA Declaration of Helsinki and the Department of Health and Human Services Belmont Report.	N/A
13. For publication of patient photos, include a statement confirming that consent to publish was obtained.	N/A
14. Report any restrictions on the availability (and/or on the use) of human data or samples.	N/A
15. Report the clinical trial registration number (at ClinicalTrials.gov or equivalent), where applicable.	N/A
16. For phase II and III randomized controlled trials, please refer to the CONSORT flow diagram (see link list at top right) and submit the CONSORT checklist (see link list at top right) with your submission. See author guidelines, under 'Reporting Guidelines'. Please confirm you have submitted this list.	N/A
17. For tumor marker prognostic studies, we recommend that you follow the REMARK reporting guidelines (see link list at top right). See author guidelines, under 'Reporting Guidelines'. Please confirm you have followed these guidelines.	N/A

F- Data Accessibility

18: Provide a "Data Availability" section at the end of the Materials & Methods, listing the accession codes for data generated in this study and deposited in a public database (e.g. RNA-Seq data: Gene Expression Omnibus GSE39462, Proteomics data: PRIDE PXD000208 etc.) Please refer to our author guidelines for 'Data Deposition'.<br><br>Data deposition in a public repository is mandatory for:<br>a. Protein, DNA and RNA sequences<br>b. Macromolecular structures<br>c. Crystallographic data for small molecules<br>d. Functional genomics data<br>e. Proteomics and molecular interactions	N/A
19. Deposition is strongly recommended for any datasets that are central and integral to the study; please consider the journal's data policy. If no structured public repository exists for a given data type, we encourage the provision of datasets in the manuscript as a Supplementary Document (see author guidelines under 'Expanded View' or in unstructured repositories such as Dryad (see link list at top right) or Figshare (see link list at top right).	N/A
20. Access to human clinical and genomic datasets should be provided with as few restrictions as possible while respecting ethical obligations to the patients and relevant medical and legal issues. If practically possible and compatible with the individual consent agreement used in the study, such data should be deposited in one of the major public access-controlled repositories such as dbGAP (see link list at top right) or EGA (see link list at top right).	N/A
21. Computational models that are central and integral to a study should be shared without restrictions and provided in a machine-readable form. The relevant accession numbers or links should be provided. When possible, standardized format (SBML, CellML) should be used instead of scripts (e.g. MATLAB). Authors are strongly encouraged to follow the MIRIAM guidelines (see link list at top right) and deposit their model in a public database such as Biomodels (see link list at top right) or JWS Online (see link list at top right). If computer source code is provided with the paper, it should be deposited in a public repository or included in supplementary information.	N/A

G- Dual use research of concern

22. Could your study fall under dual use research restrictions? Please check biosecurity documents (see link list at top right) and list of select agents and toxins (APHIS/CDC) (see link list at top right). According to our biosecurity guidelines, provide a statement only if it could.	No
